# Substantiating chemical groups for read-across using molecular response profiles

**DOI:** 10.1016/j.yrtph.2025.105894

**Published:** 2025-06-26

**Authors:** Rosemary E. Barnett, Thomas N. Lawson, Claudia Rivetti, Carlos Barata, Mark T.D. Cronin, Silvia Lacorte, Gavin R. Lloyd, Ralf J.M. Weber, Matthew J. Smith, Andrew D. Southam, Adam Biales, Kara Koehrn, Bruno Campos, John K. Colbourne, Geoff Hodges, Mark R. Viant

**Affiliations:** a Michabo Health Science Limited, Union House, 111 New Union Street, Coventry, CV1 2NT, U.K.; b Unilever, Safety and Environmental Assurance Centre (SEAC), Colworth Science Park, Sharnbrook, MK44 1LQ, U.K.; c Department of Environmental Chemistry, Institute of Environmental Assessment and Water Studies (IDAEA), C. Jordi Girona 18-26, 08034, Barcelona, Spain; d School of Pharmacy and Biomolecular Sciences, Liverpool John Moores University, Byrom Street, Liverpool, L3 3AF, U.K.; e Phenome Centre Birmingham, University of Birmingham, Edgbaston, Birmingham, B15 2TT, U.K.; f U.S. Environmental Protection Agency Office of Research and Development, Center for Computational Toxicology and Exposure, 26 W. Martin Luther King Dr., Cincinnati, OH, 45268, U.S.A.; g U.S. Environmental Protection Agency Office of Pollution Prevention and Toxics, 1200 Pennsylvania Avenue NW, Washington, DC, 20460, U.S.A.

**Keywords:** Grouping, Read-across, Bioactivity similarity, Plausible toxicological interpretation, Transcriptomics, Metabolomics, Hierarchical clustering, Replicability confidence, Daphnia

## Abstract

By grouping structurally similar chemicals, toxicity endpoints from data-rich substances can be read across to data-poor substances, supporting environmental and human health risk assessment without animal testing. However, structural similarity alone is insufficient, and additional supporting data can strengthen a grouping justification. This study aimed to demonstrate how multi-omics bioactivity data can increase confidence in a grouping hypothesis, where the bioactivity profiles can reflect a chemical’s mode(s) of action. We investigated three structurally similar phthalates and three uncouplers of oxidative phosphorylation, applying structure-based grouping approaches and short-term exposures of the ecotoxicological test species *Daphnia magna* to generate multi-omics data. Bioactivity similarities between the ‘omics responses to chemical exposure were assessed using t-statistics comparing treated samples to controls and visualised using hierarchical cluster analysis. Conventional structure-based grouping did not assign the phthalates and uncouplers into two anticipated categories, with the structurally more diverse uncouplers often assigned into multiple groups. Following bioactivity thresholding, which removed one uncoupler as it induced minimal molecular responses, bioactivity profile-based grouping of the remaining five substances correctly separated them into two chemical classes with high replicability confidence. However, a plausible toxicological interpretation of the reduced set of functionally annotated molecular features driving the grouping was attempted, although of limited success. This study demonstrates how multi-omics bioactivity profiles can increase confidence in chemical grouping and investigates a potential strategy for plausibly interpreting ‘omics data.

## 1. Introduction

Chemical grouping and read-across assist regulatory agencies and chemical companies in their evaluation of environmental and human health hazards posed by chemicals while reducing or even avoiding the need for animal testing (Bishop et al., 2012). Under U.S. and European regulations, read-across of toxicity endpoint data from one (source) substance to another (target) substance for which data are not available is permitted, which can avoid the need for additional animal studies. Guidance on how to construct and report a grouping and read-across argumentation is available from the European Chemicals Agency (ECHA; Read-Across Assessment Framework [RAAF]; ECHA, 2017), the Organization for Economic Co-operation and Development (OECD; OECD, 2017), and the European Centre for Ecotoxicology and Toxicology of Chemicals (ECETOC; ECETOC, 2012). A key prerequisite for any read-across argument is the grouping hypothesis on which it is based. Examples include the (bio)transformation of source and target substances to a common compound, or the structural similarity of test substances that infers they will cause similar toxicities, which can (ideally) be explained at a mechanistic level (RAAF, 2017). Thus, structural similarity is a fundamental part of any read-across argument (Kuseva et al., 2019). However, the RAAF stipulates that structural similarity alone is not sufficient to justify read-across, and that it should be linked to an explanation as to how and why the prediction is possible.

Read-across cases often have to be rejected by ECHA, in part due to incomplete supporting data to justify the grouping hypothesis and/or to demonstrate scientific plausibility. This can include a lack of evidence of the likely modes of action (MoA; Ball et al., 2016). One method to strengthen a read-across justification is to group chemicals using biological-effects data from targeted *in vitro* assays representing key mechanisms of endpoint toxicity (Escher et al., 2019). A second approach is to use biological-effects data from ‘omics technologies, such as transcriptomics and metabolomics, which measure broad ranges of molecular responses and potentially provide insights into a chemical’s MoA (Brockmeier et al., 2017; Pestana et al., 2021). Recently, a workflow has been proposed for chemical grouping using ‘omics data, comprising five steps: (i) design the study, providing a rationale for selecting the biological test system and ‘omics technology, here focused on ecotoxicology; (ii) acquire ‘omics data and associated metadata; (iii) group substances based on a statistical assessment of the bioactivity similarities of the ‘omics responses; (iv) attempt to provide a plausible toxicological interpretation of the molecular responses induced by each group of similar chemicals; and (v) integrate the findings into an analogue or category justification (Viant et al., 2024). Previously, we reported the use of bioactivity profile-based grouping to identify the most reliable source substance from a pool of six potential azo dye analogues, by combining transcriptomics and metabolomics data, and then reading across a reproductive toxicity endpoint to the target substance (Gruszczynska et al., 2024). Although a subsequent toxicity study supported the reproductive toxicity read-across prediction, one limitation of that study was the selection and investigation of data-poor substances in an invertebrate model for which the MoA(s) were unknown. Consequently, the grouping hypothesis derived from the ‘omics data could not be evaluated against prevailing toxicological knowledge.

The current study aims to further demonstrate the value of multi-omics measurements in bioactivity profile-based grouping to strengthen a grouping justification, including the first steps towards plausible toxicological interpretations of the molecular data that may reflect the MoA. We focus on six relatively data-rich test substances that we hypothesise will form two distinct chemical categories, the first comprising three uncouplers of oxidative phosphorylation (in both vertebrates and invertebrates; Song and Villeneuve, 2021): 2,3,4,5-tetrachlorophenol (TCP), carbonyl cyanide 3-chlorophenylhydrazone (CCCP) and carbonyl cyanide 4-(trifluoromethoxy)phenylhydrazone (FCCP). These substances are hydrophobic, protonophoric weak acids that uncouple phosphorylation from electron transport by dissipating the pH gradient within mitochondria (Terada, 1990; Hawliczek-Ignarski et al., 2017). It should be noted, from a structural perspective, that only two of these three uncouplers would be regarded as sufficiently similar to be considered as analogues in a formal regulatory read-across case. The second group comprised three structurally-similar phthalates: benzyl butyl phthalate (BBP), dibutyl phthalate (DBP) and diisobutyl phthalate (DiBP). Although some phthalates fit the World Health Organization’s definition of endocrine disruptors for their effects in vertebrates (Sohn et al., 2016; Xu et al., 2020; Czubacka et al., 2021; Sedha et al., 2021), their MoA in invertebrates is largely unknown, although chronic exposure to diethyl phthalate or DBP has been shown to increase fat accumulation and reduce lifespan in *Daphnia* (Seyoum and Pradhan, 2019). Here we hypothesise that the high structural similarities of these phthalates will lead to similar molecular responses in invertebrates.

Our approach comprises three primary objectives: first, to apply a range of conventional approaches to formulate a grouping hypothesis for the six substances, including structural similarity-based grouping and an array of mode/mechanism of action profilers. Second, to group the substances based on bioactivity profile data to statistically derive the bioactivity similarities among the substances, thereby attempting to substantiate the structure-based grouping hypothesis (Viant et al., 2024). Bioactivity profile data are obtained from the omics responses of *Daphnia magna*, acting as an environmental biosensor and having direct relevance to regulations that protect the environment, as well as satisfying the 3Rs principles of reducing, refining or replacing vertebrate animal testing to measure systemic toxicity. Third, we sought a plausible toxicological interpretation of the bioactivity profile data that combines transcriptomics and metabolomics to build greater confidence in the omics-based grouping. Based on our new findings, this objective was extended to tentatively explore the conservation of molecular effects across animal species to determine whether the bioactivity profile-based grouping in *Daphnia* may be more broadly applicable to other environmental species. We have shown previously that many toxicologically relevant pathways are evolutionarily conserved, thereby providing a basis for reading across toxicological interpretations from *Daphnia* to distantly related animals, including fish (Colbourne et al., 2022).

## 2. Materials and methods

### 2.1. Test substances

Benzyl butyl phthalate (BBP; CASRN 85-68-7), dibutyl phthalate (DBP; CASRN 84-74-2), diisobutyl phthalate (DiBP; CASRN 84-69-5), 2,3,4,5-tetrachlorophenol (TCP; CASRN 4901-51-3), carbonyl cyanide 3-chlorophenylhydrazone (CCCP; CASRN 555-60-2) and carbonyl cyanide 4-(trifluoromethoxy)phenylhydrazone (FCCP; CASRN 370-86-5) were purchased from Sigma-Aldrich (UK) with a purity ≥95 %.

### 2.2. Structural similarity

Using the *pvclust* package (version 2.2–0; Suzuki and Shimodaira, 2006), each pair of ToxPrint chemotypes (one per substance, from Hazard Comparison Database (https://hazard.sciencedataexperts.com/; Yang et al., 2015) were compared using binary distance (method.dist = “binary”) and hierarchical clustering (method.hclust = “ward.D2”) with multiscale bootstrap resampling (n = 10,000 bootstrap pseudo-replications). Other structural fingerprints were assessed using the same approach (Supplemental section S1 and Fig. S1).

### 2.3. Mode/mechanism of action profilers

A consensus approach was taken to help identify a MoA from readily available structure-based classifiers. Classification outputs were derived from the scheme of Russom et al. (1997), available via Chemprop (v7.1.1) and the US EPA Toxicity Estimation Software Tool (TEST) (U.S. EPAUser Guide for T.E.S.T, 2016) (v5.1.2). The following profilers were applied in the OECD QSAR Toolbox (OECD, 2020) (ver 4.4.1): Acute Aquatic Toxicity MOA by OASIS (AAT OASIS) (v3.4), Uncouplers (MITOTOX) (v1.1), Estrogen Receptor Binding (v2.2), the rtER Expert System – USEPA (v1.0) and Verhaar Scheme and adaptations ((Verhaar et al., 1992; Verhaar et al., 2000; Enoch et al., 2008) (v3.2)). Additionally, more recent classifiers were also applied, based on the identification of mechanisms of action. The schemes of Sapounidou-Firman (Sapounidou et al., 2021; Firman et al., 2022) and also MechoA (Bauer et al., 2018a,b; iSafeRat^®^ MechoA profiler v1.1.2 (KREATiS, 2020) (May 2023), which includes MechoA scheme v2.2 (October 2020)), were used.

### 2.4. Daphnia acute toxicity and benchmark dose modelling

*Daphnia* were maintained under constant environmental conditions (20 ± 1 °C; 16:8 h light:dark photoperiod) in water drawn from a borehole at the University of Birmingham. Cultures (20 *Daphnia* L^−1^) were fed daily with a suspension of *Chlorella vulgaris* corresponding to 0.08 mgC *Daphnia*^−1^. The acute (48 h) toxicity (immobilisation) of test substances was established in line with OECD guideline 202 (OECD, 2004) for DiBP (0.01–10 mg L^−1^), DBP (0.001–10 mg L^−1^), BBP (0.5–14 mg L^−1^), TCP (0.01–10 mg L^−1^), CCCP (0.001–0.4 mg L^−1^) and FCCP (0.001–10 mg L^−1^). Dimethyl sulfoxide (DMSO) was used as carrier solvent for all test substances and untreated controls (final DMSO concentration of 0.1 %). Benchmark dose (BMD) modelling using the PROAST webtool (https://proastweb.rivm.nl/version 66.39) was applied to 48 h immobilisation data (Fig. S2) to derive BMD estimates for 10 % immobilisation (Table S2) and which informed dose levels for multi-omics exposures (Table S3).

### 2.5. Daphnia exposures for multi-omics sampling

For multi-omics samples, *D. magna* were obtained from cultures <24 h old and grown at an increased density of 40 *Daphnia* L^−1^ with daily feeding for 4 d. Exposures were initiated at 5 d, where test organisms were pooled and randomly distributed into exposure beakers (100 mL) containing 10 individuals (*n* = 6 beakers per treatment). Exposures were conducted in two batches, with the lower estimate of benchmark dose for 10 % immobilisation after 48 h (BMDL) used as high exposure dose, the medium dose being one third BMDL, and low dose one ninth BMDL. The first batch included five compounds (BBP (0.51, 1.53, 4.58 μM), DBP (0.43, 1.30, 3.92 μM), DiBP (3.16, 9.48, 28.45 μM), FCCP (0.10, 0.29, 0.86 μM), and TCP (0.47, 1.40, 4.20 μM)) with shared solvent controls (0.1 % DMSO), and the second batch was conducted with CCCP (0.01, 0.03, 0.09 μM) and paired solvent controls. This second batch was required due to greater mortality in CCCP-treated animals at the original concentrations (0.02, 0.06, 0.19 μM) than anticipated, with the repeat exposures using half the initial concentration (Table S3); the repeat exposures proceeded as expected, with 14 % immobilisation observed in the high dose treatment after 48 h, close to the target value of 10 % expected for this dose). Samples were collected following 24 h (high dose) and 48 h (all dose groups) by filtration of organisms from the test media, rinsing with deionised H_2_O and flash-freezing in liquid nitrogen. Frozen *Daphnia* tissue was homogenised in methanol/water (71.4/28.6, v/v) extraction solvent (448 μL) using a bead-based homogeniser (Precellys-24 with CK14 homogenisation tubes, Stretton Scientific, UK) and split for RNA (10 %, equivalent to 45 μL homogenate, flash-frozen and stored at −80 °C for further transcriptomics preparation) and metabolite extraction (90 %, extracted immediately).

### 2.6. Analytical determination of exposure concentrations

Test substance concentrations were measured in media samples from the *Daphnia* exposure studies, collected at 0, 24 and 48 h, as detailed in Supplemental section S2 and Table S4. In brief, samples were analysed using liquid chromatography coupled to a triple quadrupole mass analyser (LC–MS/MS) with an electrospray ion source (Xevo TQD, Waters), and a BEH C18 analytical column (Acquity, Waters). Identification criteria included the retention time and two transitions, one used for quantification and the other for confirmation. Samples were spiked with isotope-labelled surrogate standards for quantification purposes.

### 2.7. Metabolomics data acquisition and processing

Metabolomics analyses are described in Supplemental section S3. In brief, metabolites were extracted using a biphasic method modified from Southam et al. (2021), producing dried polar and non-polar (i.e., lipophilic compounds) extracts from each sample. Data were acquired using ultra-high-performance liquid chromatography mass spectrometry (UHPLC–MS) metabolomics, with a Dionex UltiMate 3000 Rapid Separation LC coupled with a heated electrospray Q Exactive Focus mass spectrometer (Thermo Scientific), using both HILIC and C18 columns. A hybrid metabolomics method was used, including both an untargeted analysis and measuring multiple metabolic biomarkers from the MTox700+ panel (Sostare et al., 2022). Changes in the levels of several thousand polar metabolite and lipophilic features were determined (Lloyd et al., 2020), and features were identified using UHPLC–MS/MS, an in-house metabolite library, and the Galaxy Deep Metabolome Annotation (DMA) computational workflow.

### 2.8. Transcriptomics data acquisition and processing

Sample homogenates were pelleted (15,000-*g*, 5 min, 4 °C) and extracted with a Biomek FXp liquid handling robot (Beckman Coulter) using the Agencourt RNAdvance Tissue Kit (Beckman Coulter A32646) according to the manufacturer’s protocol. Purified RNA concentration and RNA integrity number were determined with a Nanodrop-8000 spectrophotometer (Thermo Fisher ND-8000-GL) and Agilent Tapestation 2200 (Agilent G2964AA) with high sensitivity RNA screentapes (Agilent 5067-5579). Changes in gene expression were assessed using a custom-designed, targeted TempO-Seq^®^ assay, consisting of 2378 probes of a BioSpyder platform covering 1988 *D. magna* genes mapped to human orthologs as described in Gruszczynska et al. (2024). Samples were shipped as extracted RNA with the remaining sample preparation and sequencing performed by BioClavis (UK). Raw counts were summarised to gene level after the removal of probes with aberrant hybridisation (*n* = 7), normalised and log-transformed using *DESeq2* (version 1.30.0; Love et al., 2014).

### 2.9. Bioactivity profile-based grouping

Two sets of statistical analyses were applied to the transcriptomics (single matrix) and metabolomics datasets (four matrices, comprising positive and negative ion analysis of polar metabolites and lipophilic compounds). First, Student’s t-tests (adjusted for false discovery rate; *q* < 0.1) were applied across all molecular features using normalised matrices to identify any significantly changing features between treated dose/time groups and their time-matched solvent controls. These results indicated the potency of each substance. Next, the high dose treatment group (and control) multi-omics data were prepared for hierarchical cluster analysis (HCA) to group the substances based on bioactivity similarities. Specifically, for each feature, the highest absolute t-statistic across the two time points (‘maximum-perturbation’ approach; Gruszczynska et al., 2024) was selected and vector-normalised (converted to a unit vector for each treatment condition). The features from all five ‘omics assays together served as input data for HCA, utilising a Euclidean distance metric and Ward’s linkage method (Murtagh and Legendre, 2014) implemented in *pvclust* (version 2.2–0; Suzuki and Shimodaira, 2006). Bootstrap replicability confidence *p*-values for the chemical grouping (10,000 bootstrap replications) were computed using the selective inference method (Shimodaira and Terada, 2019). The molecular features driving the grouping were discovered using partial least squares-discriminant analysis (PLS-DA). First, feature fold-changes were calculated for the relevant treated samples (i.e., high dose, at the same time point used in the HCA, for consistency) by dividing each sample’s feature intensity (metabolite level or gene expression) by the median of the corresponding control group. PLS–DA was applied to this fold-change dataset, with the earlier HCA results used to define the group membership.

### 2.10. Cross-species conservation of molecular pathways

The 1988 *D. magna* genes represented by probes on the BioSpyder platform were identified as members of evolutionarily conserved gene families among invertebrates and vertebrates, including humans (Colbourne et al., 2022). In brief, the mapping of each *Daphnia* gene to their orthologs in humans and other animals was obtained by a SPARQL query within the OrthoDB Database v10 (Kriventseva et al., 2019), which retrieved all genes forming gene families that share common ancestry from the root of the animal phylogeny. Further evaluation of the conservation of the human genes in the glycolysis pathway (obtained from Reactome database (Gillespie et al., 2022); R-HSA-70171) across six relevant environmental species was performed using the “Genes to Pathway - species conservation analysis tool” (G2P-SCAN; Rivetti et al., 2023).

## 3. Results and discussion

### 3.1. Conventional structure-based grouping hypothesis

**Fig. 1.**
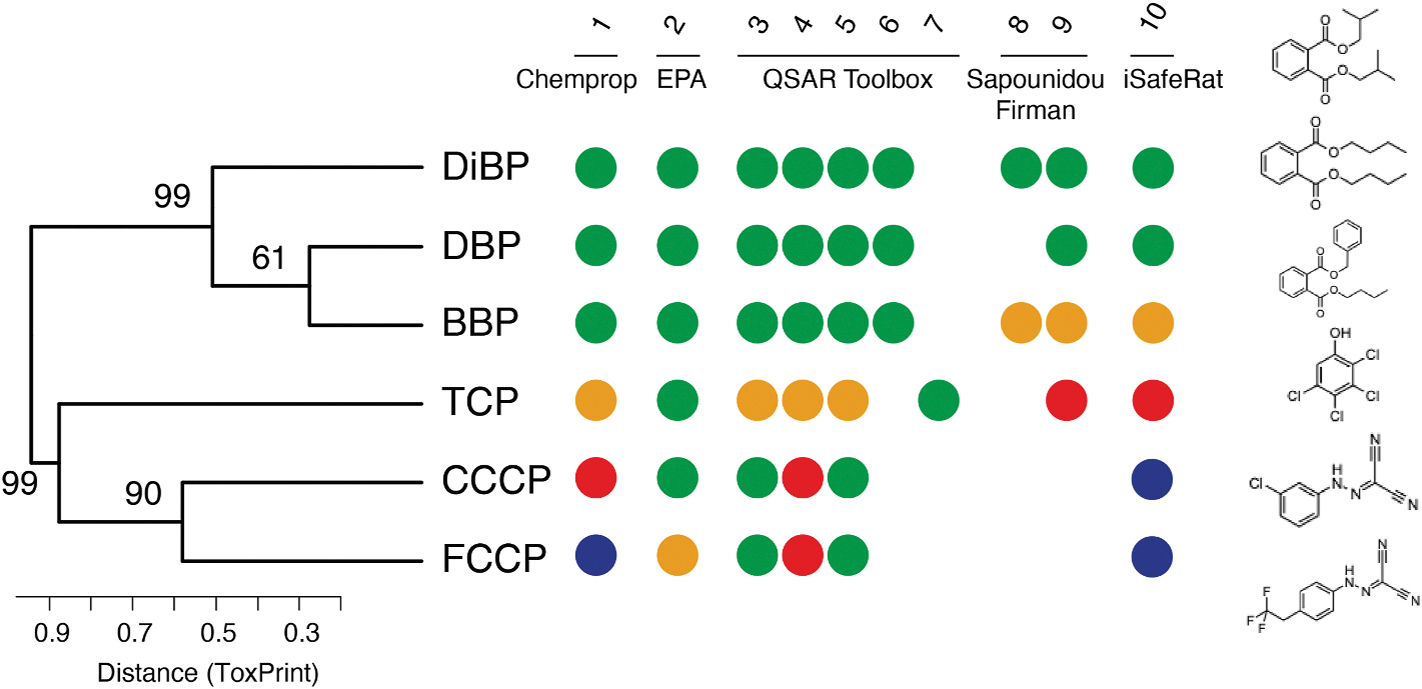
Structure-based similarity of selected test substances diisobutyl phthalate (DiBP), dibutyl phthalate (DBP), benzyl butyl phthalate (BBP), 2,3,4,5-tetrachlorophenol (TCP), carbonyl cyanide 3-chlorophenylhydrazone (CCCP) and carbonyl cyanide 4-(trifluoromethoxy)phenylhydrazone (FCCP) showing hierarchical clustering based on ToxPrint chemotypes. Selective inference (SI) bootstrap replicability confidence values are shown at each node. Summarised outputs of chemical classifiers are shown for each substance where colouring indicates the same classification within each tool. Classifier alerts for each substance obtained from (1) Chemprop for Russom, (2) US EPA Toxicity Estimation Software Tool, (3) OECD QSAR Toolbox for Verhaar acute aquatic toxicity, (4) Acute Aquatic Toxicity MOA by OASIS, (5) Estrogen Receptor Binding, (6) USEPA rtER Expert System, (7) mitochondrial toxicity (MITOTOX), (8–9) Sapounidou-Firman (Sapounidou et al., 2021; Firman et al., 2022) Non-Fish (8) and Fish (9) schemes, and (10) iSafeRat^®^ Mechanisms of toxic Action profiler. Full classifier outputs are presented in Supplemental Table S1.

Following RAAF guidance, which stipulates structural similarity between source and target substances is required for read-across, the structural similarity of the six test substances was assessed. HCA was applied to ToxPrint chemotypes (comprising 729 bits encoding the physicochemical properties of atoms, bonds and structural fragments; Yang et al., 2015) representing the test substances, and their structural similarities were visualised using a dendrogram (Fig. 1). The test substances clustered into two distinct groups, corresponding to the phthalates and uncouplers. The same grouping was observed when performing HCA with other types of structural fingerprints (see Supplemental section S1 and Fig. S1). Structural similarity among the three phthalates is comparatively high, reflecting their shared ortho-diester structure and relatively minor side-chain differences. The phthalates were also generally assigned the same classification using chemical profilers, except by the Sapounidou-Firman (Sapounidou et al., 2021; Firman et al., 2022) and MechoA profilers, for which BBP was assigned a different set of alerts and identified as SN2 reactivity, respectively. These different classifications for BBP compared to the other phthalates were attributed to the arene side chain group. In contrast, the uncouplers are a more structurally diverse group, and the profiler outputs were less consistent, with no approach assigning all three uncouplers to the same classification. Russom and MITOTOX (OECD QSAR Toolbox) assigned only TCP to oxidative phosphorylation uncouplers with no alerts for CCCP or FCCP. None of the structure-based profiling approaches assigned the uncouplers and phthalates into just two distinct groups; instead, there were up to four different classes assigned (e.g., Russom classifications; Table S1), highlighting the difficulty in associating the substances to their shared MoA and/or toxicity using chemical structure alone. These results formulated a conventional grouping hypothesis against which bioactivity profile-based grouping, using multi-omics data, was compared.

### 3.2. Magnitude of molecular responses to chemical exposure

**Fig. 2.**
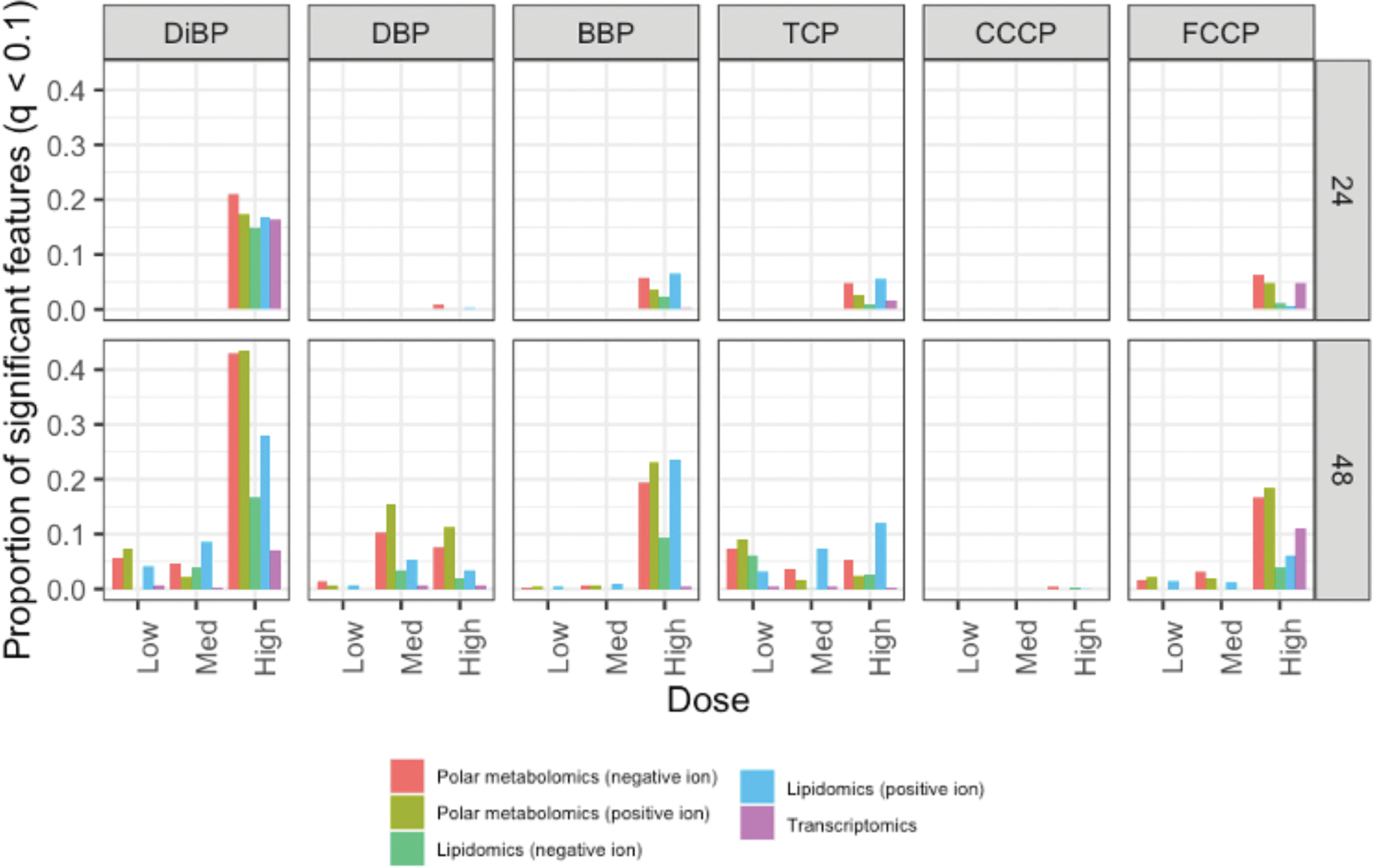
Proportion of the total number of features detected by each ‘omics assay that are differentially abundant (q < 0.1) between treated and control groups of juvenile Daphnia (5 d) collected following 24-h (top) and 48-h (bottom) exposures to the test substances diisobutyl phthalate (DiBP), dibutyl phthalate (DBP), benzyl butyl phthalate (BBP), 2,3,4,5-tetrachlorophenol (TCP), carbonyl cyanide 3-chlorophenylhydrazone (CCCP) and carbonyl cyanide 4-(trifluoromethoxy)phenylhydrazone (FCCP). Daphnia were exposed to each substance at low, medium and high doses, as indicated.

Before grouping the chemicals according to their ‘omics profiles, the magnitude of perturbation caused by each substance on a feature-by-feature basis was evaluated. This allows particularly weak or strong perturbations, which can lead to unreliable grouping, to be identified and removed (termed bioactivity thresholding). A total of 43,424 features (including 1944 genes, 22,244 polar metabolite features and 19,236 lipophilic compound features) were measured as part of the ‘omics profiles for the six substances. The numbers of significantly differentially abundant molecules (false discovery rate correction, *q* < 0.1) are presented in Fig. 2 and Table S5. As expected, the greatest molecular changes occurred at the high dose, and more features changed significantly after 48 h of exposure compared to 24 h. However, the variation in molecular perturbations was unexpectedly large, particularly considering that the doses were phenotypically anchored to *Daphnia* immobilisation. DiBP induced the largest molecular response with over 40 % of polar metabolite features (9625 out of 22,244; Table S5) significantly changing at the 48 h, high dose. Yet, minimal molecular changes were induced following exposure to CCCP with only 2 (at 24 h) and 21 (at 48 h) polar metabolite features significantly changing following high dose exposure, representing a minuscule portion of detected features, and no features changed significantly at low or medium doses. LC–MS/MS analysis of the exposure media revealed that at 0 h, the measured concentrations were within 30 % of nominal values for all substances apart from DiBP, which were 8 %, 44 % and 68 % lower than nominal concentrations for low, medium and high doses, respectively (Fig. S3), potentially due to adsorbing to the exposure vessels, volatilising or degrading. The test substance concentrations remained relatively consistent throughout the 48 h exposure period. The limited molecular responses induced by CCCP may have been caused by the reduced (i.e., 2x lower) doses for this substance (repeated, due to high mortality, at half the original doses, although the repeat exposures proceeded as expected with 14 % immobilisation observed in the high dose treatment after 48 h, which is close to the target value of 10 %; Table S3). Our workflow for bioactivity profile-based grouping assesses the similarities of the bioactivity profiles based on treatment effects versus controls (as t-statistics), and we have observed previously that non- and low-responding treatment groups are challenging to group reliably. Based on a bioactivity threshold requiring >1 % of total features detected (across all ‘omics assays) to be significantly perturbed, CCCP was identified for exclusion from the grouping analysis.

### 3.3. Bioactivity profile-based grouping using multi-omics data

**Fig. 3.**
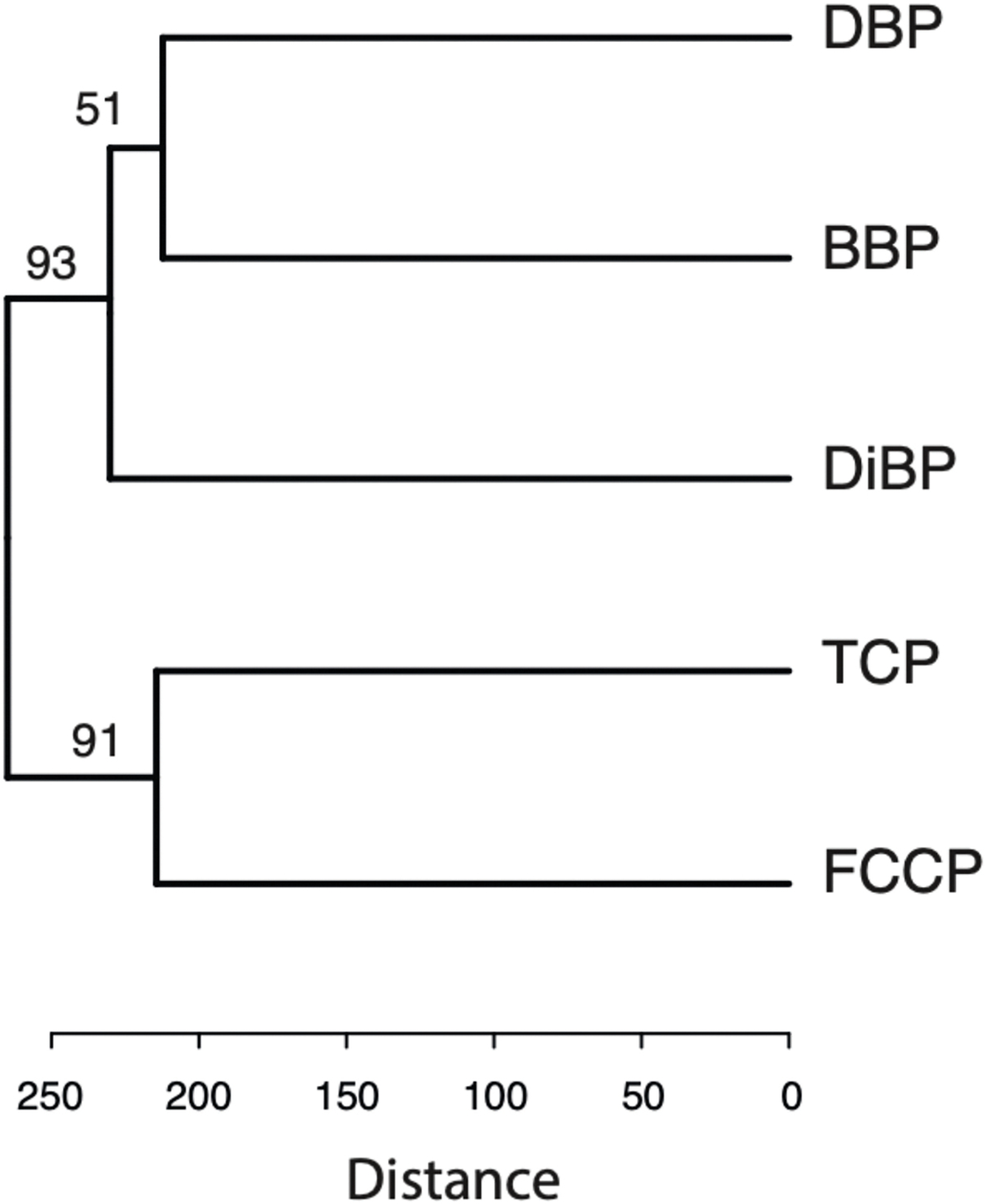
Bioactivity profile-based grouping presented as a dendrogram from a hierarchical cluster analysis of t-statistics derived from multi-omics data (transcriptomics and metabolomics) measured in samples of juvenile Daphnia (5 d) following 24-h and 48-h exposures to high doses of the test substances benzyl butyl phthalate (BBP), dibutyl phthalate (DBP), diisobutyl phthalate (DiBP), 2,3,4,5-tetrachlorophenol (TCP) and carbonyl cyanide 4-(trifluoromethoxy)phenylhydrazone (FCCP). Values at the top of the branches indicate % bootstrap replicability confidence p-values (using the selective inference method).

HCA was performed on the high dose (and control) multi-omics data to produce a bioactivity profile-based grouping of the five test substances exposed to *Daphnia*. Input data for this grouping analysis consisted of t-statistics comparing treated samples with their respective time-matched solvent controls for all 43,424 features (including genes, polar metabolites and lipophilic compounds) in the multi-omics profile. To enable the 24 h and 48 h data to be integrated, a ‘maximum perturbation’ approach was employed that selected the largest molecular perturbation (i.e., largest absolute t-statistic) from either the 24 h or 48 h time point, for all 43,424 features (Gruszczynska et al., 2024). In this manner, the interpretability of the grouping is increased by reducing the number of treatment groups in the dendrogram while retaining information about the temporal dynamics of the response. The results of the maximum perturbation bioactivity profile-based grouping are presented in Fig. 3. Statistical confidence in the groups was estimated by calculating replicability confidence *p*-values using the selective inference method. Here, branches with values > 90 % are interpreted as being strongly supported by the underlying ‘omics data. At this replicability confidence level, the five substances formed two distinct groups, separating the uncouplers of oxidative phosphorylation from the phthalates. This grouping agrees with the classical structural similarity comparisons (Fig. 1).

### 3.4. Attempted plausible toxicological interpretation

**Fig. 4.**
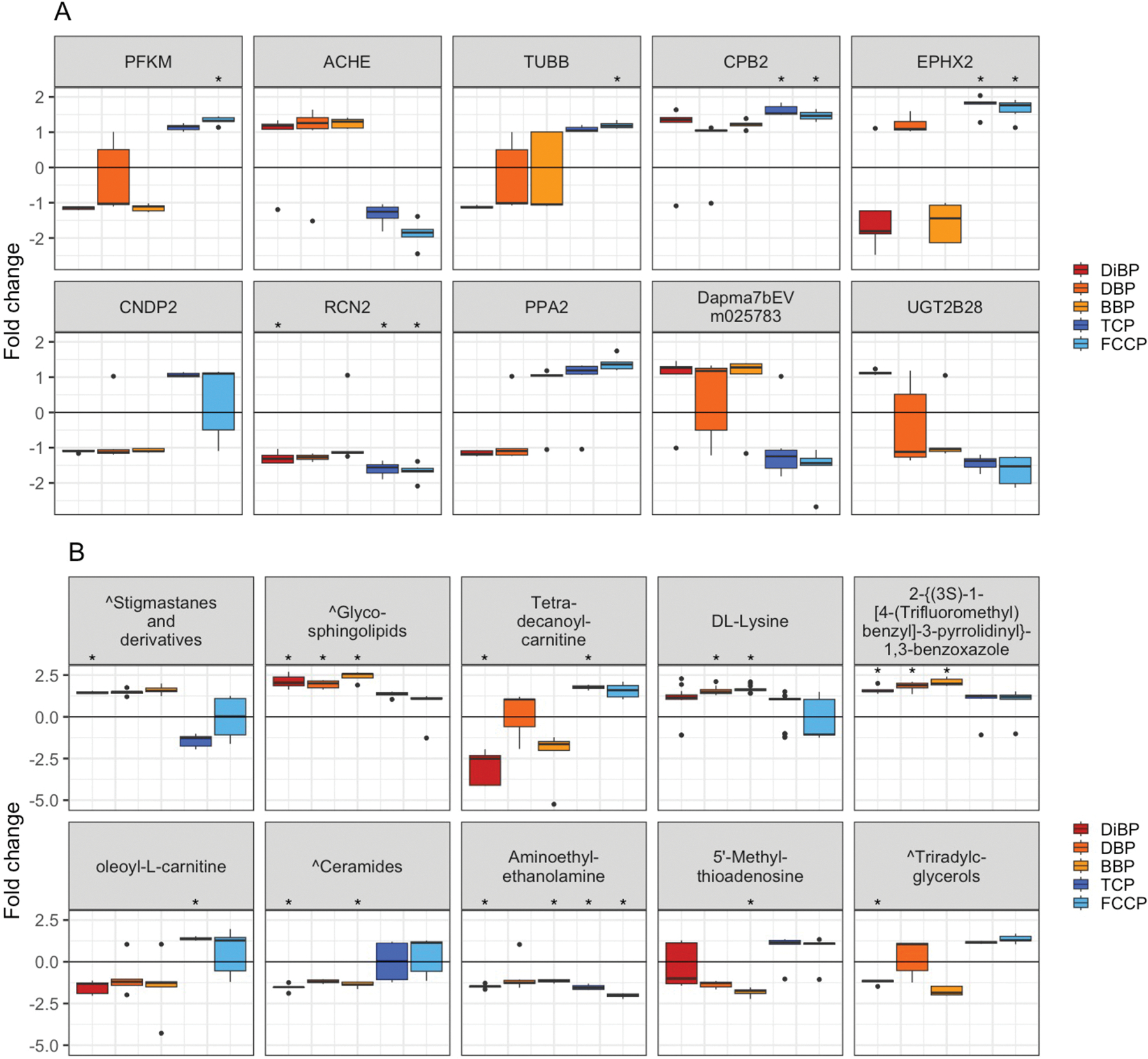
Fold-changes (−1/(log_2_ treatment −log_2_ control)) of top-ranked (A) annotated genes and (B) annotated metabolites and lipids derived from a partial least squares-discriminant analysis model following high dose treatments of test substances benzyl butyl phthalate (BBP), dibutyl phthalate (DBP), diisobutyl phthalate (DiBP), 2,3,4,5-tetrachlorophenol (TCP) and carbonyl cyanide 4-(trifluoromethoxy)phenylhydrazone (FCCP). Features are shown in descending (left to right) ranked order of their variable importance in projection scores. Asterisks above the box and whiskers indicate significant changes (q < 0.05). Panel headers indicate putative annotations showing either orthologous human gene symbols or metabolite/lipid names where (^) indicates features that could only be annotated to a subclass level. Gene symbols: PFKM - phosphofructokinase; ACHE - acetylcholinesterase; TUBB - tubulin beta class; CPB2 - carboxypeptidase B2; EPHX2 - epoxide hydrolase 2; CNDP2 - carnosine dipeptidase 2; RCN2 - reticulocalbin 2; PPA2 - pyrophosphatase 2; UGT2B28 - UDP glucuronosyltransferase family 2 member B28.

Although the agreement between structure-based and bioactivity profile-based grouping strengthens the grouping justification, further confidence can be achieved through a ‘plausible toxicological interpretation’ of the ‘omics responses underpinning each chemical category (OECD, 2017; Viant et al., 2024). Consequently, molecular features with fold-changes (treated samples versus controls) that differed between the uncoupler and phthalate groups (using dose and time point data consistent with the HCA; Fig. 3) were identified using PLS-DA supervised multivariate analysis, selecting 14,711 features with variable importance in projection (VIP) scores greater than one. Although this cut-off selected many features, including 464 annotated genes with human orthologs, relatively few metabolites (237) could be annotated and hence considered for toxicological interpretation (Supplemental section S4, Tables S6 and S7). This highlights a challenge of interpreting ‘omics responses using a reduced representative set of genes (here using a BioSpyder platform that measures 1988 *D. magna* genes mapped to human orthologs) instead of the entire transcriptome of human orthologs supplemented by the fraction of significantly changing metabolites that can be annotated. A second difficulty in attempting plausible toxicological interpretations of ‘omics datasets is the relative lack of knowledge of molecular signatures associated with chemical MoAs in *Daphnia*. Irrespective of these challenges, interpretation of the ‘omics data was attempted using a traditional approach of first prioritising the annotated gene and metabolite features (using rank order of their VIP scores from PLS–DA) and then searching the scientific literature for relevant prior knowledge. The fold-change values and statistical significance of the ten top-ranked genes, annotated metabolites, and unannotated metabolite features are presented in Fig. 4A and B and S4, respectively.

While phthalates fit the World Health Organization’s definition of endocrine disruptors for their effects in vertebrates, their MoA in invertebrates is largely unknown, and no evidence of endocrine disruption was observed here. However, we cannot exclude this MoA as the transcriptomics analysis used a reduced gene set that limited our ability to search for hypothesised effects on the endocrine system. Upon analysing the annotated metabolite dataset, the class and subclasses of metabolite features with the highest number of large VIP scores were ‘lipids and lipid-like molecules’ (Fig. S5), indicating differences in lipid metabolism between the phthalates and uncouplers. This is also supported by some top-ranked lipids in Fig. 4B (including a carnitine, ceramide and triadylcglycerol), which decreased significantly after exposure to at least one phthalate substance. These results appear consistent with changes in fatty acid metabolism and increased lipid accumulation previously reported in *Daphnia* in response to phthalate exposure (Seyoum and Pradhan, 2019), although the MoA remains unclear and care should be taken to not over-interpret the new observations given that only three phthalates were investigated.

The most striking differences between the two chemical categories are associated with disruption of energy metabolism, which is consistent with the MoA of uncouplers. In the highly ranked annotated metabolites (Fig. 4B), the levels of two putatively annotated acylcarnitines changed following exposure to the uncouplers, with significantly increased levels following TCP exposure. While associations between acylcarnitine and TCP or FCCP exposure have not been reported previously, accumulation of β-oxidation intermediates could indicate disruption of cellular energy metabolism through oxidative phosphorylation (Dambrova et al., 2022). The transcriptome data also indicated disruptions to energy metabolism, including effects on inorganic pyrophosphatase 2 (*PPA2*) expression levels (reduced following phthalate exposure and increased in response to uncouplers, though neither of these were statistically significant; Fig. 4A). *PPA2* plays a key role in phosphate metabolism, including oxidative phosphorylation and ATP generation (Phoon et al., 2020).

Furthermore, the highest-ranking gene was annotated as phosphofructokinase (*PFKM*), which was upregulated following high-dose FCCP and TCP exposures, although not statistically significant in the latter. As phosphofructokinase catalyses a rate-limiting step in glycolysis, this change in gene expression may be associated with the MoA of the uncouplers of oxidative phosphorylation. Five *D. magna* genes were identified as orthologues to human glycolysis enzymes (*GPI, PFKM, ALDO, PGK* and *ENO*) in the gene panel used here. The expression of these genes, the conservation of the human glycolysis pathway, and their functional relevance to the hypothesised MoA of the uncouplers in *Daphnia* are further explored in Supplemental section S5 (including Fig. S6 and Tables S8 and S9).

While some of the annotated genes, metabolites and lipids can be associated with the MoA of uncouplers and with previous observations of *Daphnia* exposed to phthalates, rigorous plausible toxicological interpretations that support the bioactivity profile-based grouping (Fig. 3) would require a greater number of annotated genes and metabolites in search of known MoA ‘signatures’ and toxicity related pathways that are enriched by significant features. For example, a plausible toxicological interpretation could include mapping the observed gene, metabolite and lipid changes to these signatures, assuming that these signatures are evolutionarily conserved between *Daphnia* and humans (Colbourne et al., 2022). In additional to reporting how extensively a given signature was detected (i.e., as a percentage of the number of features in the known signature), it would be valuable to indicate what proportion of the largest observed molecular changes can be explained by that (or those) MoA(s). Such future attempts at plausible toxicological interpretations in omics-based chemical grouping should benefit from greater inclusion of genes with mammalian orthologs to explore and evaluate their feasibility, for example using a genome-wide analysis of gene expression by RNA-Seq. Given the increasing knowledge of MoA signatures in human and mammalian systems, for example the MTox700+ metabolite biomarkers that are associated with multiple human health effects (Sostare et al., 2022), attempts to plausibly toxicologically interpret omics-based grouping results will become more achievable.

## 4. Conclusions

Grouping the six test substances using chemical profilers (based on structural alerts) had limited success in assigning them to the two anticipated groups based on prior knowledge of their MoAs. Substances were assigned to up to four groups depending on which profiler was applied. Grouping based on structural similarity yielded results that were more consistent with expectations, placing the phthalates and uncouplers into distinct categories. Statistical analysis of the *Daphnia* multi-omics data revealed considerable differences between the number of significantly perturbed molecular features per substance, indicating that improvements in experimental design are needed to generate ‘omics datasets that generate a more consistent level of molecular perturbations across the treatment groups. Despite this variation in the number of perturbed features, the multi-omics bioactivity profile-based grouping workflow separated the three phthalates and two uncouplers into distinct categories with high replicability confidence scores. Applying robust statistical procedures to calculate the bioactivity similarities between a series of molecular responses, in addition to defining how such procedures and their results should be reported, is important for building confidence in the reliability and transparency of bioactivity-based grouping using ‘omics data. Constrained in part by the lack of knowledge of MoA signatures in *Daphnia*, our attempted plausible toxicological interpretation was limited in scope. However, some metabolite and gene expression changes could be associated with anticipated changes in cellular energy metabolism that result from exposure to uncouplers of oxidative phosphorylation. Assuming the availability of more established MoA signatures for the biological test system being measured, for example generated by investigating a series of “anchor chemicals” with known MoAs, future grouping studies should investigate more robust and transparent strategies for the plausible toxicological interpretations of ‘omics datasets.

## Supplementary Material

Supplement1

## Data Availability

Data will be made available on request.

## Appendix A. Supplementary data

Supplementary data to this article can be found online at https://doi.org/10.1016/j.yrtph.2025.105894.
