# Supplementary material for: Substantiating chemical groups for read-across using molecular response profiles
Source: Regul Toxicol Pharmacol. Author manuscript; Available in PMC 2026 Jun 26. (PMC12598511; doi:10.1016/j.yrtph.2025.105894)
Supplement: Supplement1 [file NIHMS2121688-supplement-Supplement1.docx]

### Supplemental materials

##### Section S1 - Additional structural fingerprint-based grouping

In addition to grouping the test substances based on ToxPrint chemotypes, four other structural fingerprints (MACCS (Durant *et al. 2002* ), Morgan (Rogers *et al.* 2010), PubChem ([ftp://ncbi.nlm.nih.gov/pubchem/specifications/pubchem_fingerprints.txt](https://ncbi.nlm.nih.gov/pubchem/specifications/pubchem_fingerprints.txt)) and RDkit (Daylight-like) (https://www.rdkit.org) calculated using RDKit (https://www.rdkit.org) and PyFingerprint (Hongchao *et al.* 2023)) were also used as the basis for grouping using the *pvclust* package (version 2.2-0; Suzuki & Shimodaira 2006) with method.dist = "binary", method.hclust = "ward.D2", and n = 10, 000 bootstrap pseudo-replications. Clusters where the *pvclust* calculated selective inference (SI) value was ≥ 0.90 were considered strongly supported by the data. As with the ToxPrint chemotype analysis detailed in the main paper, all of the additional structural fingerprint clustering supported a clear separation of the phthalates and uncouplers (Figure S1). The only inconsistency between the clustered groups using the five different fingerprints is for the internal grouping structure of the phthalates. Using the Topological RDKit and PubChem fingerprints, DBP and DiBP are marginally more similar to one another than BBP. However, using the ToxPrint chemotypes, MACCS, and Morgan fingerprints, DBP and BBP are considered more similar within the internal grouping structure of phthalates.


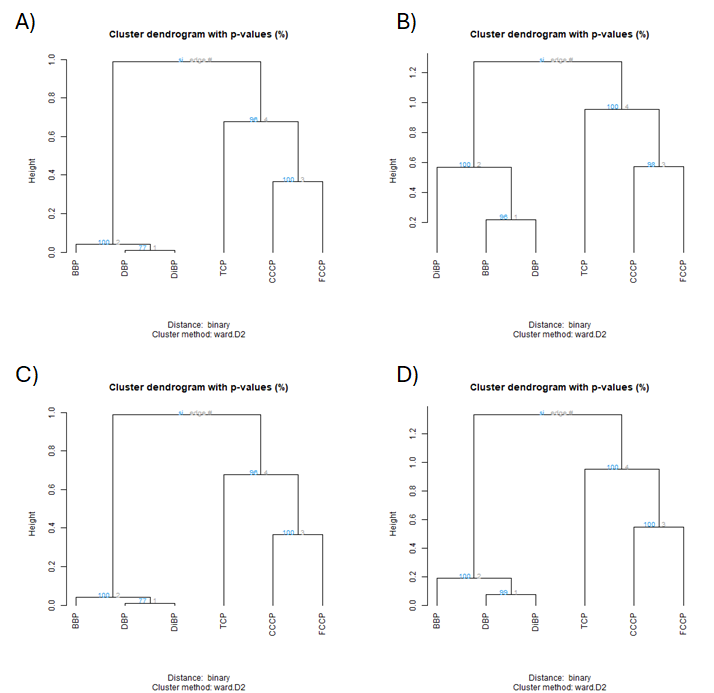


Figure S1. Structural similarity of test substances using hierarchical cluster analysis of the binary distance calculated between each pair of structural fingerprints. A) MACCS fingerprints. B) Morgan fingerprints. C) Topological RDKit fingerprints. D) PubChem fingerprints. SI = selective inference.

Table S1. Summary of outputs from the classification of test substances diisobutyl phthalate (DiBP), dibutyl phthalate (DBP), benzyl butyl phthalate (BBP), 2, 3, 4, 5-tetrachlorophenol (TCP), carbonyl cyanide 3- chlorophenylhydrazone (CCCP) and carbonyl cyanide 4-(trifluoromethoxy)phenylhydrazone (FCCP) using Russom, OECD QSAR Toolbox and LJMU/SEAC Fish and Non-fish profiler tools.

|  |  | **Phthalates** | | | **Oxidative uncouplers** | | |
| --- | --- | --- | --- | --- | --- | --- | --- |
|  |  | **DiBP** | **DBP** | **BBP** | **TCP** | **CCCP** | **FCCP** |
| **Chemprop for Russom** | **Classification** | Narcosis III: Ester narcosis | Narcosis III: Ester narcosis | Narcosis III: Ester narcosis | Oxidative phosphorylation uncouplers | Narcosis I | Reactive electrophiles/ proelectrophiles |
|  | **Applicability Domain** | 0 (out of domain) | 3 (in domain) | 2 (borderline in domain) | 3 (in domain) | 0 (out of domain) | 0 (out of domain) |
| **US EPA TEST - Predicted MoA** | | Narcosis | Narcosis | Narcosis | Narcosis | Narcosis | Reactivity |
| **OECD QSAR Toolbox** | **Acute aquatic toxicity classification by Verhaar (Modified)** | Class 3 (unspecific reactivity) | Class 3 (unspecific reactivity) | Class 3 (unspecific reactivity) | Class 4 (Compounds and groups of compounds acting by a specific mechanism) | Class 3 (unspecific reactivity) | Class 3 (unspecific reactivity) |
|  | **Acute aquatic toxicity MoA by OASIS** | Esters | Esters | Esters | Phenols and Anilines | Reactive unspecified | Reactive unspecified |
|  | **Protein binding by OASIS** | No alert found | No alert found | No alert found | No alert found | No alert found | No alert found |
|  | **DNA binding by OASIS** | No alert found | No alert found | No alert found | No alert found | No alert found | No alert found |
|  | **TB Estrogen Receptor Binding** | Non binder, without OH or NH2 group | Non binder, without OH or NH2 group | Non binder, without OH or NH2 group | Strong binder, OH group | Non binder, without OH or NH2 group | Non binder, without OH or NH2 group |
|  | **rtER Expert System - USEPA** | Phthalates | Phthalates | Phthalates | No alert found | No alert found | No alert found |
|  | **Uncouplers (MITOTOX)** | Undefined | Undefined | Undefined | High concern for uncoupling OXPHOS | Undefined | Undefined |
| **iSafeRat® Mechanisms of toxic Action profiler (MechoA)** | | MechoA 2.1 & mC2.2: Digestive hydrolysis of one ester, monoester product induces PPARalpha (precursor of liver cancer development) in chronic timeframe and is an endocrine disruptor for mammals. | MechoA 2.1 & mC2.2: Digestive hydrolysis of one ester, monoester product induces PPARalpha (precursor of liver cancer development) in chronic timeframe and is an endocrine disruptor for mammals. | MechoA 3.1 & m4.1: SN2 reactivity leading to proteins and DNA adducts for all species & conjugation with GSH (detoxification) for mammals | MechoA 5.1: Oxidative Phosphorylation Uncoupling by inner mitochondrial membrane pH gradient suppression (or other vesicles pH gradient) for all species. | MechoA 3.1 & 4.3: Hard electrophile reactivity and metabolism into electrophile for all species. | MechoA 3.1 & 4.3: Hard electrophile reactivity and metabolism into electrophile for all species. |
| **Sapounidou Non-fish** | **Alert name** | Not propargylic or allylic containing only C, H, O and Halogen |  | Arenes-(MA) |  |  |  |
|  | **MIE** | Accumulation in membrane-based phospholipids |  | Alkylation |  |  |  |
|  | **Reference(s)** | Verhaar et al 1992 |  | AOP-Wiki, 2018; Di Guilo and Hinton, 2008; Enoch and Cronin, 2014 |  |  |  |
|  | **Target** | cellular membranes |  | Protein / DNA |  |  |  |
|  | **Domain** | 1. Narcosis - Non-specific effects |  | 2. Reactive / chemistry based |  |  |  |
|  | **Example compounds** | Not propargylic or allylic containing only C, H, O and Halogen |  | Arenes-(MA) |  |  |  |
|  | **Taxanomical applicability** | Fish, ciliates, bacteria, algae, invertebrates |  | Eukaryota |  |  |  |
|  | **Description of mechanism 1** | Non-specific accumulation within biological membranes – observed as being slightly more toxic than predicted by baseline toxicity equation (i.e., Veith and Broderius, 1987) |  | Transformation into a reactive product through metabolic or oxidative processes |  |  |  |
|  | **Experimental MOA** | N/A |  | Narcosis |  |  |  |
| **Sapounidou Fish** | **Domain** | 1. Non-specific effects | 1. Non-specific effects | 2. Reactive / chemistry based | 3. Specific / biologically based | no information available | no information available |
|  | **Mechanistic group** | 1.1 Narcosis | 1.1 Narcosis | 2.1 Electrophilic | 3.5 Hormonal function disruption | no information available | no information available |
|  | **Mechanism** | 1.1.2 Polar | 1.1.2 Polar | 2.1.1 Soft | 3.5.1 Binding to nuclear receptors (oestrogen, androgen, thyroid receptors) | no information available | no information available |
|  | **Alert name** | Not propargylic or allylic containing only C, H, O and Halogen | Not propargylic or allylic containing only C, H, O and Halogen | Arenes-(MA) | Hexachlorobenzene | no information available | no information available |
|  | **MIE** | Accumulation in membrane-based phospholipids | Accumulation in membrane-based phospholipids | Michael addition | Covalent binding leading to activation | no information available | no information available |
|  | **Reference(s)** | Verhaar et al 1992 |  | Di Guilo and Hinton, 2008; Enoch and Cronin, 2010; Enoch et al, 2011 |  |  |  |
|  | **Target** | cellular membranes |  | Protein / DNA |  |  |  |
|  | **Example compounds** | Not propargylic or allylic containing only C, H, O and Halogen |  | Arenes-(MA) |  |  |  |
|  | **Taxonomical applicability** | Fish, ciliates, bacteria, algae, invertebrates |  | Teleost Fish, Tetrahymena pyriformis, Daphnia magna |  |  |  |
|  | **Description of mechanism 1** | Non-specific accumulation within biological membranes – observed as being slightly more toxic than predicted by baseline toxicity equation (i.e. Veith and Broderius, 1987) |  | Covalent interaction of soft electrophiles with biological membranes causing permanent disruption |  |  |  |


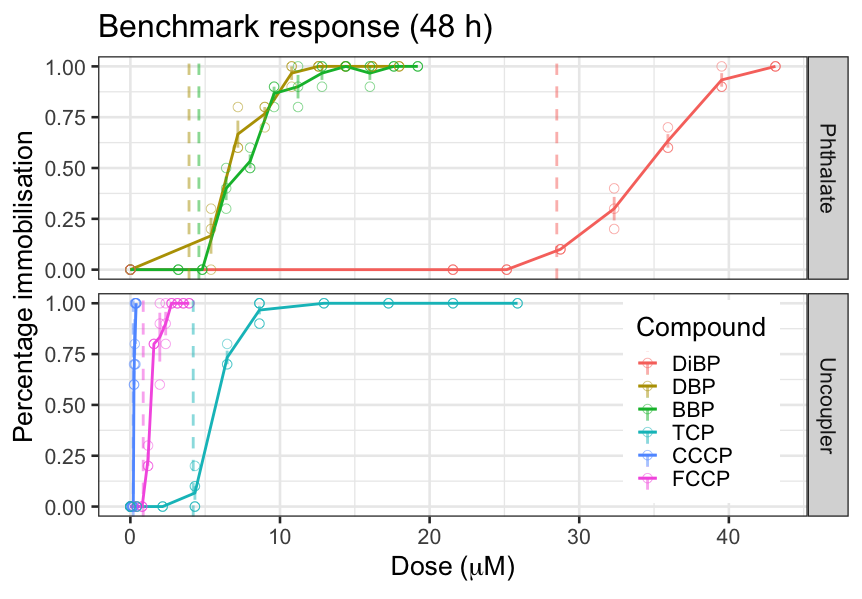


Figure S2. Mean proportion of Daphnia magna immobilised (solid line) following 48 h exposure of neonates (<24 h old) to test substances diisobutyl phthalate (DiBP), dibutyl phthalate (DBP), benzyl butyl phthalate (BBP), 2, 3, 4, 5-tetrachlorophenol (TCP), carbonyl cyanide 3- chlorophenylhydrazone (CCCP) and carbonyl cyanide 4-(trifluoromethoxy)phenylhydrazone (FCCP). Open circular points indicate individual experimental replicates (n=3), error bars indicate standard error and vertical dashed lines indicate the lower estimate of the benchmark dose for 10% immobilisation.

Table S2. Summary of benchmark dose analysis completed using the PROAST webtool to derive the lower (BMDL) and upper (BMDU) estimates of 10% immobilisation of Daphnia magna following 48 h exposure to test substances diisobutyl phthalate (DiBP), dibutyl phthalate (DBP), benzyl butyl phthalate (BBP), 2, 3, 4, 5-tetrachlorophenol (TCP), carbonyl cyanide 3- chlorophenylhydrazone (CCCP) and carbonyl cyanide 4-(trifluoromethoxy)phenylhydrazone (FCCP).

| **Substance** | **BMDL (µM)** | **BMDU (µM)** |
| --- | --- | --- |
| DiBP | 28.5 | 31.0 |
| DBP | 3.92 | 5.32 |
| BBP | 4.58 | 5.73 |
| TCP | 4.2 | 5.00 |
| CCCP | 0.189 | 0.218 |
| FCCP | 0.858 | 1.10 |

Table S3. Summary of dose groups applied during exposure of 5-d Daphnia magna to selected test substances diisobutyl phthalate (DiBP), dibutyl phthalate (DBP), benzyl butyl phthalate (BBP), 2, 3, 4, 5-tetrachlorophenol (TCP), carbonyl cyanide 3- chlorophenylhydrazone (CCCP) and carbonyl cyanide 4-(trifluoromethoxy)phenylhydrazone (FCCP) for multi-omics analysis. The lower estimate of benchmark dose for 10% (48 h) immobilisation (BMDL) was used as the high exposure dose with the medium dose group being one third BMDL and low dose group being one ninth BMDL. Moderate toxicity was observed in the “Original” high dose CCCP treated samples with a greater than anticipated (>10%) mortality. Exposures with CCCP were therefore repeated at a slightly reduced dose range of one half BMDL for high dose and one-third and one-ninth this value for medium and low dose, respectively.

| **Substance** | **Dose group** | **Concentration (µM)** |
| --- | --- | --- |
| DiBP | Low | 3.16 |
|  | Medium | 9.48 |
|  | High | 28.45 |
| DBP | Low | 0.43 |
|  | Medium | 1.3 |
|  | High | 3.92 |
| BBP | Low | 0.51 |
|  | Medium | 1.53 |
|  | High | 4.58 |
| TCP | Low | 0.47 |
|  | Medium | 1.4 |
|  | High | 4.2 |
| CCCP (Original) | Low | 0.02 |
|  | Medium | 0.06 |
|  | High | 0.19 |
| CCCP (Repeat) | Low | 0.01 |
|  | Medium | 0.03 |
|  | High | 0.09 |
| FCCP | Low | 0.1 |
|  | Medium | 0.29 |
|  | High | 0.86 |

##### Section S2 - Extended description of analytical methods to determine exposure concentrations

The concentrations of test substances were analysed in media (1 mL) from exposed *Daphnia* experiments collected at 0, 24 and 48 h from 3 separate exposure vessels (n = 3) during the course of exposures for multi-omics sampling. Samples were analysed using liquid chromatography coupled to a triple quadrupole mass analyser (LC-MS/MS) with an electrospray ion source (Xevo TQD, Waters, Milford, USA). To optimize the method to have high sensitivity, flow injection analysis of individual standard solutions (1 mg L^-1^) was performed at a cone voltage varying from 10 to 130 V to select the voltage at which the molecular ion was present with the highest intensity and then the collision energy was optimised in a range from 10 to 50 eV to select the most intense and selective fragment ions. Individual test substances from each *Daphnia* exposure experiment were analysed using direct sample injection (10 µL). A BEH C18 analytical column (100 mm x 2.1 mm, particle size 1.7 µm) (Acquity, Waters, Milford, USA) was deployed using (A) methanol:acetonitrile (25–75%) and (B) water with 20 mM ammonium acetate and 1% acetic acid as mobile phase. Gradient elution started at 60% A and 40% B, increasing to 100% A in 5 min (5 min hold time) and back to initial conditions in 1 min, at a flow rate of 200 µLmin^-1^. Acquisition was performed in selected reaction monitoring (SRM) mode using two transitions from the precursor ion to the product ion to identify each compound (Table S4). Identification criteria included the retention time and two transitions, one used for quantification and the other for confirmation. Before injection, samples were spiked with 100 ng of the labelled surrogate standards deuterated diisopentyl phthalate (DiPP-d_4_) and ^13^C perfluorooctane sulfonate (MPFOS) for phthalate and uncouplers analysis, respectively. Phthalates were measured under positive electrospray ionisation (ESI+), and uncouplers with negative electrospray (ESI-). The system and data management were processed using MassLynx v4.1 software package. The limits of detection (LOD) were calculated as 3 times the signal to noise ratio of the lowest concentration of each analyte in the calibration curve (from 0.001 to 0.75 mg L^-1^; Table S4). DBP was the only compound that had a blank contribution (0.14±0.04 mg L^-1^) which was subtracted from the calculated concentration.

Table S4. LC–MS/MS conditions and quality parameters for the analysis of test substances, indicating LC retention time (RT), cone voltage, quantification (Q) and confirmation (q) transitions used, collision energy (in brackets), limits of detection (LOD), calibration curve concentration ranges (coefficient of determination R2 was 0.999 for all substances), and percentage recovery with standard deviation (%R±SD).

| **Substance** | **RT** | **Cone** | **Q** | **q** | **LOD  (mgL^-1^)** | **Calibration** | **% R±SD** |
| --- | --- | --- | --- | --- | --- | --- | --- |
|  | **(min)** | **(V)** | **transition** | **transition** |  | **(mgL^-1^)** |  |
| DiBP | 6.43 | 25 | 279 > 205 (10) | 279 > 149 (20) | 0.0003 | 0.025-0.75 | 72±4 |
| DBP | 6.56 | 15 | 279 > 205 (20) | 279 > 121 (20) | 0.0002 | 0.025-0.75 | 121±3 |
| BBP | 6.51 | 65 | 313 > 149 (20) | 313 > 91 (20) | 0.00005 | 0.025-0.75 | 88±4 |
| TCP | 2.64 | 26 | 231 > 231 (20) | - | 0.005 | 0.02-0.70 | 115±4 |
| CCCP | 1.55 | 25 | 203 > 203 (25) | 203 > 151 (20) | 0.0002 | 0.001-0.05 | 103±11 |
| FCCP | 2.01 | 50 | 253 > 253 (20) | 253 > 132 (20) | 0.006 | 0.01-0.25 | 68±2 |


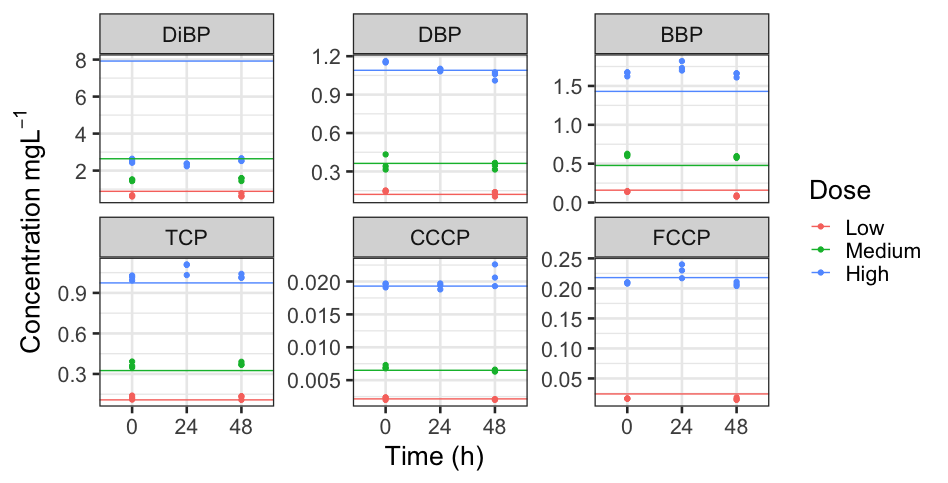


Figure S3. Concentrations of test substances benzyl butyl phthalate (BBP), dibutyl phthalate (DBP), diisobutyl phthalate (DiBP), 2, 3, 4, 5-tetrachlorophenol (TCP), carbonyl cyanide 3-chlorophenylhydrazone (CCCP) and carbonyl cyanide 4-(trifluoromethoxy)phenylhydrazone (FCCP; not detected in the medium dose samples) at three time points throughout the D. magna exposure studies. Data points represent individual sample measurements (n=3) obtained by LC–MS/MS analysis of exposure media, and the horizontal lines indicate nominal exposure concentrations.

##### Section S3 - Extended description of methods for metabolomics data acquisition and processing

Metabolites were extracted using a biphasic extraction method modified from Southam *et al.* (2021). Following the removal of the 403 µL homogenate into a 1.8 mL glass vial, the homogenisation tube was washed with 100 µL methanol/water (71.4/28.6, v/v), which was then added to the glass vial containing 403 µL homogenate. Chloroform (HPLC plus grade, Sigma) and water (LC-MS grade, Merck) were added to the glass vial to create a solvent ratio of 2:2:1.8 methanol:chloroform:water, and the sample was vortexed (15 s), incubated on ice (10 min), and centrifuged (2, 500-*g*, 4°C, 10 min). The sample was set at room temp (20°C, 5 min) to allow completion of phase partitioning, then the top layer (containing polar metabolites) was removed into a clean tube and dried in a SpeedVac concentrator (Thermo Scientific), and the bottom layer (containing lipophilic compounds) removed into a separate tube and dried under a stream of nitrogen gas (Techne FSC400D, Thermo Scientific). Extract blank samples were prepared in the same way as biological samples in the absence of biological material. Dried extracts were stored at -80°C until mass spectrometric analysis.

Directly prior to mass spectrometric analysis, dried biological extracts and extract blank samples were resuspended in either: (a) 30 µL 3:1 isopropanol:water [non-polar extract], or (b) 30 µL 1.5:1.5:1 methanol:acetonitrile:water [polar extract]. Following sample vortexing (15 s) and centrifugation (20, 000-*g*, 15 min, 4°C), 14 µL of the supernatant was loaded into chromatography vials with low recovery silanised inserts and set at 4°C until analysis. Intrastudy quality control (QC) samples were prepared by pooling 8 µL supernatant from each biological sample, vortexing the pool (30 s) and loading 14 µL into HPLC vials with low recovery silanized inserts (9 mm autosampler inserts, Thermo Scientific). All samples were set at a temperature of 4°C and maintained at 4°C during the analytical batch. Samples were analysed using two ultra high performance liquid chromatography mass spectrometry (UHPLC-MS) metabolomics methods (HILIC and C18 Lipids assays) using a Dionex UltiMate 3000 Rapid Separation LC system coupled with a heated electrospray Q Exactive Focus mass spectrometer (Thermo Scientific).

Polar extracts were analysed by a HILIC assay using an Accucore 150 Amide HILIC column (100×2.1 mm, 2.6 µm, Thermo Scientific). For positive ion HILIC analysis, mobile phase A was 10 mM ammonium formate dissolved in acetonitrile/water/formic acid (95:4.9:0.1 (v/v) and mobile phase B was 10 mM ammonium formate dissolved in acetonitrile/water/formic acid (50/49.9/0.1 (v/v)). For negative ion HILIC analysis, mobile phase A was 10 mM ammonium acetate dissolved in acetonitrile/water/acetic acid (95:4.9:0.1 (v/v) and mobile phase B was 10 mM ammonium acetate dissolved in acetonitrile/water/acetic acid (50/49.9/0.1 (v/v)). The gradient elution applied was t=0.0, 1% B; t=1.0, 1% B; t=3.0, 15% B; t=6.0, 50% B; t=9.0, 95% B; t=10.0, 95% B; t=10.5, 1% B; t=14.0, 1% B. All changes were linear (curve = 5) and the flow rate was 0.50 mL/min. Column temperature was 35 °C and injection volume was 2 μL. Data were acquired in positive and negative ionisation mode separately (70-1050 *m/z*) with a mass resolution of 70, 000 (FWHM, *m/z* 200). Ion source parameters: sheath gas = 55 arbitrary units, aux gas = 14 arbitrary units, sweep gas = 4 arbitrary units, spray voltage = +3.2kV (positive ion) / -2.7kV (negative ion), capillary temperature = 380°C, aux gas heater temperature = 440°C. Thermo ExactiveTune (2.8 SP1, build 2806) software controlled the instruments and data acquisition. All data were acquired in profile mode.

Non-polar extracts were analysed by a C18 Lipids assay using a Hypersil GOLD C18 column (100×2.1 mm, 1.9 μm; Thermo Scientific). Mobile phase A was 10 mM ammonium formate dissolved in acetonitrile/water/formic acid (60:39.9:0.1 (v/v) and mobile phase B was 10 mM ammonium formate dissolved in isopropanol/acetonitrile/water/formic acid (85.5/9.5/4.9/0.1 (v/v)). The gradient elution applied was t=0.0, 20% B; t=0.5, 20% B, t=8.5, 100% B; t=9.5, 100% B; t=11.5, 20% B; t=14.0, 20% B. All changes were linear (curve = 5) and the flow rate was 0.40 mL/min. Column temperature was 55°C and injection volume was 2 μL. Data were acquired in positive and negative ionisation mode separately (150–2000 *m/z*) with a mass resolution of 70, 000 (FWHM, *m/z* 200). Ion source parameters: sheath gas = 48 arbitrary units, aux gas = 15 arbitrary units, sweep gas = 0 arbitrary units, spray voltage = +3.2 kV (positive ion) / -2.7 kV (negative ion), capillary temperature = 380°C, aux gas heater temperature = 450°C. Thermo ExactiveTune (2.8 SP1, build 2806) software controlled the instruments and data acquisition. All data were acquired in profile mode.

For both the HILIC and C18 Lipids assays, intrastudy QC samples were analysed as the fourteen of the first fifteen injections (to condition the analytical platform) and then every sixth injection was a QC sample with two QC samples injected at the end of the analytical batch. An extract blank sample was analysed as the fifth injection and then the last injection of each batch. All data were collected as MS1 data in profile mode with the exception of several intrastudy QC sample injections where MS/MS data were collected in the “Discovery mode” setting over different precursor *m/z* ranges (HILIC: 70–140 *m/z*, 115–185 *m/z*, 150–220 *m/z*, 200–310 *m/z*, 300–510 *m/z*, 500–1050 *m/z*; lipids: 150–300 *m/z*, 290–365 *m/z*, 355–460 *m/z*, 450–860 *m/z*; 850–1010 *m/z*; 1000–2000 *m/z*) using stepped normalised collision energies (NCE) of 20, 40, 130. For each assay, the 6 x MS/MS *m/z* ranges were acquired twice (1) using the multiple metabolic biomarkers from the new MTox700+ panel (Sostare *et al*., 2022) as an inclusion list and (2) using the multiple metabolic biomarkers from the new MTox700+ panel (Sostare *et al*., 2022) as an exclusion list to increase fragmentation of unknowns.

Changes in the levels of several thousand polar metabolic and lipid features were determined, and features were identified using UHPLC-MS/MS and an in-house metabolite library (Lloyd, Jankevics and Weber, 2020). Additional metabolite annotations were obtained using the Galaxy Deep Metabolome Annotation (DMA) computational workflow, where annotations are combined and ranked from SIRIUS CSI:FingerID (Dührkop *et al.*, 2019), MetFrag (Ruttkies *et al.*, 2016) and spectral matching to mass spectrometry fragmentation library using msPurity. The annotations from the Galaxy DMA workflow were then filtered (spectral match >0.7 or a MetFrag score >0.98). If a feature could not be distilled to a single metabolite annotation at rank of 1, then the annotation was removed from further analysis.

Table S5. Number of features detected by each ‘omics assay that are differentially abundant (q<0.1) between treated and control groups of juvenile D. magna (5 d) collected following 24 h and 48 h exposures to the test substances benzyl butyl phthalate (BBP), dibutyl phthalate (DBP), diisobutyl phthalate (DiBP), 2, 3, 4, 5-tetrachlorophenol (TCP), carbonyl cyanide 3-chlorophenylhydrazone (CCCP) and carbonyl cyanide 4-(trifluoromethoxy)phenylhydrazone (FCCP). D. magna were exposed to each substance at low, medium and high doses, as indicated. ‘Max’ column indicates the number of features when the largest absolute t-statistic from either 24 h or 48 h high dose exposures are selected as presented in the ‘maximum perturbation’ multi-omics grouping approach (Figure 3).

|  |  |  | **48 h  exposure** | | | **24 h exposure** |  |
| --- | --- | --- | --- | --- | --- | --- | --- |
| **Substance** | **Assay** | **Total** | **Low** | **Medium** | **High** | **High** | **Max** |
| BBP | Transcriptome | 1944 | 0 | 0 | 7 | 3 | 10 |
|  | Polar metabolites (-) | 6446 | 11 | 40 | 1245 | 365 | 1450 |
|  | Polar metabolites (+) | 15798 | 58 | 103 | 3645 | 573 | 3964 |
|  | Lipophilic compounds (-) | 6974 | 0 | 0 | 652 | 168 | 775 |
|  | Lipophilic compounds (+) | 12262 | 66 | 105 | 2884 | 799 | 3254 |
|  |  | 43424 | 135 | 248 | 8433 | 1908 | 9453 |
| DBP | Transcriptome | 1944 | 0 | 14 | 13 | 0 | 13 |
|  | Polar metabolites (-) | 6446 | 95 | 667 | 480 | 58 | 501 |
|  | Polar metabolites (+) | 15798 | 118 | 2457 | 1775 | 3 | 1763 |
|  | Lipophilic compounds (-) | 6974 | 0 | 242 | 129 | 1 | 130 |
|  | Lipophilic compounds (+) | 12262 | 92 | 644 | 418 | 22 | 429 |
|  |  | 43424 | 305 | 4024 | 2815 | 84 | 2836 |
| DiBP | Transcriptome | 1944 | 12 | 4 | 136 | 317 | 392 |
|  | Polar metabolites (-) | 6446 | 367 | 297 | 2770 | 1348 | 3229 |
|  | Polar metabolites (+) | 15798 | 1143 | 348 | 6855 | 2736 | 7773 |
|  | Lipophilic compounds (-) | 6974 | 0 | 272 | 1163 | 1032 | 1786 |
|  | Lipophilic compounds (+) | 12262 | 512 | 1062 | 3437 | 2065 | 4490 |
|  |  | 43424 | 2034 | 1983 | 14361 | 7498 | 17670 |
| CCCP | Transcriptome | 1944 | 0 | 0 | 0 | 0 | 0 |
|  | Polar metabolites (-) | 6446 | 0 | 0 | 21 | 2 | 23 |
|  | Polar metabolites (+) | 15798 | 0 | 0 | 0 | 0 | 0 |
|  | Lipophilic compounds (-) | 6974 | 0 | 0 | 4 | 0 | 4 |
|  | Lipophilic compounds (+) | 12262 | 0 | 0 | 6 | 1 | 7 |
|  |  | 43424 | 0 | 0 | 31 | 3 | 34 |
| FCCP | Transcriptome | 1944 | 0 | 0 | 215 | 95 | 266 |
|  | Polar metabolites (-) | 6446 | 109 | 194 | 1069 | 396 | 1296 |
|  | Polar metabolites (+) | 15798 | 346 | 287 | 2908 | 743 | 3311 |
|  | Lipophilic compounds (-) | 6974 | 0 | 0 | 270 | 82 | 311 |
|  | Lipophilic compounds (+) | 12262 | 176 | 128 | 739 | 64 | 772 |
|  |  | 43424 | 631 | 609 | 5201 | 1380 | 5956 |
| TCP | Transcriptome | 1944 | 7 | 7 | 3 | 32 | 35 |
|  | Polar metabolites (-) | 6446 | 474 | 236 | 338 | 314 | 583 |
|  | Polar metabolites (+) | 15798 | 1420 | 250 | 388 | 407 | 747 |
|  | Lipophilic compounds (-) | 6974 | 418 | 3 | 185 | 64 | 237 |
|  | Lipophilic compounds (+) | 12262 | 382 | 904 | 1461 | 689 | 1836 |
|  |  | 43424 | 2701 | 1400 | 2375 | 1506 | 3438 |


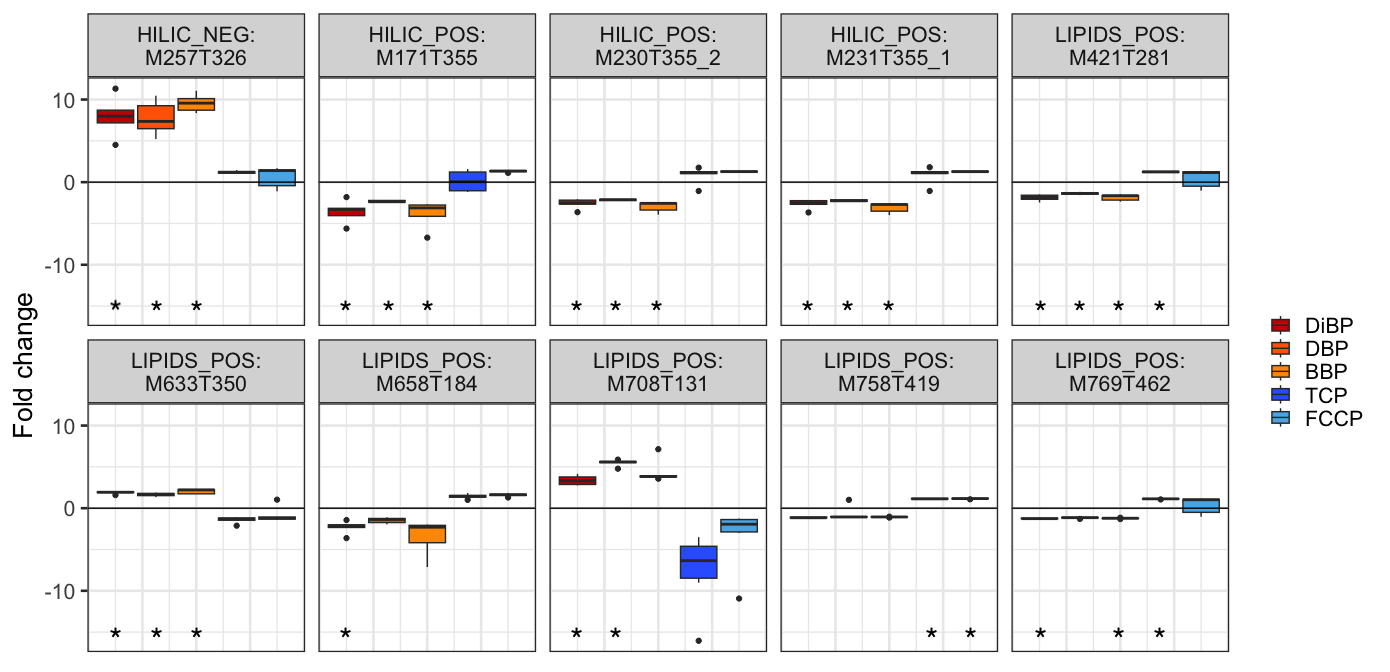


Figure S4. Fold-changes (-1/(log_2_ treatment – log_2_ control)) of top-ranked unannotated metabolite features derived from a partial least squares discriminant analysis (PLS-DA) model following high dose treatments of test substances benzyl butyl phthalate (BBP), dibutyl phthalate (DBP), diisobutyl phthalate (DiBP), 2, 3, 4, 5-tetrachlorophenol (TCP) and carbonyl cyanide 4-(trifluoromethoxy)phenylhydrazone (FCCP). Features are shown in descending (left to right) ranked order of VIP score. Asterisks below the box and whiskers indicate significant changes (q<0.05).

##### Section S4 – Metabolite and gene annotations

Table S6: Metabolite annotations for metabolomics features with VIP scores ≥ 1 using a PLS-DA model investigating the grouping of test substances benzyl butyl phthalate (BBP), dibutyl phthalate (DBP), diisobutyl phthalate (DiBP), 2,3,4,5-tetrachlorophenol (TCP) and carbonyl cyanide 4-(trifluoromethoxy)phenylhydrazone (FCCP). Metabolite annotations were either obtained using “mtoxcd” (where annotations were derived from either the MTox700+ spectral library or from Compound Discoverer) or “dma” (where annotations were derived from the DMA Galaxy workflow).

| Metabolite  feature | VIP score | Source | InChiKey | pubchem_cid | hmdb_id | kegg_id | Compound  name | Compound superclass | Compound  class | Compound  subclass |
| --- | --- | --- | --- | --- | --- | --- | --- | --- | --- | --- |
| LIPIDS_POS:M635T418 | 2.37 | dma | RUXSJGOKSGFEHU-JWNTXARASA-N | 60168070 | NA | NA | NA | Lipids and lipid-like molecules | Steroids and steroid derivatives | Stigmastanes and derivatives |
| LIPIDS_POS:M755T457 | 2.34 | dma | ZVUJGVPZCOYKGX-XYSYECETSA-N | 10395307 | NA | NA | NA | Lipids and lipid-like molecules | Sphingolipids | Glycosphingolipids |
| LIPIDS_POS:M372T89 | 2.33 | dma | PSHXNVGSVNEJBD-LJQANCHMSA-N | 53477791 | NA | NA | Tetradecanoylcarnitine | Lipids and lipid-like molecules | Fatty Acyls | Fatty acid esters |
| HILIC_POS:M129T519 | 2.25 | mtoxcd | KDXKERNSBIXSRK-UHFFFAOYSA-N | NA | HMDB0142894 | C16440 | DL-Lysine | Organic acids and derivatives | Carboxylic acids and derivatives | Amino acids, peptides, and analogues |
| HILIC_POS:M347T519 | 2.24 | mtoxcd | NA | NA | NA | NA | 2-{(3S)-1-[4-(Trifluoromethyl)benzyl]-3-pyrrolidinyl}-1, 3-benzoxazole |  |  |  |
| HILIC_POS:M130T519_5 | 2.2 | mtoxcd | KDXKERNSBIXSRK-UHFFFAOYSA-N | NA | HMDB0142894 | C16440 | DL-Lysine | Organic acids and derivatives | Carboxylic acids and derivatives | Amino acids, peptides, and analogues |
| HILIC_POS:M147T519_6 | 2.17 | mtoxcd | KDXKERNSBIXSRK-UHFFFAOYSA-N | NA | HMDB0142894 | C16440 | DL-Lysine | Organic acids and derivatives | Carboxylic acids and derivatives | Amino acids, peptides, and analogues |
| HILIC_POS:M169T519 | 2.14 | mtoxcd | KDXKERNSBIXSRK-UHFFFAOYSA-N | NA | HMDB0142894 | C16440 | DL-Lysine | Organic acids and derivatives | Carboxylic acids and derivatives | Amino acids, peptides, and analogues |
| LIPIDS_POS:M426T152 | 2.12 | dma | IPOLTUVFXFHAHI-WHIOSMTNSA-N | 46907933 | NA | NA | oleoyl-L-carnitine | Lipids and lipid-like molecules | Fatty Acyls | Fatty acid esters |
| LIPIDS_POS:M636T433 | 2.06 | dma | UYRNDQODBVPJMD-MVIDNBQJSA-N | 21778108 | NA | NA | NA | Lipids and lipid-like molecules | Sphingolipids | Ceramides |
| HILIC_POS:M88T515_2 | 2.04 | mtoxcd | LHIJANUOQQMGNT-UHFFFAOYSA-N | 8112 | NA | NA | Aminoethylethanolamine | Organic nitrogen compounds | Organonitrogen compounds | Amines |
| HILIC_NEG:M296T52 | 2.04 | mtoxcd | WUUGFSXJNOTRMR-IOSLPCCCSA-N | 439176 | HMDB0001173 | C00170 | 5'-Methylthioadenosine | Nucleosides, nucleotides, and analogues | 5'-deoxyribonucleosides | 5'-deoxy-5'-thionucleosides |
| LIPIDS_POS:M658T502 | 2.04 | dma | GAKUNXBDVGLOFS-WCCXBCNRSA-N | 87183063 | NA | NA | SCHEMBL974863 | Lipids and lipid-like molecules | Glycerolipids | Triradylcglycerols |
| HILIC_NEG:M134T52 | 2.02 | dma | GFFGJBXGBJISGV-UHFFFAOYSA-N | 190 | NA | NA | adenine | Organoheterocyclic compounds | Imidazopyrimidines | Purines and purine derivatives |
| LIPIDS_POS:M198T64 | 1.99 | mtoxcd | JHWNWJKBPDFINM-UHFFFAOYSA-N | 13690 | NA | NA | Laurolactam | Phenylpropanoids and polyketides | Macrolactams |  |
| HILIC_POS:M369T519_1 | 1.96 | mtoxcd | NA | NA | NA | NA | 2-{(3S)-1-[4-(Trifluoromethyl)benzyl]-3-pyrrolidinyl}-1, 3-benzoxazole |  |  |  |
| LIPIDS_POS:M886T565 | 1.95 | dma | ACTIUHUUMQJHFO-UPTCCGCDSA-N | 5281915 | NA | NA | Coenzyme Q10 | Lipids and lipid-like molecules | Prenol lipids | Quinone and hydroquinone lipids |
| HILIC_NEG:M209T439 | 1.94 | dma | BYXCFUMGEBZDDI-UHFFFAOYSA-N | 79437 | NA | NA | 1, 3, 7-Trimethyluric acid | Organoheterocyclic compounds | Imidazopyrimidines | Purines and purine derivatives |
| LIPIDS_POS:M864T565 | 1.94 | dma | ACTIUHUUMQJHFO-UPTCCGCDSA-N | 5281915 | NA | NA | Coenzyme Q10 | Lipids and lipid-like molecules | Prenol lipids | Quinone and hydroquinone lipids |
| LIPIDS_POS:M423T360 | 1.9 | dma | CAALPBCGPHIIHA-UHFFFAOYSA-N | 72728218 | NA | NA | 21-octacosenoic acid | Lipids and lipid-like molecules | Fatty Acyls | Fatty acids and conjugates |
| HILIC_POS:M184T621 | 1.85 | dma | YHHSONZFOIEMCP-UHFFFAOYSA-O | 1014 | NA | NA | phosphocholine | Organic nitrogen compounds | Organonitrogen compounds | Quaternary ammonium salts |
| HILIC_POS:M719T469 | 1.84 | dma | SRZJEPUYAKAFIE-RMAVLNRHSA-N | 1.38E+08 | NA | NA | Chondramide B | Organic acids and derivatives | Peptidomimetics | Hybrid peptides |
| LIPIDS_POS:M552T577 | 1.84 | dma | JEJLGIQLPYYGEE-UHFFFAOYSA-N | 99931 | NA | NA | 1, 2-Dipalmitoyl-rac-glycerol | Lipids and lipid-like molecules | Glycerolipids | Diradylglycerols |
| HILIC_POS:M258T486_6 | 1.83 | mtoxcd | SUHOQUVVVLNYQR-QMMMGPOBSA-N | NA | HMDB0000086 | C00670 | Glycerophosphocholine | Lipids and lipid-like molecules | Glycerophospholipids | Glycerophosphocholines |
| HILIC_POS:M147T464 | 1.82 | mtoxcd | ZDXPYRJPNDTMRX-UHFFFAOYSA-N | 738 | HMDB0013240 | C00303 | DL-Glutamine | Organic acids and derivatives | Carboxylic acids and derivatives | Amino acids, peptides, and analogues |
| HILIC_POS:M540T542 | 1.81 | dma | YIZLWMLZIYPRLH-UHFFFAOYSA-N | 10577363 | NA | NA | NA | Lipids and lipid-like molecules | Fatty Acyls | Fatty amides |
| HILIC_POS:M1013T572 | 1.8 | dma | OCIBBXPLUVYKCH-QXVNYKTNSA-N | 5288409 | NA | NA | Maltohexaose | Organic oxygen compounds | Organooxygen compounds | Carbohydrates and carbohydrate conjugates |
| LIPIDS_POS:M597T192 | 1.79 | mtoxcd | MQZIGYBFDRPAKN-UWFIBFSHSA-N | NA | HMDB0002204 | C08580 | Astaxanthin | Lipids and lipid-like molecules | Prenol lipids | Tetraterpenoids |
| LIPIDS_POS:M197T565 | 1.78 | mtoxcd | NA | NA | NA | NA | NP-019992 |  |  |  |
| LIPIDS_POS:M369T565 | 1.78 | dma | HVYWMOMLDIMFJA-DPAQBDIFSA-N | 5997 | NA | NA | cholesterol | Lipids and lipid-like molecules | Steroids and steroid derivatives | Cholestane steroids |
| LIPIDS_POS:M627T485 | 1.77 | dma | VLCIAVLHXXOWFZ-SBTMUPCMSA-N | 1.38E+08 | NA | NA | Myxovirescin C | Phenylpropanoids and polyketides | Macrolides and analogues |  |
| HILIC_NEG:M218T363_3 | 1.75 | dma | GHOKWGTUZJEAQD-ZETCQYMHSA-N | 6613 | NA | NA | pantothenic acid | Organic oxygen compounds | Organooxygen compounds | Alcohols and polyols |
| LIPIDS_NEG:M380T115 | 1.74 | dma | OIWTWACQMDFHJG-CCFUIAGSSA-N | 44251266 | NA | NA | Resolvin D1 | Lipids and lipid-like molecules | Fatty Acyls | Fatty acids and conjugates |
| LIPIDS_POS:M282T532 | 1.71 | mtoxcd | WWUZIQQURGPMPG-CCEZHUSRSA-N | NA | HMDB0000252 | C00319 | D-Sphingosine | Organic nitrogen compounds | Organonitrogen compounds | Amines |
| LIPIDS_POS:M992T508 | 1.7 | dma | PTRKLJDOSMBWQQ-TWMZOGGISA-N | 1.02E+08 | NA | NA | NA | Phenylpropanoids and polyketides | Macrolides and analogues |  |
| HILIC_POS:M183T484_2 | 1.69 | mtoxcd | PZMXDLWWQHYXGY-UHFFFAOYSA-N | 3338044 | NA | NA | 4-Amino-1-methyl-3-propyl-1H-pyrazole-5-carboxamide | Organic acids and derivatives | Carboxylic acids and derivatives | Carboxylic acid derivatives |
| LIPIDS_POS:M846T555 | 1.69 | dma | PGKWOUQKDDYJEI-AUNPKQNBSA-N | 6480645 | NA | NA | (6E, 10E)-3, 7, 11, 15, 19, 23, 27, 31, 35, 39-decamethyltetraconta-1, 6, 10, 38-tetraene-3, 15, 19, 23, 27, 31, 35-heptol | Lipids and lipid-like molecules | Prenol lipids | Polyterpenoids |
| LIPIDS_POS:M975T508 | 1.68 | dma | PTRKLJDOSMBWQQ-TWMZOGGISA-N | 1.02E+08 | NA | NA | NA | Phenylpropanoids and polyketides | Macrolides and analogues |  |
| LIPIDS_POS:M369T591 | 1.68 | dma | HVYWMOMLDIMFJA-DPAQBDIFSA-N | 5997 | NA | NA | cholesterol | Lipids and lipid-like molecules | Steroids and steroid derivatives | Cholestane steroids |
| LIPIDS_POS:M451T337 | 1.68 | dma | JQAUYFRMOXQSMM-UHFFFAOYSA-N | 1.23E+08 | NA | NA | NA | Lipids and lipid-like molecules | Fatty Acyls | Fatty alcohols |
| LIPIDS_POS:M367T397 | 1.67 | mtoxcd | NYOXRYYXRWJDKP-GYKMGIIDSA-N | 91477 | HMDB0000921 | C00599 | Cholest-4-en-3-one | Lipids and lipid-like molecules | Steroids and steroid derivatives | Cholestane steroids |
| LIPIDS_POS:M597T136_1 | 1.67 | mtoxcd | MQZIGYBFDRPAKN-UWFIBFSHSA-N | NA | HMDB0002204 | C08580 | Astaxanthin | Lipids and lipid-like molecules | Prenol lipids | Tetraterpenoids |
| HILIC_POS:M204T404_6 | 1.67 | mtoxcd | RDHQFKQIGNGIED-MRVPVSSYSA-N | 7045767 | HMDB0000201 | NA | Acetyl-L-carnitine | Lipids and lipid-like molecules | Fatty Acyls | Fatty acid esters |
| LIPIDS_POS:M282T542 | 1.65 | mtoxcd | WWUZIQQURGPMPG-CCEZHUSRSA-N | NA | HMDB0000252 | C00319 | D-Sphingosine | Organic nitrogen compounds | Organonitrogen compounds | Amines |
| LIPIDS_POS:M620T440 | 1.63 | dma | IENDTBZONILTAG-XKKJXBDVSA-N | 10438855 | NA | NA | NA | Lipids and lipid-like molecules | Fatty Acyls | Fatty amides |
| LIPIDS_POS:M369T602_2 | 1.63 | dma | HVYWMOMLDIMFJA-DPAQBDIFSA-N | 5997 | NA | NA | cholesterol | Lipids and lipid-like molecules | Steroids and steroid derivatives | Cholestane steroids |
| HILIC_POS:M393T407 | 1.62 | dma | HCPCZMCZJYSIAJ-GUBZILKMSA-N | 11360698 | NA | NA | CHEMBL507856 | Organic acids and derivatives | Carboxylic acids and derivatives | Amino acids, peptides, and analogues |
| LIPIDS_POS:M451T448 | 1.62 | dma | QXYCISFUJXCMSU-MDZDMXLPSA-N | 13132095 | NA | NA | Lumequic acid | Lipids and lipid-like molecules | Fatty Acyls | Fatty acids and conjugates |
| LIPIDS_POS:M182T36 | 1.62 | mtoxcd | IWKGJTDSJPLUCE-UHFFFAOYSA-N | 225550 | NA | NA | Triisopropanolamine cyclic borate | Organic nitrogen compounds | Organonitrogen compounds | Amines |
| LIPIDS_POS:M653T502 | 1.62 | dma | GAKUNXBDVGLOFS-WCCXBCNRSA-N | 87183063 | NA | NA | SCHEMBL974863 | Lipids and lipid-like molecules | Glycerolipids | Triradylcglycerols |
| LIPIDS_POS:M877T409 | 1.62 | dma | NPHUKIOGHFYZCW-OUUVCTIKSA-N | 12800561 | NA | NA | Neoviridogrisein I | Organic acids and derivatives | Peptidomimetics | Depsipeptides |
| HILIC_POS:M166T484 | 1.61 | mtoxcd | PZMXDLWWQHYXGY-UHFFFAOYSA-N | 3338044 | NA | NA | 4-Amino-1-methyl-3-propyl-1H-pyrazole-5-carboxamide | Organic acids and derivatives | Carboxylic acids and derivatives | Carboxylic acid derivatives |
| HILIC_POS:M130T455_5 | 1.6 | mtoxcd | ZDXPYRJPNDTMRX-UHFFFAOYSA-N | 738 | HMDB0013240 | C00303 | DL-Glutamine | Organic acids and derivatives | Carboxylic acids and derivatives | Amino acids, peptides, and analogues |
| HILIC_POS:M147T455_6 | 1.59 | mtoxcd | ZDXPYRJPNDTMRX-UHFFFAOYSA-N | 738 | HMDB0013240 | C00303 | DL-Glutamine | Organic acids and derivatives | Carboxylic acids and derivatives | Amino acids, peptides, and analogues |
| LIPIDS_POS:M282T520 | 1.59 | mtoxcd | WWUZIQQURGPMPG-CCEZHUSRSA-N | NA | HMDB0000252 | C00319 | D-Sphingosine | Organic nitrogen compounds | Organonitrogen compounds | Amines |
| HILIC_NEG:M221T50_3 | 1.59 | mtoxcd | YZBOVSFWWNVKRJ-UHFFFAOYSA-N | 8575 | HMDB0013247 | NA | Monobutyl phthalate | Benzenoids | Benzene and substituted derivatives | Benzoic acids and derivatives |
| HILIC_NEG:M122T291_2 | 1.59 | dma | PVNIIMVLHYAWGP-UHFFFAOYSA-N | 1.18E+08 | NA | NA | SCHEMBL16147135 | Organoheterocyclic compounds | Pyridines and derivatives | Pyridinecarboxylic acids and derivatives |
| LIPIDS_POS:M767T480 | 1.58 | dma | MWRBNPKJOOWZPW-NYVOMTAGSA-N | 44251425 | NA | NA | MWRBNPKJOOWZPW-NYVOMTAGSA-N | Lipids and lipid-like molecules | Glycerophospholipids | Glycerophosphoethanolamines |
| LIPIDS_POS:M642T495 | 1.56 | dma | GREDRAMJRDQWEJ-VCAYUJMESA-N | 95858727 | NA | NA | ZINC72400255 | Lipids and lipid-like molecules | Fatty Acyls | Lineolic acids and derivatives |
| HILIC_POS:M223T34_1 | 1.55 | dma | FLKPEMZONWLCSK-UHFFFAOYSA-N | 6781 | NA | NA | DIETHYL PHTHALATE | Benzenoids | Benzene and substituted derivatives | Benzoic acids and derivatives |
| HILIC_POS:M268T126_2 | 1.55 | mtoxcd | OIRDTQYFTABQOQ-KQYNXXCUSA-N | 60961 | HMDB0000050 | C00212 | Adenosine | Nucleosides, nucleotides, and analogues | Purine nucleosides |  |
| LIPIDS_POS:M282T508 | 1.55 | dma | JMXKCYUTURMERF-UHFFFAOYSA-N | 61899 | NA | NA | Dodemorph | Organoheterocyclic compounds | Oxazinanes | Morpholines |
| LIPIDS_POS:M887T486 | 1.54 | dma | FDTRCZDJIYMYRH-TVESKSJTSA-N | 11967363 | NA | NA | NA | Organic oxygen compounds | Organooxygen compounds | Carbohydrates and carbohydrate conjugates |
| HILIC_POS:M242T404 | 1.54 | mtoxcd | RDHQFKQIGNGIED-MRVPVSSYSA-N | 7045767 | HMDB0000201 | NA | Acetyl-L-carnitine | Lipids and lipid-like molecules | Fatty Acyls | Fatty acid esters |
| LIPIDS_POS:M282T553 | 1.52 | mtoxcd | WWUZIQQURGPMPG-CCEZHUSRSA-N | NA | HMDB0000252 | C00319 | D-Sphingosine | Organic nitrogen compounds | Organonitrogen compounds | Amines |
| HILIC_POS:M636T30 | 1.51 | dma | UYRNDQODBVPJMD-MVIDNBQJSA-N | 21778108 | NA | NA | NA | Lipids and lipid-like molecules | Sphingolipids | Ceramides |
| LIPIDS_POS:M720T598 | 1.5 | dma | GDFSBTHRBKRFTF-UHFFFAOYSA-N | 1.32E+08 | NA | NA | CE(DiMe(11, 5)) | Lipids and lipid-like molecules | Steroids and steroid derivatives | Steroid esters |
| LIPIDS_POS:M787T436 | 1.5 | dma | MPWUZHVZZKSTPV-MADBQMNMSA-N | 86289492 | NA | NA | 1-palmitoyl-2-docosahexaenoyl-sn-glycero-3-phosphoethanolamine | Lipids and lipid-like molecules | Glycerophospholipids | Glycerophosphoethanolamines |
| LIPIDS_POS:M369T579 | 1.49 | dma | HVYWMOMLDIMFJA-DPAQBDIFSA-N | 5997 | NA | NA | cholesterol | Lipids and lipid-like molecules | Steroids and steroid derivatives | Cholestane steroids |
| HILIC_POS:M205T484 | 1.48 | mtoxcd | PZMXDLWWQHYXGY-UHFFFAOYSA-N | 3338044 | NA | NA | 4-Amino-1-methyl-3-propyl-1H-pyrazole-5-carboxamide | Organic acids and derivatives | Carboxylic acids and derivatives | Carboxylic acid derivatives |
| LIPIDS_POS:M757T488_1 | 1.47 | mtoxcd | KILNVBDSWZSGLL-UHFFFAOYSA-N | 6138 | NA | NA | DL-Dipalmitoylphosphatidylcholine | Lipids and lipid-like molecules | Glycerophospholipids | Glycerophosphocholines |
| HILIC_POS:M241T427 | 1.46 | dma | WTOYNNBCKUYIKC-UHFFFAOYSA-N | 20797 | NA | NA | NOOTKATONE | Lipids and lipid-like molecules | Prenol lipids | Sesquiterpenoids |
| HILIC_POS:M377T270 | 1.45 | mtoxcd | AUNGANRZJHBGPY-SCRDCRAPSA-N | 493570 | HMDB0000244 | C00255 | Riboflavin | Organoheterocyclic compounds | Pteridines and derivatives | Alloxazines and isoalloxazines |
| LIPIDS_POS:M757T444_1 | 1.44 | dma | KILNVBDSWZSGLL-UHFFFAOYSA-N | 6138 | NA | NA | 2644-64-6 | Lipids and lipid-like molecules | Glycerophospholipids | Glycerophosphocholines |
| HILIC_POS:M189T528 | 1.44 | mtoxcd | NTNWOCRCBQPEKQ-YFKPBYRVSA-N | 132862 | HMDB0029416 | C03884 | L-Targinine | Organic acids and derivatives | Carboxylic acids and derivatives | Amino acids, peptides, and analogues |
| HILIC_POS:M179T377_2 | 1.43 | mtoxcd | LFUJIPVWTMGYDG-UHFFFAOYSA-N | 1340 | NA | NA | 1, 5-Isoquinolinediol | Organoheterocyclic compounds | Isoquinolines and derivatives |  |
| LIPIDS_POS:M1087T508 | 1.43 | dma | RYRBOTOSXJJGLX-YYUPCZRHSA-N | 10011412 | NA | NA | SCHEMBL2581241 | Organic acids and derivatives | Carboxylic acids and derivatives | Amino acids, peptides, and analogues |
| HILIC_NEG:M130T361_2 | 1.42 | dma | AGPKZVBTJJNPAG-WHFBIAKZSA-N | 7043901 | NA | NA | (2S, 3S)-2-ammonio-3-methylpentanoate | Organic acids and derivatives | Carboxylic acids and derivatives | Amino acids, peptides, and analogues |
| LIPIDS_POS:M728T520 | 1.42 | dma | ICFIZJQGJAJRSU-UHFFFAOYSA-N | 1157 | NA | NA | AC1L1AUT | Lipids and lipid-like molecules | Prenol lipids | Quinone and hydroquinone lipids |
| LIPIDS_POS:M857T481 | 1.42 | dma | SJGLOQSIMVYUEK-ZDDKLVSVSA-N | 10485678 | NA | NA | NA | Organic acids and derivatives | Carboxylic acids and derivatives | Amino acids, peptides, and analogues |
| LIPIDS_POS:M182T45 | 1.42 | dma | XBPCUCUWBYBCDP-UHFFFAOYSA-N | 7582 | NA | NA | DICYCLOHEXYLAMINE | Organic nitrogen compounds | Organonitrogen compounds | Cyclohexylamines |
| HILIC_POS:M754T504 | 1.42 | dma | HHJUWIANJFBDHT-KOTLKJBCSA-N | 40839 | NA | NA | vindesine | Alkaloids and derivatives | Vinca alkaloids |  |
| HILIC_POS:M367T455_1 | 1.41 | dma | JYWSARFDLXXOHT-UHFFFAOYSA-N | 45360332 | NA | NA | (-)-erythro-Anethole glycol 2-glucoside | Organic oxygen compounds | Organooxygen compounds | Carbohydrates and carbohydrate conjugates |
| HILIC_NEG:M606T526_5 | 1.41 | mtoxcd | LFTYTUAZOPRMMI-CFRASDGPSA-N | NA | HMDB0000290 | C00043 | UDP-N-acetylglucosamine | Nucleosides, nucleotides, and analogues | Pyrimidine nucleotides | Pyrimidine nucleotide sugars |
| HILIC_POS:M130T471 | 1.41 | dma | ODHCTXKNWHHXJC-VKHMYHEASA-N | 7405 | NA | NA | L-Pyroglutamic acid | Organic acids and derivatives | Carboxylic acids and derivatives | Amino acids, peptides, and analogues |
| HILIC_POS:M265T285 | 1.41 | mtoxcd | GJNDXQBALKCYSZ-UHFFFAOYSA-N | 144746 | NA | NA | 2-(2-amino-3-methylbutanamido)-3-phenylpropanoic acid | Organic acids and derivatives | Carboxylic acids and derivatives | Amino acids, peptides, and analogues |
| HILIC_POS:M225T377_2 | 1.41 | mtoxcd | VCKPUUFAIGNJHC-UHFFFAOYSA-N | NA | HMDB0000732 | C02794 | Hydroxykynurenine | Organic oxygen compounds | Organooxygen compounds | Carbonyl compounds |
| HILIC_POS:M249T522_2 | 1.4 | mtoxcd | XZWGNMZIFNOUGW-OGFXRTJISA-L | 23724807 | NA | D01611 | Carnosine |  |  |  |
| HILIC_POS:M148T471_5 | 1.4 | mtoxcd | WHUUTDBJXJRKMK-VKHMYHEASA-N | 33032 | HMDB0000148 | C00025 | L-Glutamic acid | Organic acids and derivatives | Carboxylic acids and derivatives | Amino acids, peptides, and analogues |
| HILIC_POS:M265T522 | 1.4 | mtoxcd | XZWGNMZIFNOUGW-OGFXRTJISA-L | 23724807 | NA | D01611 | Carnosine |  |  |  |
| HILIC_POS:M282T378 | 1.4 | dma | VQAYFKKCNSOZKM-IOSLPCCCSA-N | 102175 | NA | NA | N6-Methyladenosine | Nucleosides, nucleotides, and analogues | Purine nucleosides |  |
| HILIC_POS:M225T489 | 1.4 | mtoxcd | HVPFXCBJHIIJGS-LURJTMIESA-N | 169148 | NA | NA | N3, N4-Dimethyl-L-arginine | Organic acids and derivatives | Carboxylic acids and derivatives | Amino acids, peptides, and analogues |
| HILIC_POS:M766T518 | 1.4 | dma | WXHGVZMTCYBKFX-QPEQYQDCSA-N | 3037677 | NA | NA | BRN 0877058 | Organic acids and derivatives | Peptidomimetics | Hybrid peptides |
| HILIC_POS:M162T377_2 | 1.4 | mtoxcd | LFUJIPVWTMGYDG-UHFFFAOYSA-N | 1340 | NA | NA | 1, 5-Isoquinolinediol | Organoheterocyclic compounds | Isoquinolines and derivatives |  |
| LIPIDS_POS:M726T456 | 1.4 | mtoxcd | RWKUXQNLWDTSLO-GWQJGLRPSA-N | 9939941 | NA | NA | Palmitoyl sphingomyelin | Lipids and lipid-like molecules | Sphingolipids | Phosphosphingolipids |
| HILIC_POS:M131T471_1 | 1.39 | mtoxcd | WHUUTDBJXJRKMK-VKHMYHEASA-N | 33032 | HMDB0000148 | C00025 | L-Glutamic acid | Organic acids and derivatives | Carboxylic acids and derivatives | Amino acids, peptides, and analogues |
| LIPIDS_POS:M934T606 | 1.39 | dma | XGPILQZSXGDYEH-VBCYELTASA-N | 1.32E+08 | NA | NA | 1-Arachidonyl-2-oleoyl-3-a-linolenoyl-glycerol | Lipids and lipid-like molecules | Glycerolipids | Triradylcglycerols |
| HILIC_POS:M352T482_2 | 1.39 | dma | XJBBOFRVLANYNM-UHFFFAOYSA-N | 73198270 | NA | NA | NA | Organic acids and derivatives | Carboxylic acids and derivatives | Amino acids, peptides, and analogues |
| HILIC_POS:M237T431 | 1.39 | mtoxcd | GIAZPLMMQOERPN-UHFFFAOYSA-N | NA | HMDB0029135 | NA | Valylproline | Organic acids and derivatives | Carboxylic acids and derivatives | Amino acids, peptides, and analogues |
| LIPIDS_POS:M369T420 | 1.39 | dma | HVYWMOMLDIMFJA-DPAQBDIFSA-N | 5997 | NA | NA | cholesterol | Lipids and lipid-like molecules | Steroids and steroid derivatives | Cholestane steroids |
| HILIC_POS:M279T299_2 | 1.38 | mtoxcd | UDKICLZCJWQTLS-UHFFFAOYSA-N | 72137 | NA | NA | Tolycaine | Benzenoids | Benzene and substituted derivatives | Benzoic acids and derivatives |
| HILIC_POS:M162T429_7 | 1.38 | mtoxcd | PHIQHXFUZVPYII-UHFFFAOYSA-N | 288 | NA | NA | DL-Carnitine | Organic nitrogen compounds | Organonitrogen compounds | Quaternary ammonium salts |
| HILIC_NEG:M266T122_1 | 1.37 | dma | OIRDTQYFTABQOQ-KQYNXXCUSA-N | 60961 | NA | NA | adenosine | Nucleosides, nucleotides, and analogues | Purine nucleosides |  |
| HILIC_POS:M353T477 | 1.37 | dma | FHNINJWBTRXEBC-HXEHWPEMSA-N | NA | NA | NA | Sudan III | Organoheterocyclic compounds | Azobenzenes |  |
| HILIC_POS:M411T382 | 1.37 | dma | RAPZEAPATHNIPO-UHFFFAOYSA-N | 5073 | NA | NA | risperidone | Organoheterocyclic compounds | Pyridopyrimidines |  |
| HILIC_POS:M98T541 | 1.37 | mtoxcd | ONIBWKKTOPOVIA-SCSAIBSYSA-N | NA | HMDB0003411 | C00763 | D-(+)-Proline | Organic acids and derivatives | Carboxylic acids and derivatives | Amino acids, peptides, and analogues |
| HILIC_POS:M388T506 | 1.37 | dma | TXUZVZSFRXZGTL-UHFFFAOYSA-N | 63062 | NA | NA | Afimoxifene | Phenylpropanoids and polyketides | Stilbenes |  |
| HILIC_POS:M303T435 | 1.35 | mtoxcd | NA | NA | NA | NA | Î±-Aspartylphenylalanine |  |  |  |
| HILIC_POS:M189T547 | 1.34 | mtoxcd | MXNRLFUSFKVQSK-QMMMGPOBSA-N | NA | HMDB0001325 | C03793 | N6, N6, N6-Trimethyl-L-lysine | Organic acids and derivatives | Carboxylic acids and derivatives | Amino acids, peptides, and analogues |
| HILIC_POS:M237T386 | 1.34 | dma | OMNVYXHOSHNURL-WPRPVWTQSA-N | 6992394 | NA | NA | 3061-90-3 | Organic acids and derivatives | Carboxylic acids and derivatives | Amino acids, peptides, and analogues |
| LIPIDS_POS:M382T407 | 1.34 | dma | QVAALZYWZYXTTP-OCQYTUGVSA-N | 57418214 | NA | NA | (2Z, 6E)-3, 7, 11, 15, 19-Pentamethyl-2, 6-eicosadien-1-ol | Lipids and lipid-like molecules | Prenol lipids | Sesterterpenoids |
| HILIC_POS:M261T30_5 | 1.33 | dma | YUJSGIUJPLJCOO-UHFFFAOYSA-N | 44525694 | NA | NA | MMV023233 | Organoheterocyclic compounds | Quinolines and derivatives | Aminoquinolines and derivatives |
| HILIC_POS:M112T431 | 1.32 | mtoxcd | OPTASPLRGRRNAP-UHFFFAOYSA-N | 597 | HMDB0000630 | C00380 | Cytosine | Organoheterocyclic compounds | Diazines | Pyrimidines and pyrimidine derivatives |
| HILIC_POS:M225T494 | 1.32 | mtoxcd | HVPFXCBJHIIJGS-LURJTMIESA-N | 169148 | NA | NA | N3, N4-Dimethyl-L-arginine | Organic acids and derivatives | Carboxylic acids and derivatives | Amino acids, peptides, and analogues |
| HILIC_POS:M233T436 | 1.31 | dma | BQBCIBCLXBKYHW-UHFFFAOYSA-N | 4420322 | NA | NA | Thr-Leu | Organic acids and derivatives | Carboxylic acids and derivatives | Amino acids, peptides, and analogues |
| HILIC_POS:M265T330_1 | 1.31 | mtoxcd | GJNDXQBALKCYSZ-UHFFFAOYSA-N | 144746 | NA | NA | 2-(2-amino-3-methylbutanamido)-3-phenylpropanoic acid | Organic acids and derivatives | Carboxylic acids and derivatives | Amino acids, peptides, and analogues |
| LIPIDS_POS:M369T613 | 1.31 | dma | HVYWMOMLDIMFJA-DPAQBDIFSA-N | 5997 | NA | NA | cholesterol | Lipids and lipid-like molecules | Steroids and steroid derivatives | Cholestane steroids |
| HILIC_POS:M156T488 | 1.3 | mtoxcd | LMDZBCPBFSXMTL-UHFFFAOYSA-N | 15908 | NA | NA | 1-(3-Dimethylaminopropyl)-3-ethylcarbodiimide | Organic nitrogen compounds | Organonitrogen compounds | Amines |
| HILIC_POS:M241T457 | 1.3 | dma | WTOYNNBCKUYIKC-UHFFFAOYSA-N | 20797 | NA | NA | NOOTKATONE | Lipids and lipid-like molecules | Prenol lipids | Sesquiterpenoids |
| HILIC_POS:M167T144 | 1.29 | mtoxcd | QMQDJVIJVPEQHE-UHFFFAOYSA-N | 520098 | NA | NA | 2-sec-Butyl-3-methoxypyrazin | Organoheterocyclic compounds | Diazines | Pyrazines |
| HILIC_POS:M123T529 | 1.29 | mtoxcd | DFPAKSUCGFBDDF-UHFFFAOYSA-N | 936 | HMDB0001406 | C00153 | Nicotinamide | Organoheterocyclic compounds | Pyridines and derivatives | Pyridinecarboxylic acids and derivatives |
| HILIC_POS:M189T533_2 | 1.28 | mtoxcd | MXNRLFUSFKVQSK-QMMMGPOBSA-N | NA | HMDB0001325 | C03793 | N6, N6, N6-Trimethyl-L-lysine | Organic acids and derivatives | Carboxylic acids and derivatives | Amino acids, peptides, and analogues |
| HILIC_POS:M106T458_2 | 1.28 | mtoxcd | MTCFGRXMJLQNBG-UWTATZPHSA-N | 71077 | HMDB0003406 | C00740 | D-Serine | Organic acids and derivatives | Carboxylic acids and derivatives | Amino acids, peptides, and analogues |
| HILIC_POS:M116T521 | 1.28 | dma | ONIBWKKTOPOVIA-BYPYZUCNSA-N | 6971047 | NA | NA | L-Pro | Organic acids and derivatives | Carboxylic acids and derivatives | Amino acids, peptides, and analogues |
| LIPIDS_POS:M711T438 | 1.28 | dma | PIJBDQIAXDOOIW-RRSHUOBHSA-N | 21593887 | NA | NA | NA | Lipids and lipid-like molecules | Sphingolipids | Glycosphingolipids |
| HILIC_POS:M147T547 | 1.27 | mtoxcd | KDXKERNSBIXSRK-YFKPBYRVSA-N | 5962 | HMDB0000182 | C00047 | L-Lysine | Organic acids and derivatives | Carboxylic acids and derivatives | Amino acids, peptides, and analogues |
| HILIC_POS:M465T473 | 1.27 | dma | YJRWKOLRMSYZHE-UHFFFAOYSA-N | 53345476 | NA | NA | CHEMBL1784974 | Benzenoids | Naphthalenes |  |
| HILIC_POS:M630T541 | 1.27 | mtoxcd | LFTYTUAZOPRMMI-CFRASDGPSA-N | NA | HMDB0000290 | C00043 | UDP-N-acetylglucosamine | Nucleosides, nucleotides, and analogues | Pyrimidine nucleotides | Pyrimidine nucleotide sugars |
| LIPIDS_POS:M717T459_1 | 1.27 | dma | HBZNVZIRJWODIB-NHCUFCNUSA-N | 46891780 | NA | NA | L-alpha-Phosphatidylethanolamine (Soy) | Lipids and lipid-like molecules | Glycerophospholipids | Glycerophosphoethanolamines |
| LIPIDS_POS:M762T309 | 1.26 | dma | QIEGUUNVEUEFPY-UDEMINLESA-N | 16061236 | NA | NA | Adonixanthin 3'-beta-D-glucoside/ Adonixanthin 3'-glucoside | Lipids and lipid-like molecules | Prenol lipids | Tetraterpenoids |
| HILIC_NEG:M114T412 | 1.26 | dma | ONIBWKKTOPOVIA-BYPYZUCNSA-N | 6971047 | NA | NA | L-Pro | Organic acids and derivatives | Carboxylic acids and derivatives | Amino acids, peptides, and analogues |
| LIPIDS_POS:M279T314 | 1.25 | mtoxcd | DJDSLBVSSOQSLW-UHFFFAOYSA-N | 20393 | HMDB0013248 | C03343 | Mono(2-ethylhexyl) phthalate (MEHP) | Benzenoids | Benzene and substituted derivatives | Benzoic acids and derivatives |
| HILIC_POS:M280T486_2 | 1.25 | mtoxcd | SUHOQUVVVLNYQR-QMMMGPOBSA-N | NA | HMDB0000086 | C00670 | Glycerophosphocholine | Lipids and lipid-like molecules | Glycerophospholipids | Glycerophosphocholines |
| LIPIDS_POS:M857T457 | 1.25 | dma | DFDTZECTHJFPHE-YNHPNTTKSA-O | 23427125 | NA | NA | NA | Lipids and lipid-like molecules | Fatty Acyls | Fatty amides |
| HILIC_POS:M374T492_2 | 1.24 | mtoxcd | SULMJWJPVDSFFN-SNVBAGLBSA-N | 6398468 | NA | NA | INK | Benzenoids | Phenol ethers |  |
| HILIC_POS:M215T431_3 | 1.24 | mtoxcd | GIAZPLMMQOERPN-UHFFFAOYSA-N | NA | HMDB0029135 | NA | Valylproline | Organic acids and derivatives | Carboxylic acids and derivatives | Amino acids, peptides, and analogues |
| HILIC_POS:M122T60 | 1.23 | mtoxcd | CZZZABOKJQXEBO-UHFFFAOYSA-N | 7250 | NA | C11003 | 2, 4-Xylidine | Benzenoids | Benzene and substituted derivatives | Xylenes |
| HILIC_NEG:M121T35 | 1.23 | mtoxcd | RGHHSNMVTDWUBI-UHFFFAOYSA-N | 126 | HMDB0011718 | C00633 | 4-Hydroxybenzaldehyde | Organic oxygen compounds | Organooxygen compounds | Carbonyl compounds |
| LIPIDS_POS:M282T97 | 1.23 | mtoxcd | WWUZIQQURGPMPG-CCEZHUSRSA-N | NA | HMDB0000252 | C00319 | D-Sphingosine | Organic nitrogen compounds | Organonitrogen compounds | Amines |
| HILIC_NEG:M111T134_1 | 1.23 | mtoxcd | LFTYTUAZOPRMMI-CFRASDGPSA-N | NA | HMDB0000290 | C00043 | UDP-N-acetylglucosamine | Nucleosides, nucleotides, and analogues | Pyrimidine nucleotides | Pyrimidine nucleotide sugars |
| LIPIDS_POS:M587T432 | 1.23 | dma | YWTXVAUUCWGHSO-UHFFFAOYSA-N | 73029717 | NA | NA | Annotemoyin 1 | Lipids and lipid-like molecules | Fatty Acyls | Fatty alcohols |
| HILIC_NEG:M267T458 | 1.23 | mtoxcd | BKAYIFDRRZZKNF-VIFPVBQESA-N | 9903482 | NA | NA | N-Acetyl-L-carnosine | Organic acids and derivatives | Peptidomimetics | Hybrid peptides |
| HILIC_POS:M146T419_6 | 1.23 | dma | JHPNVNIEXXLNTR-UHFFFAOYSA-N | 725 | NA | NA | Actinine | Lipids and lipid-like molecules | Fatty Acyls | Fatty acids and conjugates |
| HILIC_NEG:M134T122_2 | 1.23 | mtoxcd | GFFGJBXGBJISGV-UHFFFAOYSA-N | 190 | HMDB0000034 | C00147 | Adenine | Organoheterocyclic compounds | Imidazopyrimidines | Purines and purine derivatives |
| HILIC_POS:M423T452 | 1.22 | mtoxcd | PRONOHBTMLNXCZ-UHFFFAOYSA-N | 18232044 | NA | NA | YLK | Organic acids and derivatives | Carboxylic acids and derivatives | Amino acids, peptides, and analogues |
| LIPIDS_POS:M903T453 | 1.22 | dma | DMUPZSDWJVULSC-UHFFFAOYSA-N | 76517846 | NA | NA | beta1-Tomatidine | Lipids and lipid-like molecules | Steroids and steroid derivatives | Steroidal glycosides |
| HILIC_NEG:M180T397_3 | 1.22 | mtoxcd | OUYCCCASQSFEME-QMMMGPOBSA-N | 6057 | HMDB0000158 | C00082 | L-Tyrosine | Organic acids and derivatives | Carboxylic acids and derivatives | Amino acids, peptides, and analogues |
| HILIC_NEG:M134T58 | 1.22 | dma | GFFGJBXGBJISGV-UHFFFAOYSA-N | 190 | NA | NA | adenine | Organoheterocyclic compounds | Imidazopyrimidines | Purines and purine derivatives |
| LIPIDS_POS:M629T605 | 1.21 | dma | NZGIKHMTWDNXAF-UHFFFAOYSA-N | 75295078 | NA | NA | cis-Reticulatacin-10-one | Lipids and lipid-like molecules | Fatty Acyls | Fatty alcohols |
| HILIC_POS:M220T101_4 | 1.21 | mtoxcd | GHOKWGTUZJEAQD-ZETCQYMHSA-N | 6613 | HMDB0000210 | C00864 | Pantothenic acid | Organic oxygen compounds | Organooxygen compounds | Alcohols and polyols |
| HILIC_POS:M197T510 | 1.2 | mtoxcd | ODKSFYDXXFIFQN-UHFFFAOYSA-N | 232 | NA | C02385 | DL-Arginine | Organic acids and derivatives | Carboxylic acids and derivatives | Amino acids, peptides, and analogues |
| HILIC_NEG:M243T297 | 1.2 | dma | BCVIOZZGJNOEQS-UHFFFAOYSA-N | 23274590 | NA | NA | NA | Organic acids and derivatives | Carboxylic acids and derivatives | Amino acids, peptides, and analogues |
| LIPIDS_POS:M632T107 | 1.2 | dma | QTLCLULKZUFQLC-YABIYNNUSA-N | 10483620 | NA | NA | NA | Phenylpropanoids and polyketides | Macrolide lactams |  |
| HILIC_NEG:M161T439 | 1.2 | dma | NPOAOTPXWNWTSH-UHFFFAOYSA-N | 1662 | NA | NA | MEGLUTOL | Lipids and lipid-like molecules | Fatty Acyls | Fatty acids and conjugates |
| HILIC_NEG:M132T529_3 | 1.2 | mtoxcd | CKLJMWTZIZZHCS-REOHCLBHSA-N | 5960 | HMDB0000191 | C00049 | L-Aspartic acid | Organic acids and derivatives | Carboxylic acids and derivatives | Amino acids, peptides, and analogues |
| HILIC_POS:M253T402 | 1.2 | mtoxcd | ALZVPLKYDKJKQU-XVKPBYJWSA-N | 92946 | HMDB0028699 | NA | Alanyltyrosine | Organic acids and derivatives | Carboxylic acids and derivatives | Amino acids, peptides, and analogues |
| LIPIDS_POS:M659T338 | 1.2 | dma | FMCTUMJBUUGELW-LVABKWSNSA-O | 1.01E+08 | NA | NA | NA | Lipids and lipid-like molecules | Fatty Acyls | Fatty acids and conjugates |
| HILIC_POS:M381T456 | 1.19 | dma | RCTGMCJBQGBLKT-PAMTUDGESA-N | NA | NA | NA | Sudan IV | Organoheterocyclic compounds | Azobenzenes |  |
| HILIC_NEG:M267T487_3 | 1.19 | dma | UGQMRVRMYYASKQ-KQYNXXCUSA-N | 1.35E+08 | NA | NA | inosine | Nucleosides, nucleotides, and analogues | Purine nucleosides |  |
| LIPIDS_POS:M1047T434 | 1.19 | dma | BXLIEUQCBKBVTC-OEBQGHFLSA-N | 10557983 | NA | NA | NA | Lipids and lipid-like molecules | Sphingolipids | Glycosphingolipids |
| HILIC_NEG:M201T402_2 | 1.19 | dma | RDIKFPRVLJLMER-UHFFFAOYSA-N | 259583 | NA | NA | dl-Alanyl-dl-leucine | Organic acids and derivatives | Carboxylic acids and derivatives | Amino acids, peptides, and analogues |
| HILIC_POS:M281T434 | 1.19 | mtoxcd | NA | NA | NA | NA | Î±-Aspartylphenylalanine |  |  |  |
| HILIC_POS:M291T453 | 1.18 | mtoxcd | BKAYIFDRRZZKNF-VIFPVBQESA-N | 9903482 | NA | NA | N-Acetyl-L-carnosine | Organic acids and derivatives | Peptidomimetics | Hybrid peptides |
| HILIC_POS:M159T266 | 1.18 | mtoxcd | FDGQSTZJBFJUBT-UHFFFAOYSA-N | 790 | HMDB0000157 | C00262 | Hypoxanthine | Organoheterocyclic compounds | Imidazopyrimidines | Purines and purine derivatives |
| HILIC_POS:M153T363_3 | 1.18 | dma | LRFVTYWOQMYALW-UHFFFAOYSA-N | 1188 | NA | NA | xanthine | Organoheterocyclic compounds | Imidazopyrimidines | Purines and purine derivatives |
| HILIC_NEG:M255T42 | 1.17 | mtoxcd | XDBMXUKHMOFBPJ-ZAFYKAAXSA-N | 54676864 | NA | NA | L-Ascorbic acid 2-sulfate | Organoheterocyclic compounds | Dihydrofurans | Furanones |
| HILIC_POS:M129T224 | 1.17 | dma | NBAKTGXDIBVZOO-UHFFFAOYSA-N | 93556 | NA | NA | Dihydrothymine | Organoheterocyclic compounds | Diazines | Pyrimidines and pyrimidine derivatives |
| HILIC_POS:M184T429 | 1.16 | mtoxcd | PHIQHXFUZVPYII-UHFFFAOYSA-N | 288 | NA | NA | DL-Carnitine | Organic nitrogen compounds | Organonitrogen compounds | Quaternary ammonium salts |
| LIPIDS_POS:M704T456_2 | 1.16 | mtoxcd | RWKUXQNLWDTSLO-GWQJGLRPSA-N | 9939941 | NA | NA | Palmitoyl sphingomyelin | Lipids and lipid-like molecules | Sphingolipids | Phosphosphingolipids |
| LIPIDS_POS:M611T491 | 1.15 | dma | BDGWQMLWIGDEKO-UHFFFAOYSA-N | 14759338 | NA | NA | Neoreticulatacin A | Lipids and lipid-like molecules | Fatty Acyls | Fatty alcohols |
| HILIC_POS:M119T397 | 1.15 | mtoxcd | IIBOGKHTXBPGEI-UHFFFAOYSA-N | 80654 | NA | C15561 | N-Benzylformamide | Benzenoids | Benzene and substituted derivatives |  |
| LIPIDS_POS:M968T430 | 1.15 | dma | IGYGGFRYWBWNTJ-IUNYHNGUSA-N | 16104249 | NA | NA | NA | Lipids and lipid-like molecules | Prenol lipids | Terpene lactones |
| HILIC_POS:M129T289 | 1.15 | mtoxcd | DPBWFNDFMCCGGJ-UHFFFAOYSA-N | 3772 | NA | NA | 4-Piperidinecarboxamide | Organoheterocyclic compounds | Piperidines | Piperidinecarboxylic acids and derivatives |
| HILIC_POS:M122T39 | 1.15 | mtoxcd | CZZZABOKJQXEBO-UHFFFAOYSA-N | 7250 | NA | C11003 | 2, 4-Xylidine | Benzenoids | Benzene and substituted derivatives | Xylenes |
| HILIC_NEG:M356T58 | 1.15 | dma | WUUGFSXJNOTRMR-IOSLPCCCSA-N | 439176 | NA | NA | Methylthioadenosine | Nucleosides, nucleotides, and analogues | 5'-deoxyribonucleosides | 5'-deoxy-5'-thionucleosides |
| HILIC_POS:M187T455_2 | 1.14 | mtoxcd | WPWUFUBLGADILS-WDSKDSINSA-N | 83525 | NA | NA | L-Alanyl-L-proline | Organic acids and derivatives | Carboxylic acids and derivatives | Amino acids, peptides, and analogues |
| HILIC_POS:M291T266 | 1.14 | mtoxcd | UGQMRVRMYYASKQ-KQYNXXCUSA-N | 6021 | HMDB0000195 | C00294 | Inosine | Nucleosides, nucleotides, and analogues | Purine nucleosides |  |
| HILIC_POS:M188T462 | 1.13 | mtoxcd | QEFRNWWLZKMPFJ-YGVKFDHGSA-N | 158980 | HMDB0002005 | NA | Methionine sulfoxide | Organic acids and derivatives | Carboxylic acids and derivatives | Amino acids, peptides, and analogues |
| HILIC_POS:M182T397 | 1.13 | mtoxcd | OUYCCCASQSFEME-QMMMGPOBSA-N | 6057 | HMDB0000158 | C00082 | L-Tyrosine | Organic acids and derivatives | Carboxylic acids and derivatives | Amino acids, peptides, and analogues |
| LIPIDS_POS:M393T34 | 1.13 | mtoxcd | GLZWNFNQMJAZGY-UHFFFAOYSA-N | 78798 | NA | NA | PEG n8 | Organic oxygen compounds | Organooxygen compounds | Ethers |
| LIPIDS_POS:M552T604 | 1.13 | dma | JEJLGIQLPYYGEE-UHFFFAOYSA-N | 99931 | NA | NA | 1, 2-Dipalmitoyl-rac-glycerol | Lipids and lipid-like molecules | Glycerolipids | Diradylglycerols |
| HILIC_POS:M154T275_2 | 1.12 | mtoxcd | XJWPISBUKWZALE-UHFFFAOYSA-N | 69602 | HMDB0013253 | C05135 | N-Acetylhistamine | Organic acids and derivatives | Carboxylic acids and derivatives | Carboxylic acid derivatives |
| LIPIDS_POS:M430T389 | 1.12 | dma | GVJHHUAWPYXKBD-IEOSBIPESA-N | 14985 | NA | NA | alpha-Tocopherol | Lipids and lipid-like molecules | Prenol lipids | Quinone and hydroquinone lipids |
| HILIC_POS:M275T402 | 1.12 | mtoxcd | ALZVPLKYDKJKQU-XVKPBYJWSA-N | 92946 | HMDB0028699 | NA | Alanyltyrosine | Organic acids and derivatives | Carboxylic acids and derivatives | Amino acids, peptides, and analogues |
| LIPIDS_POS:M737T548 | 1.12 | dma | JVMGRPXMVYGAQN-QYOQUFJESA-N | 6438769 | NA | NA | Glisoprenin B | Lipids and lipid-like molecules | Prenol lipids | Polyprenols |
| HILIC_POS:M307T266 | 1.11 | mtoxcd | UGQMRVRMYYASKQ-KQYNXXCUSA-N | 6021 | HMDB0000195 | C00294 | Inosine | Nucleosides, nucleotides, and analogues | Purine nucleosides |  |
| HILIC_POS:M136T397 | 1.11 | mtoxcd | IIBOGKHTXBPGEI-UHFFFAOYSA-N | 80654 | NA | C15561 | N-Benzylformamide | Benzenoids | Benzene and substituted derivatives |  |
| HILIC_POS:M365T464_3 | 1.11 | dma | GUBGYTABKSRVRQ-XLOQQCSPSA-N | 84571 | NA | NA | alpha-lactose | Organic oxygen compounds | Organooxygen compounds | Carbohydrates and carbohydrate conjugates |
| HILIC_POS:M185T455 | 1.11 | mtoxcd | ZDXPYRJPNDTMRX-UHFFFAOYSA-N | 738 | HMDB0013240 | C00303 | DL-Glutamine | Organic acids and derivatives | Carboxylic acids and derivatives | Amino acids, peptides, and analogues |
| LIPIDS_POS:M520T108 | 1.11 | mtoxcd | SPJFYYJXNPEZDW-FTJOPAKQSA-N | NA | HMDB0010386 | C04100 | LysoPC(18:2(9Z, 12Z)) | Lipids and lipid-like molecules | Glycerophospholipids | Glycerophosphocholines |
| HILIC_POS:M122T33 | 1.11 | mtoxcd | CZZZABOKJQXEBO-UHFFFAOYSA-N | 7250 | NA | C11003 | 2, 4-Xylidine | Benzenoids | Benzene and substituted derivatives | Xylenes |
| HILIC_POS:M227T542 | 1.1 | mtoxcd | XZWGNMZIFNOUGW-OGFXRTJISA-L | 23724807 | NA | D01611 | Carnosine |  |  |  |
| LIPIDS_POS:M745T480 | 1.1 | dma | MWRBNPKJOOWZPW-NYVOMTAGSA-N | 44251425 | NA | NA | MWRBNPKJOOWZPW-NYVOMTAGSA-N | Lipids and lipid-like molecules | Glycerophospholipids | Glycerophosphoethanolamines |
| HILIC_POS:M269T453 | 1.1 | mtoxcd | BKAYIFDRRZZKNF-VIFPVBQESA-N | 9903482 | NA | NA | N-Acetyl-L-carnosine | Organic acids and derivatives | Peptidomimetics | Hybrid peptides |
| LIPIDS_POS:M579T192 | 1.09 | mtoxcd | MQZIGYBFDRPAKN-UWFIBFSHSA-N | NA | HMDB0002204 | C08580 | Astaxanthin | Lipids and lipid-like molecules | Prenol lipids | Tetraterpenoids |
| HILIC_POS:M361T455 | 1.08 | dma | RPWFJAMTCNSJKK-UHFFFAOYSA-N | 14425 | NA | NA | Dodecyl gallate | Benzenoids | Benzene and substituted derivatives | Benzoic acids and derivatives |
| HILIC_NEG:M308T503_4 | 1.08 | dma | SQVRNKJHWKZAKO-PFQGKNLYSA-N | 445063 | NA | NA | 131-48-6 | Organic oxygen compounds | Organooxygen compounds | Carbohydrates and carbohydrate conjugates |
| HILIC_POS:M489T563 | 1.08 | mtoxcd | RZZPDXZPRHQOCG-OJAKKHQRSA-N | 13804 | NA | D00057 | Cytidine 5'-diphosphocholine | Nucleosides, nucleotides, and analogues | Pyrimidine nucleotides | Pyrimidine ribonucleotides |
| LIPIDS_POS:M1291T508 | 1.08 | dma | OCVAXCIPQXUQJH-XBXFPUCHSA-N | 16083114 | NA | NA | actinomycin G3 | Organic acids and derivatives | Peptidomimetics | Depsipeptides |
| HILIC_POS:M200T429 | 1.07 | mtoxcd | PHIQHXFUZVPYII-UHFFFAOYSA-N | 288 | NA | NA | DL-Carnitine | Organic nitrogen compounds | Organonitrogen compounds | Quaternary ammonium salts |
| HILIC_NEG:M164T345_4 | 1.07 | dma | COLNVLDHVKWLRT-QMMMGPOBSA-N | 6925665 | NA | NA | L-phenylalanine | Organic acids and derivatives | Carboxylic acids and derivatives | Amino acids, peptides, and analogues |
| LIPIDS_POS:M184T363 | 1.07 | dma | YHHSONZFOIEMCP-UHFFFAOYSA-O | 1014 | NA | NA | phosphocholine | Organic nitrogen compounds | Organonitrogen compounds | Quaternary ammonium salts |
| HILIC_POS:M329T324 | 1.07 | dma | JTVPZMFULRWINT-UHFFFAOYSA-N | 5467 | NA | NA | tiapride | Benzenoids | Benzene and substituted derivatives | Benzenesulfonyl compounds |
| LIPIDS_POS:M765T461 | 1.07 | dma | MPWUZHVZZKSTPV-MADBQMNMSA-N | 86289492 | NA | NA | 1-palmitoyl-2-docosahexaenoyl-sn-glycero-3-phosphoethanolamine | Lipids and lipid-like molecules | Glycerophospholipids | Glycerophosphoethanolamines |
| LIPIDS_POS:M542T108 | 1.07 | mtoxcd | SPJFYYJXNPEZDW-FTJOPAKQSA-N | NA | HMDB0010386 | C04100 | LysoPC(18:2(9Z, 12Z)) | Lipids and lipid-like molecules | Glycerophospholipids | Glycerophosphocholines |
| HILIC_POS:M365T458 | 1.06 | dma | GUBGYTABKSRVRQ-XLOQQCSPSA-N | 84571 | NA | NA | alpha-lactose | Organic oxygen compounds | Organooxygen compounds | Carbohydrates and carbohydrate conjugates |
| HILIC_NEG:M116T406 | 1.06 | mtoxcd | JJMDCOVWQOJGCB-UHFFFAOYSA-N | 138 | HMDB0003355 | C00431 | 5-Aminovaleric acid | Organic acids and derivatives | Carboxylic acids and derivatives | Amino acids, peptides, and analogues |
| HILIC_POS:M203T495_6 | 1.06 | mtoxcd | HVPFXCBJHIIJGS-LURJTMIESA-N | NA | HMDB0003334 | NA | Symmetric dimethylarginine | Organic acids and derivatives | Carboxylic acids and derivatives | Amino acids, peptides, and analogues |
| HILIC_POS:M213T510 | 1.05 | mtoxcd | ODKSFYDXXFIFQN-UHFFFAOYSA-N | 232 | NA | C02385 | DL-Arginine | Organic acids and derivatives | Carboxylic acids and derivatives | Amino acids, peptides, and analogues |
| HILIC_NEG:M146T515_2 | 1.05 | mtoxcd | WHUUTDBJXJRKMK-VKHMYHEASA-N | 33032 | HMDB0000148 | C00025 | L-Glutamic acid | Organic acids and derivatives | Carboxylic acids and derivatives | Amino acids, peptides, and analogues |
| HILIC_POS:M312T483 | 1.05 | mtoxcd | JCMUOFQHZLPHQP-BQBZGAKWSA-N | NA | HMDB0005765 | NA | Ophthalmic acid | Organic acids and derivatives | Carboxylic acids and derivatives | Amino acids, peptides, and analogues |
| HILIC_NEG:M915T454 | 1.05 | dma | JSVPJXYQXDNLRD-UHFFFAOYSA-N | 73792076 | NA | NA | NA | Organic acids and derivatives | Peptidomimetics | Depsipeptides |
| HILIC_NEG:M137T32 | 1.05 | mtoxcd | TYMLOMAKGOJONV-UHFFFAOYSA-N | 7475 | NA | C02126 | 4-Nitroaniline | Benzenoids | Benzene and substituted derivatives | Nitrobenzenes |
| HILIC_NEG:M319T529 | 1.05 | mtoxcd | NBSCHQHZLSJFNQ-QTVWNMPRSA-N | 65127 | NA | NA | D-Mannose 6-phosphate | Organic oxygen compounds | Organooxygen compounds | Carbohydrates and carbohydrate conjugates |
| LIPIDS_POS:M678T582 | 1.04 | dma | XDRZCIXEBLTNKS-UHFFFAOYSA-N | 1.32E+08 | NA | NA | CE(MonoMe(9, 5)) | Lipids and lipid-like molecules | Steroids and steroid derivatives | Steroid esters |
| LIPIDS_NEG:M452T132 | 1.04 | dma | YVYMBNSKXOXSKW-UHFFFAOYSA-N | 5256421 | NA | NA | AC1NQWNJ | Lipids and lipid-like molecules | Glycerophospholipids | Glycerophosphoethanolamines |
| HILIC_POS:M150T382_5 | 1.04 | mtoxcd | FFEARJCKVFRZRR-BYPYZUCNSA-N | 6137 | HMDB0000696 | C00073 | L-Methionine | Organic acids and derivatives | Carboxylic acids and derivatives | Amino acids, peptides, and analogues |
| HILIC_POS:M361T491 | 1.04 | mtoxcd | MECLEFZMPPOEAC-UHFFFAOYSA-N | 18224376 | NA | NA | TLK | Organic acids and derivatives | Carboxylic acids and derivatives | Amino acids, peptides, and analogues |
| LIPIDS_POS:M757T418 | 1.03 | dma | KILNVBDSWZSGLL-UHFFFAOYSA-N | 6138 | NA | NA | 2644-64-6 | Lipids and lipid-like molecules | Glycerophospholipids | Glycerophosphocholines |
| HILIC_POS:M133T382_3 | 1.03 | dma | FFEARJCKVFRZRR-BYPYZUCNSA-N | 6992087 | NA | NA | 3tun | Organic acids and derivatives | Carboxylic acids and derivatives | Amino acids, peptides, and analogues |
| LIPIDS_POS:M622T485 | 1.03 | dma | JLFRIOJUMJHQAO-TYAUXZOCSA-N | 21777635 | NA | NA | NA | Lipids and lipid-like molecules | Steroids and steroid derivatives | Bile acids, alcohols and derivatives |
| HILIC_POS:M128T113 | 1.02 | mtoxcd | NDNUANOUGZGEPO-UHFFFAOYSA-N | 9985 | NA | NA | Coniine | Alkaloids and derivatives |  |  |
| HILIC_POS:M120T341_4 | 1.02 | dma | LPAGFVYQRIESJQ-UHFFFAOYSA-N | 10328 | NA | NA | Indoline | Organoheterocyclic compounds | Indoles and derivatives | Indolines |
| HILIC_POS:M620T28 | 1.02 | dma | IENDTBZONILTAG-XKKJXBDVSA-N | 10438855 | NA | NA | NA | Lipids and lipid-like molecules | Fatty Acyls | Fatty amides |
| HILIC_POS:M394T332 | 1.02 | dma | NKANXQFJJICGDU-QPLCGJKRSA-N | 2733526 | NA | NA | tamoxifen | Phenylpropanoids and polyketides | Stilbenes |  |
| HILIC_POS:M110T547 | 1.02 | mtoxcd | GHCFWKFREBNSPC-UHFFFAOYSA-N | 7939 | NA | NA | 2-Amino-4-methylpyrimidine | Organoheterocyclic compounds | Diazines | Pyrimidines and pyrimidine derivatives |
| HILIC_POS:M166T341_5 | 1.02 | mtoxcd | COLNVLDHVKWLRT-QMMMGPOBSA-N | 6140 | HMDB0000159 | C00079 | L-Phenylalanine | Organic acids and derivatives | Carboxylic acids and derivatives | Amino acids, peptides, and analogues |
| HILIC_POS:M298T57_3 | 1.01 | mtoxcd | WUUGFSXJNOTRMR-IOSLPCCCSA-N | 439176 | HMDB0001173 | C00170 | 5'-Methylthioadenosine | Nucleosides, nucleotides, and analogues | 5'-deoxyribonucleosides | 5'-deoxy-5'-thionucleosides |
| LIPIDS_POS:M184T467 | 1.01 | dma | YHHSONZFOIEMCP-UHFFFAOYSA-O | 1014 | NA | NA | phosphocholine | Organic nitrogen compounds | Organonitrogen compounds | Quaternary ammonium salts |
| HILIC_POS:M248T483 | 1.01 | dma | IAWKAVWGVXBNLH-UHFFFAOYSA-N | 14101166 | NA | NA | (2S, 3'S)-alpha-Amino-2-carboxy-5-oxo-1-pyrrolidinebutanoic acid | Organic acids and derivatives | Carboxylic acids and derivatives | Amino acids, peptides, and analogues |
| LIPIDS_POS:M679T428 | 1.01 | dma | CITHEXJVPOWHKC-UHFFFAOYSA-N | 26197 | NA | NA | 18656-38-7 | Lipids and lipid-like molecules | Glycerophospholipids | Glycerophosphocholines |
| HILIC_POS:M463T489 | 1.01 | dma | UFPLHRPHJDMHRW-GTKOJQRLSA-N | 1.37E+08 | NA | NA | NA | Organoheterocyclic compounds | Pteridines and derivatives | Pterins and derivatives |
| HILIC_POS:M355T470 | 1.01 | dma | GVQWKADEEQMGQO-UHFFFAOYSA-N | 45859661 | NA | NA | MMV688796 | Organic acids and derivatives | Carboxylic acids and derivatives | Carboxylic acid derivatives |
| HILIC_POS:M229T447_2 | 1.01 | mtoxcd | YCYXUKRYYSXSLJ-CVEARBPZSA-N | 173815 | NA | NA | Prolylleucine | Organic acids and derivatives | Carboxylic acids and derivatives | Amino acids, peptides, and analogues |
| HILIC_POS:M95T445_2 | 1.01 | mtoxcd | OSSNTDFYBPYIEC-UHFFFAOYSA-N | 66171 | NA | NA | 1-Vinylimidazole | Organoheterocyclic compounds | Azoles | Imidazoles |
| HILIC_POS:M138T397_6 | 1 | mtoxcd | WWNNZCOKKKDOPX-UHFFFAOYSA-N | 5570 | HMDB0000875 | C01004 | Trigonelline | Alkaloids and derivatives |  |  |
| HILIC_POS:M169T455_2 | 1 | mtoxcd | ZDXPYRJPNDTMRX-VKHMYHEASA-N | 5961 | HMDB0000641 | C00064 | L-Glutamine | Organic acids and derivatives | Carboxylic acids and derivatives | Amino acids, peptides, and analogues |

Table S7: Gene annotations with human orthologs for transcriptomic features with VIP scores ≥ 1 using a PLS-DA model investigating the grouping of test substances benzyl butyl phthalate (BBP), dibutyl phthalate (DBP), diisobutyl phthalate (DiBP), 2,3,4,5-tetrachlorophenol (TCP) and carbonyl cyanide 4-(trifluoromethoxy)phenylhydrazone (FCCP). Human Gene Orthologs lists the mapping of each Daphnia gene to their orthologs in humans within the OrthoDB Database v10 (Kriventseva et al., 2019). Those listed as ‘NA’ those from gene families that are unique to the Daphnia phylogenetic lineage.

| **Gene Feature** | **VIP Score** | **Human Gene Orthologs** |
| --- | --- | --- |
| **RNA:Dapma7bEVm009578** | 2.33 | PFKM, PFKP, PFKL |
| **RNA:Dapma7bEVm006728** | 2.23 | ACHE |
| **RNA:Dapma7bEVm000940** | 2.19 | TUBB, TUBB2B, TUBB2A, TUBB4B, TUBB8, TUBB3, TUBB6, TUBB8P12, TUBB4A, TUBB1 |
| **RNA:Dapma7bEVm001565** | 2.18 | CPB2, CPA2, CPA1, CPA4, CPA5, CPA6, CPO, CPB1, CPA3 |
| **RNA:Dapma7bEVm001991** | 2.17 | EPHX2, EPHX4, EPHX3 |
| **RNA:Dapma7bEVm001910** | 2.16 | CNDP2, CNDP1 |
| **RNA:Dapma7bEVm010420** | 2.14 | RCN2, CALU, RCN1, RCN3 |
| **RNA:Dapma7bEVm001597** | 2.12 | PPA2, PPA1 |
| **RNA:Dapma7bEVm025783** | 2.11 | NA |
| **RNA:Dapma7bEVm004847** | 2.1 | UGT2B28, UGT2B15, UGT2A3, UGT2B4, UGT2B11, UGT2B7, UGT2B17, UGT2A1, UGT8, UGT2B10, UGT3A2, UGT3A1, UGA7, UGA10, UGA4, UGA8, UGA9, UGA1, UGA6, UGA3, UGA5, LOC101929773, UGT2A3 |
| **RNA:Dapma7bEVm005500** | 2.08 | CHRNB4, CHRNA5, CHRNA3, CHRNA6, CHRNB3, CHRNB3, CHRNA2, CHRNB2, CHRNA1, CHRNA4 |
| **RNA:Dapma7bEVm000028** | 2.05 | RAP1GAP2, RAP1GAP2, RAP1GAP |
| **RNA:Dapma7bEVm010404** | 2.02 | MDH2 |
| **RNA:Dapma7bEVm006057** | 2.02 | MDM2, MDM4 |
| **RNA:Dapma7bEVm004767** | 2.02 | PGK2, PGK1 |
| **RNA:Dapma7bEVm006826** | 2.01 | UBE2T |
| **RNA:Dapma7bEVm003643** | 1.96 | PRKCZ, PRKCI |
| **RNA:Dapma7bEVm002197** | 1.93 | TWISTNB |
| **RNA:Dapma7bEVm003343** | 1.92 | SLC35A1, SLC35A3, SLC35A3, SLC35A2 |
| **RNA:Dapma7bEVm002681** | 1.91 | HNF4G, HNF4A, HNF4A |
| **RNA:Dapma7bEVm005607** | 1.9 | SUCLA2, SUCLG2 |
| **RNA:Dapma7bEVm015298** | 1.86 | PRSS1, PRSS2, PRSS1, PRSS3, PRSS3, KLK9, KLK10, PRSS38, KLK3, KLK4, KLK12, AZU1, PRTN3, ELANE, KLK11, KLK15 |
| **RNA:Dapma7bEVm009722** | 1.85 | TRAFD1, XAF1 |
| **RNA:Dapma7bEVm001152** | 1.85 | CCT2, TCP1, CCT7 |
| **RNA:Dapma7bEVm007452** | 1.84 | AQP9, AQP9, AQP3, LOC112267859, AQP7, AQP10, LOC100509620 |
| **RNA:Dapma7bEVm006156** | 1.82 | NA |
| **RNA:Dapma7bEVm000324** | 1.8 | ENDOU |
| **RNA:Dapma7bEVm018551** | 1.79 | ACTN1, ACTN3, ACTN2, ACTN4, ACTN4 |
| **RNA:Dapma7bEVm000660** | 1.79 | PLOD3, PLOD1, PLOD2 |
| **RNA:Dapma7bEVm004944** | 1.79 | GSTM4, GSTM5, GSTM2, GSTM3, GSTM1 |
| **RNA:Dapma7bEVm017213** | 1.78 | B4GAL, B4GALT6, B4GALT2, B4GALT3, B4GALT4, B4GALT5 |
| **RNA:Dapma7bEVm011067** | 1.78 | TSTA3 |
| **RNA:Dapma7bEVm001287** | 1.77 | SFTPA1, SFTPA2 |
| **RNA:Dapma7bEVm000029** | 1.76 | ALDH3B1, ALDH3B2, ALDH3A1, ALDH3A2 |
| **RNA:Dapma7bEVm012147** | 1.74 | CNPY3, CNPY4 |
| **RNA:Dapma7bEVm004355** | 1.73 | TBPL2, TBP |
| **RNA:Dapma7bEVm015122** | 1.73 | QARS, EPRS |
| **RNA:Dapma7bEVm004697** | 1.72 | CYP3A7-CYP3A51P, TBXAS1, CYP3A4, CYP3A43, CYP3A7, TBXAS1, CYP3A5 |
| **RNA:Dapma7bEVm017578** | 1.72 | CLEC19A, REG4, REG1B, REG3G, REG3A, REG1A, CLEC3B, CLEC4M |
| **RNA:Dapma7bEVm004606** | 1.72 | HSPA9, HSPA5 |
| **RNA:Dapma7bEVm010599** | 1.72 | HSPD1 |
| **RNA:Dapma7bEVm005570** | 1.71 | UGDH |
| **RNA:Dapma7bEVm000321** | 1.7 | CYP3A7-CYP3A51P, TBXAS1, CYP3A4, CYP3A43, CYP3A7, TBXAS1, CYP3A5 |
| **RNA:Dapma7bEVm010950** | 1.7 | MAP2K1, MAP2K1, MAP2K5, MAP2K2 |
| **RNA:Dapma7bEVm027932** | 1.69 | CTH |
| **RNA:Dapma7bEVm012202** | 1.69 | ATP6V1G2, ATP6V1G1, ATP6V1G3, ATP6V1G3 |
| **RNA:Dapma7bEVm012440** | 1.69 | TRIAP1 |
| **RNA:Dapma7bEVm001650** | 1.69 | NA |
| **RNA:Dapma7bEVm002124** | 1.68 | CEBPZ |
| **RNA:Dapma7bEVm006218** | 1.68 | INPP5F, INPP5F, INPP5F, INPP5F, SACM1L |
| **RNA:Dapma7bEVm002808** | 1.68 | BUB3, RAE1 |
| **RNA:Dapma7bEVm008240** | 1.68 | PCBD2, PCBD1 |
| **RNA:Dapma7bEVm002731** | 1.68 | EIF3E |
| **RNA:Dapma7bEVm000066** | 1.68 | AMY1A, AMY1B, AMY2B, AMY1C, AMY2A |
| **RNA:Dapma7bEVm027498** | 1.67 | C4B, CD109, C4A, A2M, PZP, PZP, A2ML1, C5, CPAMD8, LOC110384692, C4B_2 |
| **RNA:Dapma7bEVm015760** | 1.66 | NA |
| **RNA:Dapma7bEVm005357** | 1.66 | NA |
| **RNA:Dapma7bEVm002457** | 1.66 | CA2, CA3, CA8, CA13, CA5A, CA7, CA5B |
| **RNA:Dapma7bEVm015242** | 1.65 | NNT |
| **RNA:Dapma7bEVm007625** | 1.65 | RFC3 |
| **RNA:Dapma7bEVm001618** | 1.64 | H1FX, H1F0 |
| **RNA:Dapma7bEVm009919** | 1.64 | RPLP0 |
| **RNA:Dapma7bEVm000568** | 1.63 | DNAJA3 |
| **RNA:Dapma7bEVm003885** | 1.63 | CTSG, GZMH, GZMB, CMA1 |
| **RNA:Dapma7bEVm004468** | 1.63 | RSL24D1 |
| **RNA:Dapma7bEVm009686** | 1.63 | CLGN, CANX, CANX |
| **RNA:Dapma7bEVm010991** | 1.62 | LYZL4, LALBA, LYZ, LYZL1, LYZL2, SPACA3, LYZL6, SPACA5B, SPACA5 |
| **RNA:Dapma7bEVm007445** | 1.62 | TMPRSS11E, S4, TMPRSS5, PRSS53, PRSS36, KLK1, PRSS43, TMPRSS7, TMPRSS9, KLK13, KLK8, KLK7, KLK2, KLK6, KLK14, KLK5, TMPRSS6 |
| **RNA:Dapma7bEVm015452** | 1.62 | KCNK9, KCNK3, KCNK15 |
| **RNA:Dapma7bEVm010270** | 1.62 | DCP1B, DCP1A |
| **RNA:Dapma7bEVm000253** | 1.62 | CPB2, CPA2, CPA1, CPA4, CPA5, CPA6, CPO, CPB1, CPA3 |
| **RNA:Dapma7bEVm006041** | 1.62 | EXOSC4, EXOSC4 |
| **RNA:Dapma7bEVm003836** | 1.62 | ATG3 |
| **RNA:Dapma7bEVm007439** | 1.62 | B4GAL, B4GALT6, B4GALT2, B4GALT3, B4GALT4, B4GALT5 |
| **RNA:Dapma7bEVm019267** | 1.62 | TUBA1C, TUBA1A, TUBA1B, TUBA3C, TUBAL3, TUBA4A, TUBA3D, TUBA3E, TUBA8 |
| **RNA:Dapma7bEVm002635** | 1.6 | NFKBIE, NFKBIA, NFKBIB, BCL3 |
| **RNA:Dapma7bEVm008091** | 1.6 | SRSF3, SRSF7 |
| **RNA:Dapma7bEVm000542** | 1.6 | C1QTNF7, C1QTNF2, C1QTNF9, C1QTNF9B, C1QB, C1QC, C1QA, ADIPOQ, OTOL1 |
| **RNA:Dapma7bEVm003260** | 1.6 | ATP6AP1L, ATP6AP1 |
| **RNA:Dapma7bEVm015646** | 1.6 | RSU1 |
| **RNA:Dapma7bEVm008232** | 1.59 | HSPB3, HSPB8, HSPB1, CRYAB, HSPB2, HSPB6, CRYAA, CRYAA2 |
| **RNA:Dapma7bEVm004317** | 1.59 | TOR1A, TOR1B, TOR2A, TOR3A |
| **RNA:Dapma7bEVm010446** | 1.58 | MYL6, MYL6B, MYL4, MYL1, MYL3 |
| **RNA:Dapma7bEVm006832** | 1.58 | SGCB |
| **RNA:Dapma7bEVm008403** | 1.57 | NA |
| **RNA:Dapma7bEVm011894** | 1.57 | NA |
| **RNA:Dapma7bEVm010028** | 1.56 | PRKG2, PRKG1, PRKG1 |
| **RNA:Dapma7bEVm006239** | 1.56 | LIPN, LIPK, LIPJ, LIPK, LIPF, LIPM, LIPA |
| **RNA:Dapma7bEVm001292** | 1.55 | PRSS1, PRSS2, PRSS1, PRSS3, PRSS3, KLK9, KLK10, PRSS38, KLK3, KLK4, KLK12, AZU1, PRTN3, ELANE, KLK11, KLK15 |
| **RNA:Dapma7bEVm012434** | 1.55 | FUT9, FUT7, FUT4, FUT6, FUT3, FUT5 |
| **RNA:Dapma7bEVm002584** | 1.55 | RASA1, SYNGAP1, DAB2IP, DAB2IP, RASAL2, RASAL2, RASAL2, RASAL3 |
| **RNA:Dapma7bEVm000604** | 1.55 | COL4A1, COL4A2, COL4A3, COL4A3, COL4A4, COL4A5, COL4A6 |
| **RNA:Dapma7bEVm010157** | 1.55 | HMG20A, HMG20B |
| **RNA:Dapma7bEVm001511** | 1.55 | AMY1A, AMY1B, AMY2B, AMY1C, AMY2A |
| **RNA:Dapma7bEVm002225** | 1.54 | SLC6A3, SLC6A2, SLC6A4 |
| **RNA:Dapma7bEVm006095** | 1.54 | CLTB, CLTA |
| **RNA:Dapma7bEVm002389** | 1.53 | TRAM1L1, TRAM2, TRAM1 |
| **RNA:Dapma7bEVm003942** | 1.53 | PSMD9 |
| **RNA:Dapma7bEVm005960** | 1.52 | STAMBPL1, STAMBP |
| **RNA:Dapma7bEVm007127** | 1.52 | DHFR, DHFR2 |
| **RNA:Dapma7bEVm001265** | 1.52 | GGH |
| **RNA:Dapma7bEVm004389** | 1.52 | SSR1 |
| **RNA:Dapma7bEVm001764** | 1.51 | LIG1 |
| **RNA:Dapma7bEVm012375** | 1.51 | NA |
| **RNA:Dapma7bEVm005288** | 1.51 | EIF2S2 |
| **RNA:Dapma7bEVm007606** | 1.51 | PAX4, PAX6 |
| **RNA:Dapma7bEVm001683** | 1.5 | CLTC, CLTCL1, CLTCL1 |
| **RNA:Dapma7bEVm004661** | 1.5 | CTTN, HCLS1 |
| **RNA:Dapma7bEVm027493** | 1.49 | ACADVL, ACAD9 |
| **RNA:Dapma7bEVm000023** | 1.49 | MPP6, MPP2, CASK, MPP1 |
| **RNA:Dapma7bEVm000190** | 1.49 | HEXB, HEXA |
| **RNA:Dapma7bEVm010840** | 1.48 | PPOX |
| **RNA:Dapma7bEVm012905** | 1.48 | PIN1 |
| **RNA:Dapma7bEVm002345** | 1.48 | GZMK, GZMA |
| **RNA:Dapma7bEVm010120** | 1.48 | RGL2, RALGDS, RGL1, RGL3, RGL4, RGL2 |
| **RNA:Dapma7bEVm010925** | 1.47 | CYP1B1 |
| **RNA:Dapma7bEVm003217** | 1.47 | BIRC2, BIRC3, BIRC8, BIRC7, XIAP |
| **RNA:Dapma7bEVm005672** | 1.47 | COPS5 |
| **RNA:Dapma7bEVm002275** | 1.46 | CCT2, TCP1, CCT7 |
| **RNA:Dapma7bEVm014955** | 1.46 | CYP27B1, CYP11A1, CYP11B2, CYP11B1, CYP27A1, CYP27C1, CYP24A1 |
| **RNA:Dapma7bEVm015772** | 1.46 | SLC35A1, SLC35A3, SLC35A3, SLC35A2 |
| **RNA:Dapma7bEVm003519** | 1.45 | INPP5F, INPP5F, INPP5F, INPP5F, SACM1L |
| **RNA:Dapma7bEVm003994** | 1.45 | DHX29, YTHDC2, DHX9, DHX57, DHX36, DHX30, DHX34 |
| **RNA:Dapma7bEVm004102** | 1.45 | PDIA5 |
| **RNA:Dapma7bEVm002577** | 1.44 | NFKB1, NFKB2, RELA, REL, RELB |
| **RNA:Dapma7bEVm010807** | 1.44 | DAG1 |
| **RNA:Dapma7bEVm006813** | 1.44 | RBKS |
| **RNA:Dapma7bEVm001185** | 1.44 | LIPN, LIPK, LIPJ, LIPK, LIPF, LIPM, LIPA |
| **RNA:Dapma7bEVm001389** | 1.43 | MARS |
| **RNA:Dapma7bEVm010997** | 1.43 | NA |
| **RNA:Dapma7bEVm001227** | 1.43 | MSMO1, SC5D |
| **RNA:Dapma7bEVm001483** | 1.43 | ZWILCH |
| **RNA:Dapma7bEVm010152** | 1.43 | AHCY |
| **RNA:Dapma7bEVm011678** | 1.42 | CDA |
| **RNA:Dapma7bEVm008302** | 1.42 | DAD1 |
| **RNA:Dapma7bEVm005737** | 1.42 | RAB27A, RAB27B |
| **RNA:Dapma7bEVm006949** | 1.42 | HISH3E, HISH3C, HISH3J, HISH3H, HISH3B, HISH3F, HISH3D, HISH3G, HISH3I, HISH3A, HIST2H3PS2 |
| **RNA:Dapma7bEVm000522** | 1.42 | ATP6V1A |
| **RNA:Dapma7bEVm003069** | 1.42 | DDX46, DDX42 |
| **RNA:Dapma7bEVm010786** | 1.42 | FKBP14, FKBP7 |
| **RNA:Dapma7bEVm010637** | 1.42 | NA |
| **RNA:Dapma7bEVm004878** | 1.41 | PHYHD1 |
| **RNA:Dapma7bEVm027414** | 1.41 | ACE, ACE2 |
| **RNA:Dapma7bEVm012830** | 1.41 | RPA3 |
| **RNA:Dapma7bEVm004622** | 1.41 | MOXD1, DBH |
| **RNA:Dapma7bEVm003189** | 1.41 | SCARB2, SCARB1, CD36 |
| **RNA:Dapma7bEVm004188** | 1.41 | NR1I2, VDR, NR1I3, NR1I2 |
| **RNA:Dapma7bEVm001237** | 1.41 | TXNDC5 |
| **RNA:Dapma7bEVm002983** | 1.41 | RAN |
| **RNA:Dapma7bEVm028401** | 1.41 | IGF1R, IGF1R, INSRR, INSR |
| **RNA:Dapma7bEVm007328** | 1.4 | BCAS2 |
| **RNA:Dapma7bEVm005460** | 1.4 | MCM9, MCM3, MCM7, MCM9, MCM4, MCM6, MCM2, MCM8, MCM5 |
| **RNA:Dapma7bEVm001672** | 1.4 | RGPD1, RANBP2, RGPD4, RGPD6, RGPD1, RGPD2, RGPD5, RGPD3 |
| **RNA:Dapma7bEVm020549** | 1.4 | UBC, UBB, RPS27A, UBA52 |
| **RNA:Dapma7bEVm002724** | 1.4 | ALG8, ALG6 |
| **RNA:Dapma7bEVm010835** | 1.39 | NOP58, NOP56 |
| **RNA:Dapma7bEVm000221** | 1.39 | VARS2, VARS, VARS |
| **RNA:Dapma7bEVm002000** | 1.39 | LAMP1, LAMP2 |
| **RNA:Dapma7bEVm015164** | 1.38 | DHFR, DHFR2 |
| **RNA:Dapma7bEVm018640** | 1.38 | ATP6V0C |
| **RNA:Dapma7bEVm005263** | 1.38 | B3GALT6 |
| **RNA:Dapma7bEVm012813** | 1.38 | CDC6, ORC1 |
| **RNA:Dapma7bEVm000194** | 1.38 | NT5E |
| **RNA:Dapma7bEVm002915** | 1.37 | GSTA4 |
| **RNA:Dapma7bEVm018434** | 1.37 | CREBBP, EP300 |
| **RNA:Dapma7bEVm012511** | 1.37 | NA |
| **RNA:Dapma7bEVm003922** | 1.37 | GSTZ1 |
| **RNA:Dapma7bEVm010714** | 1.37 | P4HA2, P4HA1, P4HA3 |
| **RNA:Dapma7bEVm005713** | 1.37 | CBR3 |
| **RNA:Dapma7bEVm002349** | 1.37 | EEF1G |
| **RNA:Dapma7bEVm011742** | 1.37 | SH3BP5, SH3BP5L |
| **RNA:Dapma7bEVm008161** | 1.37 | LSM2 |
| **RNA:Dapma7bEVm010357** | 1.37 | PUM3 |
| **RNA:Dapma7bEVm025189** | 1.36 | RPA2, RPA4 |
| **RNA:Dapma7bEVm003309** | 1.36 | PSMD4 |
| **RNA:Dapma7bEVm001679** | 1.36 | ABCA13, ABCA1, ABCA2, ABCA3, ABCA9, ABCA5, ABCA6, ABCA10, ABCA8, ABCA4, ABCA12, ABCA7 |
| **RNA:Dapma7bEVm000278** | 1.36 | COL21A1, COL19A1, COL9A1, COL22A1, COL16A1, COL16A1 |
| **RNA:Dapma7bEVm004069** | 1.35 | RAD21, RAD21L1 |
| **RNA:Dapma7bEVm007981** | 1.35 | TFDP1, TFDP2, TFDP3 |
| **RNA:Dapma7bEVm005743** | 1.35 | IFI30 |
| **RNA:Dapma7bEVm002383** | 1.35 | EIF2S1 |
| **RNA:Dapma7bEVm000159** | 1.35 | ACE, ACE2 |
| **RNA:Dapma7bEVm000952** | 1.35 | CTSD, REN, CTSE, NAPSA |
| **RNA:Dapma7bEVm000290** | 1.35 | UBE3B, UBE3C |
| **RNA:Dapma7bEVm002026** | 1.35 | PPIA, PPIF, PPIE, PPIAL4A, PPIAL4E, PPIAL4F, PPIAL4G, PPIAL4C, LOC105371242, PPIAL4D |
| **RNA:Dapma7bEVm026426** | 1.35 | NA |
| **RNA:Dapma7bEVm027847** | 1.34 | ELOVL2, ELOVL5, ELOVL4 |
| **RNA:Dapma7bEVm011195** | 1.34 | RNF7, RBX1 |
| **RNA:Dapma7bEVm003956** | 1.34 | NA |
| **RNA:Dapma7bEVm015081** | 1.34 | SULE1, SULB1, SULA3, SULA2, SULA4, SULA1, SULT2B1, SULC3, SULT6B1, SULC2, SULC4, SULT2A1, SULT4A1 |
| **RNA:Dapma7bEVm000391** | 1.34 | WASF1, WASF3, WASF2 |
| **RNA:Dapma7bEVm029320** | 1.34 | PSMD10 |
| **RNA:Dapma7bEVm015127** | 1.33 | PRKCB, PRKCA, PRKCA, PRKCG |
| **RNA:Dapma7bEVm004890** | 1.33 | SNAP29 |
| **RNA:Dapma7bEVm001285** | 1.33 | ASTL |
| **RNA:Dapma7bEVm000739** | 1.33 | PSMC1, PSMC6, PSMC2, PSMC3, PSMC5, PSMC5, PSMC4 |
| **RNA:Dapma7bEVm010428** | 1.33 | PSMA8, PSMA7 |
| **RNA:Dapma7bEVm000671** | 1.33 | SLC6A6, SLC6A13, SLC6A12, SLC6A11, SLC6A1, SLC6A8 |
| **RNA:Dapma7bEVm006597** | 1.32 | NA |
| **RNA:Dapma7bEVm000438** | 1.32 | C4B, CD109, C4A, A2M, PZP, PZP, A2ML1, C5, CPAMD8, LOC110384692, C4B_2 |
| **RNA:Dapma7bEVm000579** | 1.32 | AQP9, AQP9, AQP3, LOC112267859, AQP7, AQP10, LOC100509620 |
| **RNA:Dapma7bEVm019181** | 1.32 | CES1, CES4A, LOC107987423 |
| **RNA:Dapma7bEVm003504** | 1.32 | COPA, COPB2 |
| **RNA:Dapma7bEVm001854** | 1.32 | DUSP6, DUSP10, DUSP7, DUSP9 |
| **RNA:Dapma7bEVm002760** | 1.32 | PSEN1, PSEN2 |
| **RNA:Dapma7bEVm000852** | 1.32 | ABCB6, ABCB7 |
| **RNA:Dapma7bEVm007431** | 1.32 | SMAD1, SMAD5, SMAD9, SMAD3, SMAD2, SMAD2 |
| **RNA:Dapma7bEVm003115** | 1.32 | HSPA9, HSPA5 |
| **RNA:Dapma7bEVm004942** | 1.31 | RAC1, RHOG, RAC3, RAC2 |
| **RNA:Dapma7bEVm002712** | 1.31 | PRKACG, PRKACB, PRKACB, PRKACA, PRKX |
| **RNA:Dapma7bEVm000593** | 1.31 | VWF, OTOGL, MUC19, SSPO, MUC2, MUC5B, OTOG, MUC6, MUC5AC |
| **RNA:Dapma7bEVm015028** | 1.31 | SLC2A12, SLC2A13, SLC2A12, SLC2A6, SLC2A8, SLC2A10 |
| **RNA:Dapma7bEVm005481** | 1.31 | MELTF, LTF, TF |
| **RNA:Dapma7bEVm005137** | 1.31 | ALPL, ALPG, ALPI, ALPP |
| **RNA:Dapma7bEVm009461** | 1.31 | EGFLAM, EGFLAM, EGFLAM, AGRN, HSPG2 |
| **RNA:Dapma7bEVm007803** | 1.3 | KCNJ8, KCNJ11, KCNJ1, KCNJ5, KCNJ18, KCNJ16, KCNJ12, KCNJ2, KCNJ9, KCNJ10, KCNJ3, KCNJ14, KCNJ15, KCNJ6, KCNJ4 |
| **RNA:Dapma7bEVm029234** | 1.3 | PECR |
| **RNA:Dapma7bEVm004864** | 1.3 | GTF2E2 |
| **RNA:Dapma7bEVm015441** | 1.3 | CFL2, CFL1, DSTN, DSTN |
| **RNA:Dapma7bEVm003201** | 1.3 | SLC37A4 |
| **RNA:Dapma7bEVm025316** | 1.3 | METTL24 |
| **RNA:Dapma7bEVm001786** | 1.29 | ACE, ACE2 |
| **RNA:Dapma7bEVm010462** | 1.29 | MAN2B2, MAN2B1 |
| **RNA:Dapma7bEVm010075** | 1.29 | LDHB, LDHAL6B, LDHC, LDHA, LDHAL6A |
| **RNA:Dapma7bEVm002783** | 1.29 | TP53, TP73, TP63 |
| **RNA:Dapma7bEVm003085** | 1.28 | EHHADH, HADHA |
| **RNA:Dapma7bEVm027564** | 1.28 | DLAT, PDHX |
| **RNA:Dapma7bEVm000246** | 1.27 | HEXB, HEXA |
| **RNA:Dapma7bEVm005258** | 1.27 | SDR42E2, HSD3B7, SDR42E1, HSD3B1, HSD3B2, NSDHL, SDR42E2 |
| **RNA:Dapma7bEVm000432** | 1.27 | FABP7, PMP2, FABP4, FABP9, FABP12, FABP5, RBP7, FABP3, RBP2 |
| **RNA:Dapma7bEVm010421** | 1.27 | PPP2CA, PPP2CB, PPP6C, PPP4C |
| **RNA:Dapma7bEVm015567** | 1.27 | LPAR2 |
| **RNA:Dapma7bEVm010304** | 1.27 | MSMO1, SC5D |
| **RNA:Dapma7bEVm027409** | 1.27 | GLB1L3, GLB1L2, GLB1L, GLB1 |
| **RNA:Dapma7bEVm011582** | 1.27 | TOMM20L, TOMM20 |
| **RNA:Dapma7bEVm012899** | 1.27 | SNRPG |
| **RNA:Dapma7bEVm003630** | 1.26 | INHBE, INHBC, INHBA, INHBB |
| **RNA:Dapma7bEVm027676** | 1.26 | PTK2, PTK2, PTK2B |
| **RNA:Dapma7bEVm000092** | 1.26 | SLC17A8, SLC17A6, SLC17A7 |
| **RNA:Dapma7bEVm006777** | 1.26 | MGA, POMGN |
| **RNA:Dapma7bEVm007501** | 1.26 | INMT, NNMT, PNMT |
| **RNA:Dapma7bEVm028972** | 1.26 | HSP90AB1, HSP90B1, HSP90AA1 |
| **RNA:Dapma7bEVm004741** | 1.26 | BECN1, BECN2 |
| **RNA:Dapma7bEVm015167** | 1.25 | GM2A |
| **RNA:Dapma7bEVm005063** | 1.25 | B3GALT4, B3GNT4, B3GNT6, B3GNT9, B3GNT3, B3GALT2, B3GNT7, B3GAL, B3GNT2, B3GALN, B3GNT8, B3GALT5 |
| **RNA:Dapma7bEVm002504** | 1.25 | PSMB1, PSMB3 |
| **RNA:Dapma7bEVm004673** | 1.25 | P4HA2, P4HA1, P4HA3 |
| **RNA:Dapma7bEVm011087** | 1.25 | COX6C |
| **RNA:Dapma7bEVm018485** | 1.25 | TOPBP1, ECT2 |
| **RNA:Dapma7bEVm001570** | 1.25 | PDHB, BCKDHB |
| **RNA:Dapma7bEVm015041** | 1.25 | ETS1, ETV2, ETS2, GABPA |
| **RNA:Dapma7bEVm011478** | 1.24 | ARF5, ARF1, ARF4 |
| **RNA:Dapma7bEVm000957** | 1.24 | ADH7, ADH1B, ADH6, ADH1A, ADH4, ADH5, ADH1C |
| **RNA:Dapma7bEVm003269** | 1.24 | ETF1 |
| **RNA:Dapma7bEVm027937** | 1.24 | H1FX, H1F0 |
| **RNA:Dapma7bEVm009540** | 1.24 | COL1A2, COL5A2, COL3A1 |
| **RNA:Dapma7bEVm009016** | 1.24 | NA |
| **RNA:Dapma7bEVm003476** | 1.24 | ABCA13, ABCA1, ABCA2, ABCA3, ABCA9, ABCA5, ABCA6, ABCA10, ABCA8, ABCA4, ABCA12, ABCA7 |
| **RNA:Dapma7bEVm002441** | 1.24 | SMN2, SMN1, SMNDC1 |
| **RNA:Dapma7bEVm028590** | 1.24 | CAT |
| **RNA:Dapma7bEVm004795** | 1.24 | ATF1, CREM, CREM, CREB1 |
| **RNA:Dapma7bEVm008058** | 1.24 | NA |
| **RNA:Dapma7bEVm028352** | 1.24 | ME1, ME3, ME2 |
| **RNA:Dapma7bEVm001773** | 1.23 | EXTL3, EX, EXT2, EXTL1, EXTL2 |
| **RNA:Dapma7bEVm005649** | 1.23 | ARFIP1, ARFIP2 |
| **RNA:Dapma7bEVm024419** | 1.22 | RAD54B, RAD54L |
| **RNA:Dapma7bEVm017067** | 1.22 | NA |
| **RNA:Dapma7bEVm007297** | 1.22 | ENOPH1 |
| **RNA:Dapma7bEVm005362** | 1.22 | MOB1B, MOB3B, MOB3C, MOB1A, MOB3A |
| **RNA:Dapma7bEVm004477** | 1.22 | SKP1 |
| **RNA:Dapma7bEVm007409** | 1.21 | NA |
| **RNA:Dapma7bEVm007373** | 1.21 | GTF2A2 |
| **RNA:Dapma7bEVm004109** | 1.21 | GLB1L3, GLB1L2, GLB1L, GLB1 |
| **RNA:Dapma7bEVm012383** | 1.21 | SQSTM1 |
| **RNA:Dapma7bEVm027942** | 1.21 | C1QBP |
| **RNA:Dapma7bEVm010533** | 1.2 | PSMA4 |
| **RNA:Dapma7bEVm010029** | 1.2 | SEC24D, SEC24C |
| **RNA:Dapma7bEVm010638** | 1.2 | MYL12A, MYL12B, MYL9 |
| **RNA:Dapma7bEVm005302** | 1.2 | NA |
| **RNA:Dapma7bEVm021641** | 1.19 | LIPN, LIPK, LIPJ, LIPK, LIPF, LIPM, LIPA |
| **RNA:Dapma7bEVm007354** | 1.19 | YKT6 |
| **RNA:Dapma7bEVm010343** | 1.19 | ACLY |
| **RNA:Dapma7bEVm000055** | 1.19 | FLNC, FLNA |
| **RNA:Dapma7bEVm015380** | 1.19 | EIF5 |
| **RNA:Dapma7bEVm001012** | 1.19 | VPS28 |
| **RNA:Dapma7bEVm015082** | 1.19 | SULE1, SULB1, SULA3, SULA2, SULA4, SULA1, SULT2B1, SULC3, SULT6B1, SULC2, SULC4, SULT2A1, SULT4A1 |
| **RNA:Dapma7bEVm011186** | 1.19 | NA |
| **RNA:Dapma7bEVm000985** | 1.19 | CA1, CA10, CA11 |
| **RNA:Dapma7bEVm004026** | 1.19 | FGB, FGL2, ANGPT2, ANGP, ANGPTL4, ANGPTL3, ANGPTL7, ANGPT4 |
| **RNA:Dapma7bEVm004380** | 1.19 | MTX3, MTX1, MTX2 |
| **RNA:Dapma7bEVm003049** | 1.18 | PSAPL1, PSAP, SFTPB |
| **RNA:Dapma7bEVm001499** | 1.18 | ALDH7A1 |
| **RNA:Dapma7bEVm009518** | 1.18 | TGM1, TGM7, EPB42, TGM5, F13A1, TGM4, TGM2, TGM3, TGM6, TGM4 |
| **RNA:Dapma7bEVm010456** | 1.18 | SEMA5A, SEMA4B, SEMA4B, SEMA4D, SEMA4A, SEMA4F, SEMA5B |
| **RNA:Dapma7bEVm001026** | 1.18 | COL4A1, COL4A2, COL4A3, COL4A3, COL4A4, COL4A5, COL4A6 |
| **RNA:Dapma7bEVm009567** | 1.17 | SCP2, SCP2, SCP2 |
| **RNA:Dapma7bEVm002259** | 1.17 | CDC45 |
| **RNA:Dapma7bEVm001472** | 1.17 | MMP19, MMP17, MMP14, MMP27, MMP3, MMP12, MMP10, MMP15, MMP25, MMP25, MMP28, MMP28, MMP11 |
| **RNA:Dapma7bEVm010774** | 1.17 | NA |
| **RNA:Dapma7bEVm015292** | 1.17 | ALG8, ALG6 |
| **RNA:Dapma7bEVm007334** | 1.17 | GCH1 |
| **RNA:Dapma7bEVm002257** | 1.17 | SARS, SARS2 |
| **RNA:Dapma7bEVm008948** | 1.17 | NA |
| **RNA:Dapma7bEVm004236** | 1.17 | BMP4, BMP2 |
| **RNA:Dapma7bEVm004984** | 1.16 | NA |
| **RNA:Dapma7bEVm007173** | 1.16 | CDA |
| **RNA:Dapma7bEVm018740** | 1.16 | ERN2, ERN1 |
| **RNA:Dapma7bEVm004148** | 1.16 | DUSP6, DUSP10, DUSP7, DUSP9 |
| **RNA:Dapma7bEVm009691** | 1.16 | TUBA1C, TUBA1A, TUBA1B, TUBA3C, TUBAL3, TUBA4A, TUBA3D, TUBA3E, TUBA8 |
| **RNA:Dapma7bEVm017437** | 1.16 | NA |
| **RNA:Dapma7bEVm003338** | 1.16 | RACGAP1 |
| **RNA:Dapma7bEVm018811** | 1.15 | KCNK16, KCNK10, KCNK4, KCNK2 |
| **RNA:Dapma7bEVm000191** | 1.15 | TPX2 |
| **RNA:Dapma7bEVm028045** | 1.15 | SLC5A8, SLC5A12, SLC5A6, SLC5A5 |
| **RNA:Dapma7bEVm013111** | 1.15 | SRP9 |
| **RNA:Dapma7bEVm004157** | 1.15 | PGM5, PGM1 |
| **RNA:Dapma7bEVm004797** | 1.15 | VEGFC, VEGFA, PGF, VEGFB, VEGFD |
| **RNA:Dapma7bEVm007428** | 1.15 | CASP2, CASP2, CASP14, CASP9 |
| **RNA:Dapma7bEVm000083** | 1.15 | STAT6, STAT2, STAT3, STAT5B, STAT5A, STA, STAT4 |
| **RNA:Dapma7bEVm023719** | 1.14 | SDR42E2, HSD3B7, SDR42E1, HSD3B1, HSD3B2, NSDHL, SDR42E2 |
| **RNA:Dapma7bEVm011670** | 1.14 | SNRPD3 |
| **RNA:Dapma7bEVm001680** | 1.14 | PIK3C2G, PIK3C2G, PIK3C2A, PIK3C2B |
| **RNA:Dapma7bEVm004644** | 1.14 | TPTE2, PTEN, PTEN |
| **RNA:Dapma7bEVm008666** | 1.14 | NA |
| **RNA:Dapma7bEVm007633** | 1.14 | PI16, GLIPR1, GLIPR1L2, GLIPR1L1, CRISP3, CRISP2, CRISP1, PI15, CRISPLD1, CLEC18B, CRISPLD2, CLEC18A, CLEC18C, R3HDML |
| **RNA:Dapma7bEVm003075** | 1.14 | NUP62, NUP62CL |
| **RNA:Dapma7bEVm010078** | 1.14 | YWHAG, YWHAZ, YWHAE, SFN, YWHAQ, YWHAB, YWHAH |
| **RNA:Dapma7bEVm000346** | 1.14 | MAP3K4, MAP3K19 |
| **RNA:Dapma7bEVm019300** | 1.14 | NA |
| **RNA:Dapma7bEVm001464** | 1.13 | KARS |
| **RNA:Dapma7bEVm002347** | 1.13 | PTGES2 |
| **RNA:Dapma7bEVm011308** | 1.13 | NA |
| **RNA:Dapma7bEVm022115** | 1.13 | TUBA1C, TUBA1A, TUBA1B, TUBA3C, TUBAL3, TUBA4A, TUBA3D, TUBA3E, TUBA8 |
| **RNA:Dapma7bEVm003529** | 1.13 | DDX46, DDX42 |
| **RNA:Dapma7bEVm009841** | 1.13 | PHKG1, PHKG2 |
| **RNA:Dapma7bEVm027262** | 1.13 | TLR9, TLR2, TLR6, TLR1, TLR3, TLR10, CD180, TLR4, TLR4, TLR5, TLR7, TLR8 |
| **RNA:Dapma7bEVm009587** | 1.13 | AQP9, AQP9, AQP3, LOC112267859, AQP7, AQP10, LOC100509620 |
| **RNA:Dapma7bEVm002143** | 1.13 | LIMK1, TESK1, TESK2, LIMK2, LIMK2 |
| **RNA:Dapma7bEVm011408** | 1.13 | PTGS1, PTGS2 |
| **RNA:Dapma7bEVm023023** | 1.12 | NUDT21 |
| **RNA:Dapma7bEVm023914** | 1.12 | TFAP2B, TFAP2A, TFAP2D, TFAP2E, TFAP2C |
| **RNA:Dapma7bEVm006170** | 1.12 | TOPBP1, ECT2 |
| **RNA:Dapma7bEVm004664** | 1.12 | CEL |
| **RNA:Dapma7bEVm004558** | 1.12 | MCM9, MCM3, MCM7, MCM9, MCM4, MCM6, MCM2, MCM8, MCM5 |
| **RNA:Dapma7bEVm012412** | 1.12 | FABP6, FABP1 |
| **RNA:Dapma7bEVm000785** | 1.12 | XRN1, XRN1, XRN2 |
| **RNA:Dapma7bEVm000543** | 1.12 | FLAD1 |
| **RNA:Dapma7bEVm009653** | 1.12 | CYP3A7-CYP3A51P, TBXAS1, CYP3A4, CYP3A43, CYP3A7, TBXAS1, CYP3A5 |
| **RNA:Dapma7bEVm019488** | 1.12 | NA |
| **RNA:Dapma7bEVm023649** | 1.12 | ABCG2, ABCG8, ABCG5 |
| **RNA:Dapma7bEVm009543** | 1.12 | HIF1A, EPAS1, HIF3A |
| **RNA:Dapma7bEVm005621** | 1.11 | BIRC2, BIRC3, BIRC8, BIRC7, XIAP |
| **RNA:Dapma7bEVm006235** | 1.11 | ATG4C, ATG4B, ATG4D, ATG4A |
| **RNA:Dapma7bEVm008074** | 1.11 | CLEC19A, REG4, REG1B, REG3G, REG3A, REG1A, CLEC3B, CLEC4M |
| **RNA:Dapma7bEVm007743** | 1.11 | NA |
| **RNA:Dapma7bEVm003664** | 1.11 | RPN1 |
| **RNA:Dapma7bEVm010754** | 1.11 | POLR1C |
| **RNA:Dapma7bEVm009806** | 1.11 | THBS4, THBS2, THBS1, COMP, THBS3 |
| **RNA:Dapma7bEVm004134** | 1.1 | LMNB1, LMNA, LMNB2 |
| **RNA:Dapma7bEVm001525** | 1.1 | CALR3, CALR |
| **RNA:Dapma7bEVm004259** | 1.1 | VPS4A, VPS4B |
| **RNA:Dapma7bEVm001497** | 1.1 | SPDEF |
| **RNA:Dapma7bEVm009549** | 1.1 | RASA1, SYNGAP1, DAB2IP, DAB2IP, RASAL2, RASAL2, RASAL2, RASAL3 |
| **RNA:Dapma7bEVm006122** | 1.1 | PARK7 |
| **RNA:Dapma7bEVm010828** | 1.1 | CTBP1, CTBP2, PHGDH |
| **RNA:Dapma7bEVm002558** | 1.1 | FANCI |
| **RNA:Dapma7bEVm005467** | 1.1 | SEL1L, SEL1L2 |
| **RNA:Dapma7bEVm005405** | 1.1 | EIF1AX, EIF1AY |
| **RNA:Dapma7bEVm022974** | 1.1 | UGT2B28, UGT2B15, UGT2A3, UGT2B4, UGT2B11, UGT2B7, UGT2B17, UGT2A1, UGT8, UGT2B10, UGT3A2, UGT3A1, UGA7, UGA10, UGA4, UGA8, UGA9, UGA1, UGA6, UGA3, UGA5, LOC101929773, UGT2A3 |
| **RNA:Dapma7bEVm010216** | 1.09 | CTNND2, CTNND1, PKP3, PKP4, PKP4, ARVCF |
| **RNA:Dapma7bEVm004554** | 1.09 | GABBR1, GABBR2, GABBR1 |
| **RNA:Dapma7bEVm005949** | 1.09 | INHBE, INHBC, INHBA, INHBB |
| **RNA:Dapma7bEVm010250** | 1.09 | SLC2A9, SLC2A9, SLC2A14, SLC2A3, SLC2A4, SLC2A7, SLC2A1, SLC2A5, SLC2A2, SLC2A11 |
| **RNA:Dapma7bEVm015109** | 1.09 | IGF2BP3, IGF2BP1, IGF2BP2 |
| **RNA:Dapma7bEVm006210** | 1.09 | PAPSS1, PAPSS2 |
| **RNA:Dapma7bEVm007162** | 1.09 | NHP2 |
| **RNA:Dapma7bEVm000984** | 1.08 | RPSAP58, RPSA |
| **RNA:Dapma7bEVm003920** | 1.08 | REELD1 |
| **RNA:Dapma7bEVm001123** | 1.08 | RACGAP1 |
| **RNA:Dapma7bEVm004411** | 1.08 | AQP9, AQP9, AQP3, LOC112267859, AQP7, AQP10, LOC100509620 |
| **RNA:Dapma7bEVm006493** | 1.08 | CHRNA9, CHRFAM7A, CHRNA7, CHRNA7, CHRNA10 |
| **RNA:Dapma7bEVm005385** | 1.07 | NA |
| **RNA:Dapma7bEVm030410** | 1.07 | KIF4B, KIF21A, KIF7, KIF27, KIF21B, KIF4A |
| **RNA:Dapma7bEVm010820** | 1.07 | SLC13A4, SLC13A4, SLC13A1, SLC13A2, SLC13A5, SLC13A3 |
| **RNA:Dapma7bEVm005142** | 1.07 | PTPN2, PTPN1, PTPN1 |
| **RNA:Dapma7bEVm000442** | 1.07 | NPC1L1, NPC1 |
| **RNA:Dapma7bEVm009911** | 1.07 | ALDOB, ALDOA, ALDOC |
| **RNA:Dapma7bEVm027599** | 1.07 | HDAC10, HDAC6 |
| **RNA:Dapma7bEVm005064** | 1.07 | NCAPH |
| **RNA:Dapma7bEVm012397** | 1.07 | UGT2B28, UGT2B15, UGT2A3, UGT2B4, UGT2B11, UGT2B7, UGT2B17, UGT2A1, UGT8, UGT2B10, UGT3A2, UGT3A1, UGA7, UGA10, UGA4, UGA8, UGA9, UGA1, UGA6, UGA3, UGA5, LOC101929773, UGT2A3 |
| **RNA:Dapma7bEVm005078** | 1.06 | CDC16 |
| **RNA:Dapma7bEVm004353** | 1.06 | HCCS |
| **RNA:Dapma7bEVm018376** | 1.06 | ATP6V0E1, ATP6V0E2 |
| **RNA:Dapma7bEVm010265** | 1.06 | DPP7, PRCP |
| **RNA:Dapma7bEVm003039** | 1.06 | PSMD2 |
| **RNA:Dapma7bEVm009468** | 1.06 | ATP12A, ATP1A4, ATP1A1, ATP1A2, ATP4A, ATP1A3 |
| **RNA:Dapma7bEVm015652** | 1.06 | RPS27L, RPS27 |
| **RNA:Dapma7bEVm001283** | 1.06 | PCNA |
| **RNA:Dapma7bEVm005267** | 1.06 | WDR61 |
| **RNA:Dapma7bEVm000487** | 1.06 | SEPT7, SEP, SEP2, C17orf47, SEPT9, SEPT9, SEPT4, SEPT9, SEPT2, SEPT5, SEPT5, SEPT3 |
| **RNA:Dapma7bEVm000388** | 1.06 | PRKG2, PRKG1, PRKG1 |
| **RNA:Dapma7bEVm020394** | 1.06 | MT-CO2 |
| **RNA:Dapma7bEVm015025** | 1.06 | P4HA2, P4HA1, P4HA3 |
| **RNA:Dapma7bEVm003126** | 1.06 | ABCG2, ABCG8, ABCG5 |
| **RNA:Dapma7bEVm005978** | 1.06 | SULE1, SULB1, SULA3, SULA2, SULA4, SULA1, SULT2B1, SULC3, SULT6B1, SULC2, SULC4, SULT2A1, SULT4A1 |
| **RNA:Dapma7bEVm007536** | 1.06 | VBP1 |
| **RNA:Dapma7bEVm018600** | 1.06 | XRCC5 |
| **RNA:Dapma7bEVm024100** | 1.06 | RAD54B, RAD54L |
| **RNA:Dapma7bEVm004943** | 1.05 | RAB4A, RAB4B |
| **RNA:Dapma7bEVm006440** | 1.05 | ACP1 |
| **RNA:Dapma7bEVm010819** | 1.05 | FZD1, FZD3, FZD3, FZD6, FZD2, FZD7 |
| **RNA:Dapma7bEVm028906** | 1.05 | MAN2B2, MAN2B1 |
| **RNA:Dapma7bEVm004624** | 1.05 | PPARD, NR1D1, PPARG, NR1D2, PPARA |
| **RNA:Dapma7bEVm016561** | 1.05 | RGS3, RGS1, RGS16, RGS2, RGS21, RGS8, RGS5, RGS13, RGS18, RGS4 |
| **RNA:Dapma7bEVm006094** | 1.05 | DNAJB9 |
| **RNA:Dapma7bEVm027787** | 1.04 | UGT2B28, UGT2B15, UGT2A3, UGT2B4, UGT2B11, UGT2B7, UGT2B17, UGT2A1, UGT8, UGT2B10, UGT3A2, UGT3A1, UGA7, UGA10, UGA4, UGA8, UGA9, UGA1, UGA6, UGA3, UGA5, LOC101929773, UGT2A3 |
| **RNA:Dapma7bEVm010257** | 1.04 | NA |
| **RNA:Dapma7bEVm028503** | 1.04 | NA |
| **RNA:Dapma7bEVm011534** | 1.04 | CES1, CES4A, LOC107987423 |
| **RNA:Dapma7bEVm015247** | 1.04 | GCLC |
| **RNA:Dapma7bEVm023068** | 1.04 | EPHX1 |
| **RNA:Dapma7bEVm010808** | 1.04 | EIF3CL, EIF3C |
| **RNA:Dapma7bEVm011228** | 1.04 | PNKP |
| **RNA:Dapma7bEVm001510** | 1.04 | SLC7A11, SLC7A8, SLC7A7, SLC7A6, SLC7A5 |
| **RNA:Dapma7bEVm001697** | 1.03 | CLCA2, CLCA4, CLCA1 |
| **RNA:Dapma7bEVm011641** | 1.03 | HS6ST3, HS6S, HS6ST2, HS6ST2 |
| **RNA:Dapma7bEVm007672** | 1.03 | PSME2, PSME1, PSME3 |
| **RNA:Dapma7bEVm000188** | 1.03 | ABCG2, ABCG8, ABCG5 |
| **RNA:Dapma7bEVm001183** | 1.03 | CCNB1, CCNB2 |
| **RNA:Dapma7bEVm007019** | 1.03 | KYA, KYAT3 |
| **RNA:Dapma7bEVm008142** | 1.03 | SERPINB1, SERPINB9, SERPINE3, SERPINA4, SERPINA6, SERPINA9, SERPINB6, SERPINE1, SERPINH1, SERPINB13, SERPINB2, SERPINB11, SERPINB12, SERPINB7, SERPINB5, SERPINB4, SERPINB8, SERPINB10, HMSD, SERPINB3, SERPINC1, SERPINE2, SERPINI2, SERPINI1, SERPIND1 |
| **RNA:Dapma7bEVm004291** | 1.03 | PPP1CC, PPP1CA, PPP1CB |
| **RNA:Dapma7bEVm003143** | 1.03 | ZNF501, ZNF197, ZBTB11, ZNF141, PRDM5, ZBTB49, ZNF732, ZNF595, ZNF718, ZSCAN26, ZSCAN2, ZNF165, ZNF12, ZNF853, ZNF736, ZNF398, ZNF705D, ZNF510, ZNF33A, ZNF239, ZFP2, ZFP62, ZNF10, ZNF705A, ZNF655, ZNF777, ZNF273, ZNF789, ZNF572, ZNF707, ZNF883, ZNF37A, ZNF721, ZNF605, ZNF770, ZNF391, ZNF783, ZNF425, ZKSCAN1, ZFP41, ZNF705G, ZNF79, ZNF484, ZNF300, ZSCAN9, ZNF268, ZNF322, ZNF786, ZNF775, REPIN1, ZNF727, ZNF623, LOC107984126, ZNF25, ZNF214, ZNF354C, ZNF140, ZNF26, ZBTB24, ZNF398, ZNF716, ZSCAN25, ZNF7, ZNF705B, ZFAT, ZNF248, ZNF354B, ZSCAN16, ZSCAN12, ZNF184, RBAK, ZKSCAN5, ZNF3, ZNF517, ZNF596, ZNF189, ZNF485, ZNF215, ZNF454, ZNF84, ZSCAN29, ZFP57, ZNF713, ZNF479, ZNF679, ZNF7, ZNF696, ZNF658, ZNF202, ZNF141, ZNF354A, ZNF641, ZSCAN23, ZNF394, ZNF138, ZNF212, ZNF92, ZNF250, ZNF483, ZNF169, ZNF33A, ZNF879, ZNF664, ZNF774, ZNF311, ZNF735, ZNF107, ZSCAN21, ZNF251, ZNF16, ZNF782, ZNF32, PRDM9, ZSCAN31, ZNF26, ZKSCAN8, ZNF107, ERV3-1-ZNF117, ZNF467, ZNF680, GLI4, ZFP37, ZNF22, ZNF33B, ZNF891, ZNF316, ZNF282, ZNF746, ZNF34, ZNF169, ZSCAN30, ZNF589, ZNF446, HKR1, ZNF20, ZNF229, ZSCAN1, ZNF157, ZSCAN32, ZNF93, ZNF559, ZBTB48, ZNF134, ZNF43, ZNF256, ZBTB32, ZNF793, ZNF835, ZNF133, PRDM11, ZNF597, ZNF778, ZNF114, ZNF302, ZNF17, ZNF667, ZNF781, ZNF558, ZNF296, ZNF788P, ZNF335, ZNF23, ZNF232, ZNF546, ZNF806, ZNF660, ZNF429, ZNF223, ZNF85, ZNF471, ZNF331, ZNF808, LOC112268403, ZNF345, ZNF695, ZNF619, ZNF350, ZNF506, ZNF254, ZNF45, ZNF431, ZNF324B, ZNF674, ZNF780A, ZNF785, ZNF174, ZNF497, ZNF684, ZNF675, ZNF419, ZNF431, ZNF528, ZNF628, ZNF439, ZNF57, ZNF626, ZNF195, ZFP3, ZNF678, ZNF613, ZNF816, ZNF175, ZNF568, ZNF610, ZNF121, ZNF253, ZNF341, ZNF688, ZNF397, ZNF543, ZFP69B, ZNF620, ZNF574, ZNF347, ZNF556, ZNF730, ZNF257, LOC107984139, PATZ1, ZNF689, ZNF222, ZKSCAN7, ZNF846, ZNF749, ZNF699, ZNF765-ZNF761, ZNF805, ZNF91, ZNF780B, ZNF311, E4F1, ZNF19, ZNF730, ZNF496, ZNF813, PEG3, ZNF625, ZIK1, ZNF792, ZNF257, ZIM2, ZFP1, ZNF48, ZNF594, ZNF492, ZNF589, ZNF567, ZNF790, ZNF780A, ZNF551, ZFP28, ZNF584, PATZ1, ZNF286A, ZNF285, ZNF670, ZNF570, ZNF616, ZNF155, ZNF490, ZNF284, ZNF845, ZNF534, ZNF334, ZNF630, ZNF213, ZNF24, ZNF8, ZNF860, ZNF468, ZNF571, ZNF723, ZNF544, ZNF583, MZF1, ZNF563, ZNF705E, ZNF205, ZNF254, ZNF648, ZNF14, ZFP14, ZNF557, ZNF101, ZNF791, ZNF112, ZNF611, ZNF587B, ZNF747, ZNF519, ZNF493, ZNF35, ZNF283, ZNF527, ZNF491, ZNF564, ZNF841, ZNF480, ZNF275, ZNF263, ZNF846, ZBTB40, ZNF80, ZNF358, ZSCAN22, ZNF442, ZNF540, ZFP30, ZNF708, ZNF324, ZFP92, ZNF646, ZNF286A, ZNF181, ZNF142, ZNF415, ZNF787, ZNF681, ZNF677, ZNF221, ZNF587, GZF1, ZNF771, ZNF627, ZNF418, ZNF586, ZNF320, ZNF582, ZNF560, ZNF460, ZNF614, ZNF773, ZNF780B, ZNF85, ZNF200, ZNF430, ZBTB41, MYNN, ZNF234, ZNF529, ZNF230, ZNF562, ZNF177, ZNF404, ZNF70, ZFP90, ZNF407, ZNF136, ZNF691, ZIM3, ZNF585B, ZNF726, ZNF573, ZNF233, ZNF738, ZNF426, ZNF133, ZNF764, ZNF624, ZNF566, ZNF621, ZNF160, ZNF765, ZNF784, ZNF98, ZNF333, ZNF788P, ZNF449, ZBTB16, ZFP1, ZNF554, ZNF669, ZNF66, ZNF888, ZNF473, ZNF304, ZNF878, ZNF260, ZNF544, ZNF28, ZNF287, ZNF429, ZNF514, ZNF662, ZNF30, ZNF776, ZNF555, ZNF665, ZNF682, ZNF133, LOC101928592, ZNF319, ZNF552, ZSCAN20, ZNF225, ZNF550, ZNF549, ZNF763, ZNF547, ZNF132, ZNF600, ZNF69, ZNF251, ZNF668, ZNF500, ZBTB17, ZNF461, ZNF266, LOC105372319, ZNF211, ZNF274, ZNF606, ZNF729, ZNF561, ZNF408, PRDM7, ZNF236, ZNF578, ZNF717, ZSCAN18, ZNF146, ZNF559, ZNF470, ZNF585A, FIZ1, ZNF182, ZSCAN10, ZNF99, ZFP69, ZNF852, ZNF224, ZNF135, ZNF253, ZNF565, ZNF579, ZNF343, ZNF41, ZNF267, ZNF396, ZNF823, ZNF445, ZNF837, ZNF90, ZNF44, ZNF814, ZNF829, ZNF569, ZNF737, ZNF705G, ZNF267, MAZ, ZNF77, ZNF672, ZNF180, ZNF264, ZNF728, ZNF865, ZNF700, ZNF420, ZNF382, ZNF844, ZNF18, ZNF227, ZNF2, ZNF329, ZNF548, ZNF880, ZNF607, ZNF615, ZNF443, PRDM15, ZNF546, ZNF629, ZKSCAN2, ZNF83, ZNF697, ZNF799, ZNF526, ZNF486, ZNF530, ZNF649, ZNF671, ZNF525, ZNF433, ZNF236, ZNF766, LOC101928592, ZNF57, ZNF440, ZNF441, ZNF416, ZNF709, ZNF100, ZNF836, ZNF74, ZNF768, ZNF286B, ZNF71, ZNF436, ZNF432, ZNF850, ZNF772, ZNF235, ZNF577, ZNF417, ZNF724, ZNF337, ZFP90, ZNF154, ZNF124, ZNF502, ZNF208, ZNF599, ZNF383, ZNF226, ZNF317, ZFP82, ZNF81, ZNF701 |
| **RNA:Dapma7bEVm029516** | 1.03 | NA |
| **RNA:Dapma7bEVm003278** | 1.03 | PSMC1, PSMC6, PSMC2, PSMC3, PSMC5, PSMC5, PSMC4 |
| **RNA:Dapma7bEVm010516** | 1.03 | RPS2 |
| **RNA:Dapma7bEVm027494** | 1.03 | TGFBI, POSTN |
| **RNA:Dapma7bEVm006501** | 1.02 | MAP2K4, LOC100996792, MAP2K4, MAP2K4, MAP2K6, MAP2K3, MAP2K6, MAP2K7 |
| **RNA:Dapma7bEVm000301** | 1.02 | ABCG2, ABCG8, ABCG5 |
| **RNA:Dapma7bEVm005521** | 1.02 | STXBP1, STXBP3, STXBP2 |
| **RNA:Dapma7bEVm010425** | 1.02 | PRSS1, PRSS2, PRSS1, PRSS3, PRSS3, KLK9, KLK10, PRSS38, KLK3, KLK4, KLK12, AZU1, PRTN3, ELANE, KLK11, KLK15 |
| **RNA:Dapma7bEVm002619** | 1.02 | TTK |
| **RNA:Dapma7bEVm026291** | 1.02 | NA |
| **RNA:Dapma7bEVm003779** | 1.01 | CCNB1, CCNB2 |
| **RNA:Dapma7bEVm000591** | 1.01 | HPGDS |
| **RNA:Dapma7bEVm006767** | 1.01 | NEK4, NEK11, NEK1, NEK5, NEK3, NEK9, NEK8, NEK2, NEK2, NEK4, NEK11, NEK4 |
| **RNA:Dapma7bEVm000171** | 1.01 | LPIN2, LPIN1, LPIN1, LPIN1, LPIN3 |
| **RNA:Dapma7bEVm015143** | 1.01 | SLC27A4, SLC27A1 |
| **RNA:Dapma7bEVm007546** | 1.01 | NA |
| **RNA:Dapma7bEVm005939** | 1.01 | B4GAL, B4GALT6, B4GALT2, B4GALT3, B4GALT4, B4GALT5 |
| **RNA:Dapma7bEVm010497** | 1.01 | PTGER4, PTGER2, PTGDR, PTGFR, PTGER3, PTGER3, PTGER1, TBXA2R, PTGIR |
| **RNA:Dapma7bEVm028591** | 1.01 | RTL1, LOC107984745, LOC112267992, LOC105379356, C8orf33, LOC105376526, LOC105373926, LOC105374103, LOC105376906 |
| **RNA:Dapma7bEVm011047** | 1.01 | SGPP1, SGPP2, SGPP2 |
| **RNA:Dapma7bEVm010261** | 1.01 | ALDH6A1 |
| **RNA:Dapma7bEVm001890** | 1.01 | SLC7A11, SLC7A8, SLC7A7, SLC7A6, SLC7A5 |
| **RNA:Dapma7bEVm023195** | 1.01 | UBE3B, UBE3C |
| **RNA:Dapma7bEVm001673** | 1 | TCF12, TCF4, TCF3 |
| **RNA:Dapma7bEVm003092** | 1 | CASP8, CASP10 |
| **RNA:Dapma7bEVm000560** | 1 | CPOX |
| **RNA:Dapma7bEVm003418** | 1 | FAXC |
| **RNA:Dapma7bEVm005313** | 1 | PGAM2, BPGM, PGAM1 |
| **RNA:Dapma7bEVm001745** | 1 | HYOU1, HYOU1 |
| **RNA:Dapma7bEVm011333** | 1 | ACE, ACE2 |
| **RNA:Dapma7bEVm030441** | 1 | NA |


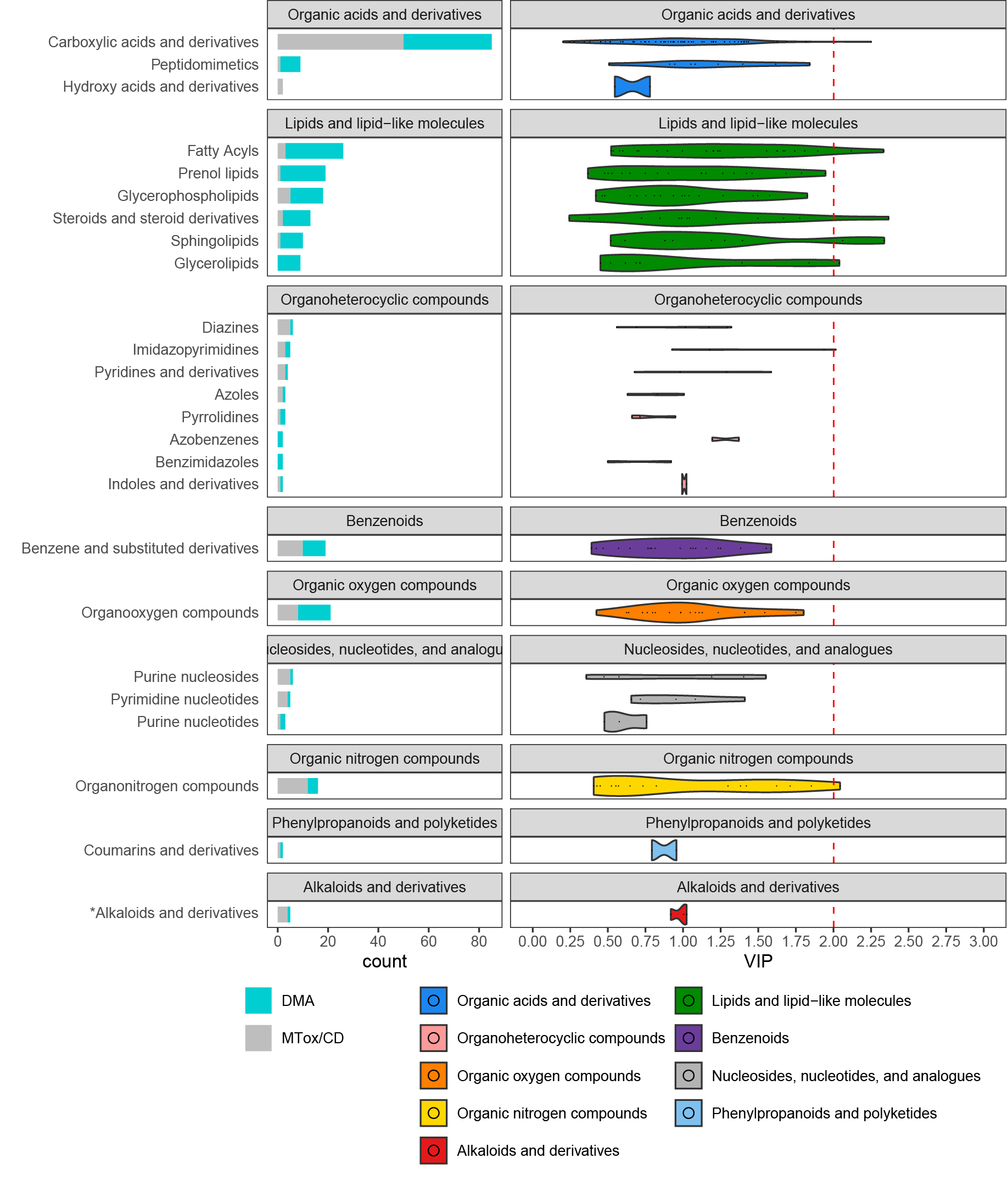


Figure S5. Top 25 most prevalent compound classes observed (and their corresponding superclasses). Superclasses in grey boxes and classes listed in each row. Alkaloids and derivatives are only classified to a superclass. Left side: Total count across all assays, colour denotes if the annotation was observed in either “MTox/CD” (where annotations were derived from either the MTox700+ spectral library or from Compound Discoverer), “DMA” (where annotations were derived from the DMA Galaxy workflow). Right side: Violin plots for each of the top 25 most prevalent compound classes of the VIP scores derived from PLS-DA model. Dotted red line is shown at a threshold of VIP score of 2. *These alkaloids and derivatives annotations did not have a specific compound class name (only superclass, kingdom and annotation to a specific compound identifier).

##### Section S5 - Further investigation of glycolysis pathway

To further investigate the perturbation of glycolysis in *D. magna* is response to phthalates and uncouplers, the transcriptomics data were searched for other members of the glycolysis pathway. A total of five *D. magna* genes were orthologous to the human glycolysis enzymes *GPI, PFK, ALDO, PGK* and *ENO* (Figure S6; Table S8). Enolase (*ENO*) showed reduced expression across all substances however none of these changes were statistically significant. In addition to *PFK*, expression of *GPI* and *ALDO* were also significantly up-regulated in FCCP-treated samples although *ALDO* was also significantly upregulated in the phthalate DiBP. Except for *GPI,* the expression pattern in both uncouplers showed a similar trend, although the changes were not statistically significant in any of the genes following TCP exposure. Several studies have reported changes in glycolysis following exposure to uncouplers, including significant increases in glycolytic intermediate metabolite levels in rat primary hepatocytes (Fujimoto *et al.*, 2020) and Chinese hamster lung fibroblasts (Balcke *et al.*, 2011) following short-term exposure to FCCP, which has been suggested to represent an enhancement of glycolysis to fulfil cellular energy requirements and produce ATP, in response to the inhibition of oxidative phosphorylation.

Using G2P-SCAN to investigate the conservation of the human glycolysis pathway (defined in the Reactome database; R-HSA-70171) across multiple species (Figure S6) revealed that this pathway is highly conserved particularly at the level of reactions and protein families with 67% and 80% conservation in the fruit fly *Drosophila melanogaster*. Indeed, the ten enzymatic steps of glycolysis are central to cellular energy metabolism and are ubiquitous within eukaryotes (Fothergill-Gilmore and Michels, 1993).


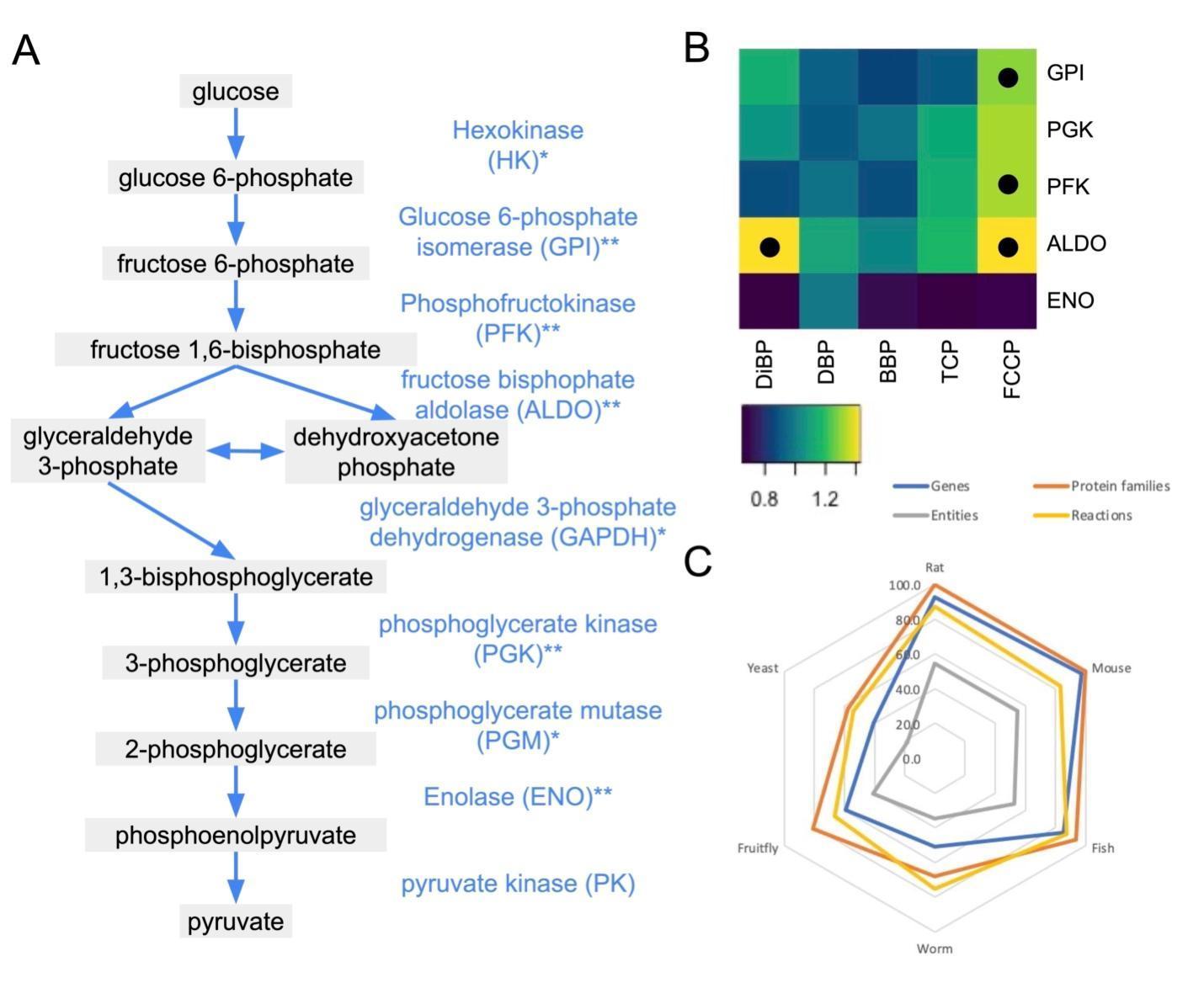


Figure S6. (A) Glycolysis metabolic pathway and catalytic enzymes (blue), *indicates presence of homologous genes in Daphnia magna, **indicates genes represented in this study. (B) Heatmap indicates the mean fold-changes of Daphnia magna (5 d) treated samples following high dose exposure to test substances benzyl butyl phthalate (BBP), dibutyl phthalate (DBP), diisobutyl phthalate (DiBP), 2, 3, 4, 5-tetrachlorophenol (TCP) and carbonyl cyanide 4-(trifluoromethoxy)phenylhydrazone (FCCP). Black circles indicate significant (q<0.05) changes relative to control samples. (C) Percentage conservation of human glycolysis pathway across model species obtained using “Genes to Pathway - species conservation analysis tool” (G2P-SCAN; Rivetti et al., 2023). Full G2P-SCAN results are presented in Table S8.

Table S8. Mapping Daphnia magna genes to human orthologs and Reactome pathways using “Genes to Pathway - species conservation analysis tool” (G2P-SCAN; Rivetti et al., 2023). Target genes were selected based on the top-ranked VIP score derived from a PLS-DA model following high dose treatment of the test substances benzyl butyl phthalate (BBP), dibutyl phthalate (DBP), diisobutyl phthalate (DiBP), 2, 3, 4, 5-tetrachlorophenol (TCP) and carbonyl cyanide 4-(trifluoromethoxy)phenylhydrazone (FCCP) and manual searching for those related to glycolysis.

| ***Daphnia magna* gene ID** | **Human gene ID** | **VIP score** | **Rank order (total features)** | **Rank order (genes)** | **Uniprot ID** | **Uniprot description** | **Terminal Reactome pathway ID** | **Pathway** |
| --- | --- | --- | --- | --- | --- | --- | --- | --- |
| Dapma7bEVm009578 | PFKM | 2.333 | 111 | 1 | A0A0P6AZG6 | ATP-dependent 6-phosphofructokinase | R-HSA-70171 | Glycolysis |
| Dapma7bEVm006728 | ACHE | 2.227 | 277 | 2 | A0A0P4XHD6 | Acetylcholinesterase | R-HSA-112311 | Neurotransmitter clearance |
|  |  |  |  |  |  |  | R-HSA-1483191 | Synthesis of PC |
|  |  |  |  |  |  |  | R-HSA-422085 | Synthesis, secretion, and deacylation of Ghrelin |
| Dapma7bEVm000940 | TUBB | 2.192 | 365 | 3 | A0A0P5AM59 | Tubulin beta chain | R-HSA-8854518 | AURKA Activation by TPX2 |
|  |  |  |  |  |  |  | R-HSA-5620912 | Anchoring of the basal body to the plasma membrane |
|  |  |  |  |  |  |  | R-HSA-380259 | Loss of Nlp from mitotic centrosomes |
|  |  |  |  |  |  |  | R-HSA-6798695 | Neutrophil degranulation |
|  |  |  |  |  |  |  | R-HSA-9679191 | Potential therapeutics for SARS |
|  |  |  |  |  |  |  | R-HSA-380320 | Recruitment of NuMA to mitotic centrosomes |
|  |  |  |  |  |  |  | R-HSA-380270 | Recruitment of mitotic centrosome proteins and complexes |
|  |  |  |  |  |  |  | R-HSA-2565942 | Regulation of PLK1 Activity at G2/M Transition |
| Dapma7bEVm001565 | CPB2 | 2.180 | 401 | 4 | A0A0P6B1I0 | Carboxypeptidase B | R-HSA-2022377 | Metabolism of Angiotensinogen to Angiotensins |
|  |  |  |  |  |  |  | R-HSA-977606 | Regulation of Complement cascade |
| Dapma7bEVm001991 | EPHX2 | 2.173 | 419 | 5 | A0A0P6BSV0 | Epoxide hydrolase |  |  |
| Dapma7bEVm001910 | CNDP2 | 2.159 | 457 | 6 | A0A0P5RA14 | Cytosolic non-specific dipeptidase | R-HSA-174403 | Glutathione synthesis and recycling |
|  |  |  |  |  |  |  | R-HSA-9753281 | Paracetamol ADME |
| Dapma7bEVm010420 | RCN2 | 2.138 | 514 | 7 | A0A0P5UAS3 | Calumenin-B |  |  |
| Dapma7bEVm001597 | PPA2 | 2.118 | 572 | 8 | A0A0P6I4V8 | Inorganic pyrophosphatase | R-HSA-379726 | Mitochondrial tRNA aminoacylation |
|  |  |  |  |  |  |  | R-HSA-71737 | Pyrophosphate hydrolysis |
| Dapma7bEVm025783 | NA | 2.106 | 624 | 9 | A0A0P6I5G5 | A0A0P6I5G5_9CRUS |  |  |
| Dapma7bEVm004847 | UGT2B28 | 2.101 | 640 | 10 | A0A0P6J2I1 | Glucosyl/glucuronosyl transferases | R-HSA-9749641 | Aspirin ADME |
|  |  |  |  |  |  |  | R-HSA-156588 | Glucuronidation |
| Dapma7bEVm004767 | PGK2 | 2.016 | 937 | 15 | A0A0P6ITA9 | Phosphoglycerate kinase | R-HSA-70263 | Gluconeogenesis |
|  |  |  |  |  |  |  | R-HSA-70171 | Glycolysis |
| Dapma7bEVm009911 | ALDOB | 1.067 | 12670 | 387 | A0A0P6DE75 | Fructose-bisphosphate aldolase | R-HSA-70350 | Fructose catabolism |
|  |  |  |  |  |  |  | R-HSA-70263 | Gluconeogenesis |
|  |  |  |  |  |  |  | R-HSA-70171 | Glycolysis |
|  |  |  |  |  |  |  | R-HSA-5657560 | Hereditary fructose intolerance |
| Dapma7bEVm003660 | GPI | 0.973 | 15591 | 502 | A0A0P5RLF6 | Glucose-6-phosphate isomerase | R-HSA-70263 | Gluconeogenesis |
|  |  |  |  |  |  |  | R-HSA-70171 | Glycolysis |
|  |  |  |  |  |  |  | R-HSA-6798695 | Neutrophil degranulation |
|  |  |  |  |  |  |  | R-HSA-5628897 | TP53 Regulates Metabolic Genes |
| Dapma7bEVm015145 | ENO2 | 0.442 | 38223 | 1527 | A0A0P5TRX3 | Alpha-enolase | R-HSA-70263 | Gluconeogenesis |
|  |  |  |  |  |  |  | R-HSA-70171 | Glycolysis |

Table S9. Summary of results from “Genes to Pathway - species conservation analysis tool” (G2P-SCAN; Rivetti et al., 2023) for the glycolysis pathway (R-HSA-70171 from the Reactome database) showing the total number of genes, proteins, protein families, entities, and reactions across 6 model species.

|  | **Human** | **Rat  (*R. norvegicus*)** | | **Mouse (*M. musculus*)** | | **Fish  (*D. rerio*)** | | **Worm  (*C. elegans*)** | | **Fruit fly  (*D. melanogaster*)** | | **Yeast  (*S. cerevisiae*)** | |
| --- | --- | --- | --- | --- | --- | --- | --- | --- | --- | --- | --- | --- | --- |
|  |  | **Count** | **%** | **Count** | **%** | **Count** | **%** | **Count** | **%** | **Count** | **%** | **Count** | **%** |
| **Total Genes** | 69 | 64 | 92.8 | 67 | 97.1 | 59 | 85.5 | 35 | 50.7 | 41 | 59.4 | 28 | 40.6 |
| **Protein Count** | 69 | 64 | 92.8 | 67 | 1.0 | 59 | 85.5 | 35 | 50.7 | 41 | 59.4 | 28 | 40.6 |
| **Protein Families** | 47 | 47 | 100.0 | 47 | 1.0 | 44 | 93.6 | 32 | 68.1 | 38 | 80.9 | 27 | 57.4 |
| **Entities** | 110 | 60 | 54.5 | 60 | 0.5 | 58 | 52.7 | 38 | 34.5 | 45 | 40.9 | 20 | 18.2 |
| **Reactions** | 24 | 21 | 87.5 | 20 | 0.8 | 21 | 87.5 | 18 | 75.0 | 16 | 66.7 | 13 | 54.2 |

##### References

Balcke, G. U., Kolle, S. N., Kamp, H., Bethan, B., Looser, R., Wagner, S., Landsiedel, R., & van Ravenzwaay, B. (2011). Linking energy metabolism to dysfunctions in mitochondrial respiration – A metabolomics in vitro approach. Toxicology Letters, 203(3), 200–209. <https://doi.org/10.1016/j.toxlet.2011.03.013>

Durant J. L., Leland B. A., Henry D. R., Nourse J. G. (2002). Reoptimization of MDL keys for use in drug discovery. J. Chem. Inf. Comput. Sci. 42, 1273–1280. <https://doi.org/10.1021/ci010132r>

Dührkop, K., Fleischauer, M., Ludwig, M., Aksenov, A. A., Melnik, A. V., Meusel, M., Dorrestein, P. C., Rousu, J., & Böcker, S. (2019). SIRIUS 4: a rapid tool for turning tandem mass spectra into metabolite structure information. Nature Methods, 16(4), 299–302. <https://doi.org/10.1038/s41592-019-0344-8>

Fothergill-Gilmore, L. A., & Michels, P. A. M. (1993). Evolution of glycolysis. Progress in Biophysics and Molecular Biology, 59(2), 105–235. <https://doi.org/10.1016/0079-6107(93)90001-Z>

Ji, H., Deng, H., Lu, H., & Zhang, Z. (2020). Predicting a molecular fingerprint from an electron ionization mass spectrum with deep neural networks. Analytical Chemistry, 92(13), 8649-8653. <https://doi.org/10.1021/acs.analchem.0c01450>

Lloyd, G. R., Jankevics, A., & Weber, R. J. M. (2020). Struct: An R/Bioconductor-based framework for standardized metabolomics data analysis and beyond. Bioinformatics, 36(22–23), 5551–5552. <https://doi.org/10.1093/bioinformatics/btaa1031>

Rivetti, C., Houghton, J., Basili, D., Hodges, G., & Campos, B. (2023). Genes‐to‐Pathways Species Conservation Analysis: Enabling the Exploration of Conservation of Biological Pathways and Processes Across Species. Environmental Toxicology and Chemistry, 42(5), 1152–1166. <https://doi.org/10.1002/etc.5600>

Rogers, D., & Hahn, M. (2010). Extended-Connectivity Fingerprints. J. Chem. Inf. and Model. 50, 742-54. <https://doi.org/10.1021/ci100050t>

Ruttkies, C., Schymanski, E. L., Wolf, S., Hollender, J., & Neumann, S. (2016). MetFrag relaunched: Incorporating strategies beyond in silico fragmentation. Journal of Cheminformatics, 8, 3. <https://doi.org/10.1186/s13321-016-0115-9>

Southam, A. D., Pursell, H., Frigerio, G., Jankevics, A., Weber, R. J. M., & Dunn, W. B. (2021). Characterization of Monophasic Solvent-Based Tissue Extractions for the Detection of Polar Metabolites and Lipids Applying Ultrahigh-Performance Liquid Chromatography-Mass Spectrometry Clinical Metabolic Phenotyping Assays. Journal of Proteome Research, 20(1), 831–840. <https://doi.org/10.1021/acs.jproteome.0c00660>
